# A novel bicistronic vector inducing balanced alternative splicing for antibody production

**DOI:** 10.1038/s41598-026-57819-1

**Published:** 2026-06-11

**Authors:** Shoutaro Tsuji

**Affiliations:** Department of Medical Technology & Clinical Engineering, Gunma University of Health and Welfare, 191-1 Kawamagari-machi, Maebashi, 371-0823 Gunma Japan

**Keywords:** Alternative splicing, Bicistronic expression, Cassette exon, CHO cells, HEG1, Recombinant antibody, Biochemistry, Biological techniques, Biotechnology, Cell biology

## Abstract

Efficient and stable production of recombinant monoclonal antibodies requires sufficient expression of light (LC) and heavy (HC) chains within host cells. Conventional bicistronic systems such as internal ribosome entry site (IRES)- or 2 A peptide–based vectors often suffer from imbalanced translation efficiencies or the presence of residual peptide tags that can compromise antibody quality. We developed a novel bicistronic expression platform, designated the cassette exon–associated (CAE) system, which exploits alternative splicing derived from the HEG1 gene to generate two mRNA isoforms from a single promoter. The CAE cassette contains synthetic splice donor and acceptor sequences that enable expression of LC and HC without additional peptide sequences. The optimized construct, pCAE3.31, driven by various promoters—including chicken β-actin, cytomegalovirus, and human elongation factor-1α—efficiently expressed functional antibodies in multiple mammalian cell lines. Compared with IRES-based systems, the CAE vector achieved an 8-fold higher antibody yield. Fab fragments were also produced using the CAE system. The CAE system represents a compact and versatile bicistronic expression strategy that ensures expression of LC and HC, avoids extraneous peptide fusion, and is compatible with major mammalian promoters. This platform facilitates efficient production of recombinant antibodies and Fab fragments, supporting screening and potential therapeutic applications.

**Supplementary Information:**

The online version contains supplementary material available at 10.1038/s41598-026-57819-1.

## Introduction

Recombinant monoclonal antibodies (mAbs) have become a cornerstone of modern biopharmaceuticals used in clinical practice^1^. Efficient production of mAbs requires the establishment of clones with both high productivity and stable expression^2^. The frequency of obtaining highly productive clones and the durability of expression depend on the number and chromosomal location of inserted transgenes driven by appropriate promoters. In the absence of site-specific integration technologies, the insertion site is largely stochastic. Smaller transgene constructs generally enhance transfection and nuclear import efficiency^3^, indicating that transgene size directly influences the likelihood of isolating high-producing clones.

Balanced biosynthesis of light (LC) and heavy (HC) chains within a cell is essential for efficient assembly of functional antibodies^4^. However, when LC and HC genes are introduced separately, the probability that both are highly expressed and stably integrated is relatively low. To overcome this limitation, bi-promoter vectors—harboring two independent promoters for LC and HC expression—are frequently employed. Nonetheless, the large size of bi-promoter vectors reduces transfection efficiency and complicates plasmid construction and purification. In addition, imbalance expression due to promoter silencing or transcriptional interference can impair antibody yield. Bicistronic vectors, in which two coding sequences are expressed under the control of a single promoter, represent an attractive alternative to achieve high productivity and stable expression with smaller vector size.

The internal ribosome entry site (IRES) is a classical element enabling bicistronic expression. IRES sequences recruit ribosomes in a 5′ cap–independent manner^5,6^, allowing translation of two open reading frames (ORFs) from a single mRNA—one from the initial AUG and another following the IRES^7^. Viral IRES elements, particularly those from picornaviruses, are well characterized and widely used in bicistronic reporter systems^8^. Although many cellular IRESs have been also identified, they typically function only under stress conditions and exhibit low translational efficiency^9^. In viral or cellular IRESs, the expression of the downstream ORF is markedly lower (< 20%) than that of the upstream ORF^10^, making IRES-based systems suboptimal for antibody production. While some reports have demonstrated stable mAb expression using IRES^11^, optimization is often antibody clone-specific, and the relatively long IRES sequence (> 500 bp) provides little advantage in reducing vector size compared with bi-promoter systems.

Another well-known bicistronic approach employs viral 2 A peptides. When a 2 A sequence (~ 20 amino acids) is inserted between two ORFs, ribosomal skipping during translation produces two separate polypeptides^7,12^. Although 2 A-based systems can be more efficient than IRES-based ones for antibody expression, residual peptide tags at the junction can promote aggregation and impair antibody quality^13^. Incomplete cleavage can also lead to fusion products and reduced expression of downstream chains^14^.

A different bicistronic mechanism utilizes alternative splicing, where two distinct 3′ splice acceptors generate separate mRNAs from a single transcript^15^. Adjusting the acceptor sequence can modulate expression ratios of the two mRNAs. This method introduces no additional sequence and avoids peptide fusions, suggesting potential suitability for antibody expression. However, expression balance is sensitive to both cistron and splice acceptor sequences, and the system’s productivity for antibodies remains to be fully characterized^15^. In the studies, authors have also pointed out that splice acceptor–like motifs located within coding regions can lead to unintended cryptic splicing events. For example, the presence of a splice acceptor motif within the LC coding region downstream of a 5′UTR intron has been reported to generate cryptic splicing^15^. Such splice acceptor motifs within open reading frames represent a recognized risk for unintended splicing events that can reduce protein expression. However, the extent to which these cryptic splicing events influence recombinant protein production has not been systematically investigated. This issue is not limited to alternative splicing–based vector systems but should be considered more broadly in the design of expression vectors that contain introns. Careful design of splice elements is therefore critical when developing intron-containing expression.

**Fig. 1:**
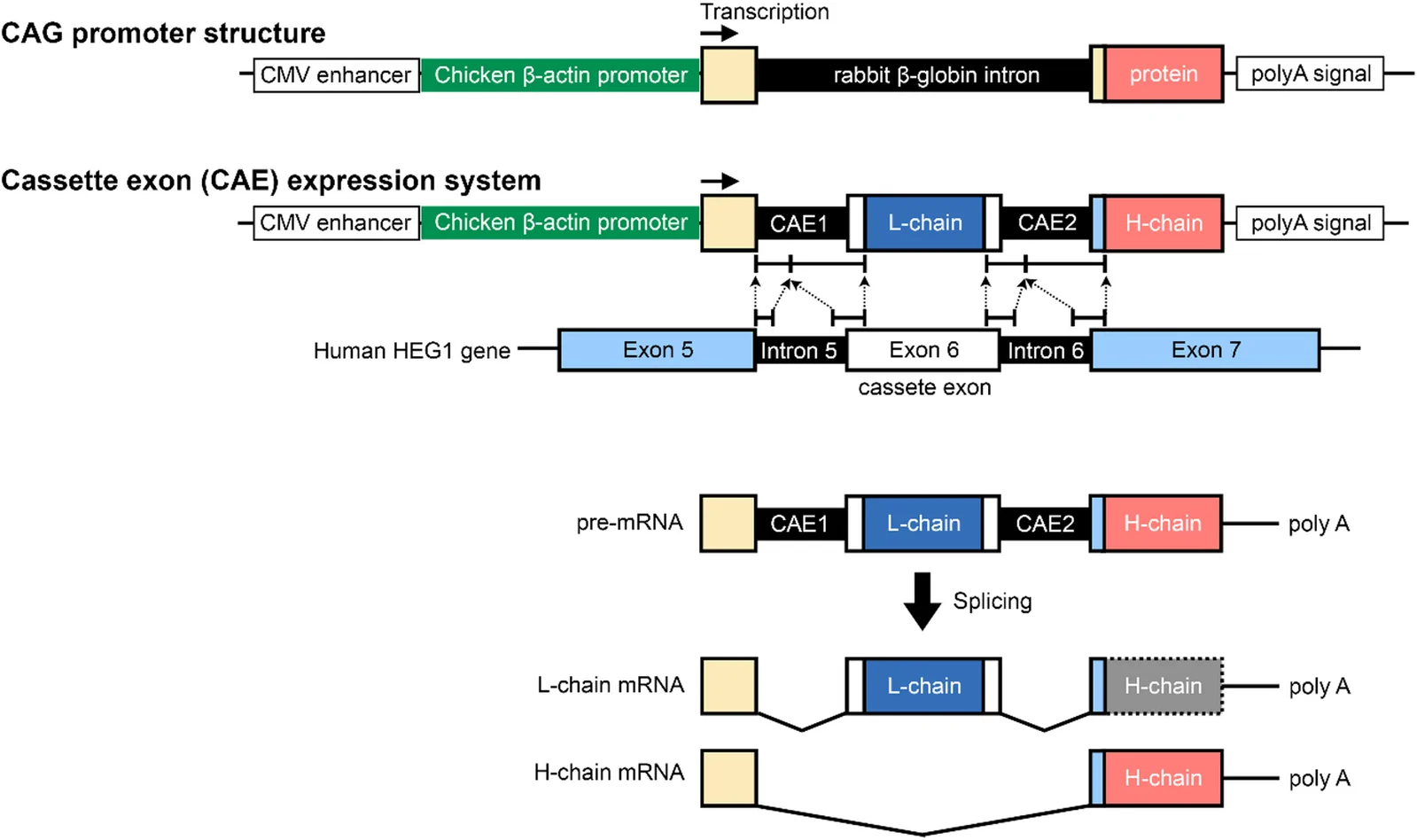
Bicistronic expression system using HEG1 introns. CAE1 contains HEG1 intron 5-derived splicing donor and acceptor, while CAE2 contains HEG1 intron 6-derived splicing donor and acceptor.

In this study, we developed a novel bicistronic expression system that enables transcription of two mRNAs in comparable amounts from a single promoter by inserting a short sequence (~ 140 bp) between two coding regions. The design was inspired by the alternative splicing of the *HEG homolog 1* (HEG1) gene, a mucin-like membrane protein expressed in mesothelial and endothelial cells. Exon 6 of *HEG1* functions as a cassette exon, generating two mRNA isoforms—one containing exon 6 and one lacking it—in approximately equal proportions across various tissues^16^. We hypothesized that introducing the *HEG1* exon 6 and its flanking intronic sequences between two antibody coding sequences could similarly yield balanced bicistronic expression (Fig. 1). We designated this intronic region as **CAE (CAssette Exon)** and established a new CAE-based bicistronic vector system. Using CAE cassette, various recombinant antibodies and fragments could be readily expressed by exchanging a ~ 1.3 kb synthetic gene containing LC (~ 750 bp), CAE (~ 140 bp), and VH regions (~ 400 bp) into an expression vector harboring the HC constant domain. The CAE system was compatible with several major promoters and efficiently supported antibody expression. In addition, the CAE system was able to efficiently produce Fab fragments. With the arrival of heterodimeric bispecific antibody drugs^1^, accurate affinity evaluation of monomeric antibodies in screening has become increasingly important, growing the demand for rapid and simple generation of recombinant Fab monomers. This simple yet effective design offers a practical tool for high-yield production of recombinant antibodies in research and development of antibody drug.

## Results

### Construction of CAE expression plasmids

The conceptual framework of the CAE bicistronic expression system is illustrated in Fig. 1. Exon 6 of *HEG1* functions as a cassette exon, producing two mRNA isoforms—one containing exon 6 and the other lacking it—in approximately equal proportions across multiple tissues^16^. Although the detailed mechanism underlying this balanced alternative splicing remains unclear, we hypothesized that two distinct mRNAs could be transcribed at similar levels by exploiting the splice donor and acceptor sites in *HEG1* introns 5 and 6 (Fig. 1).

**Fig. 2:**
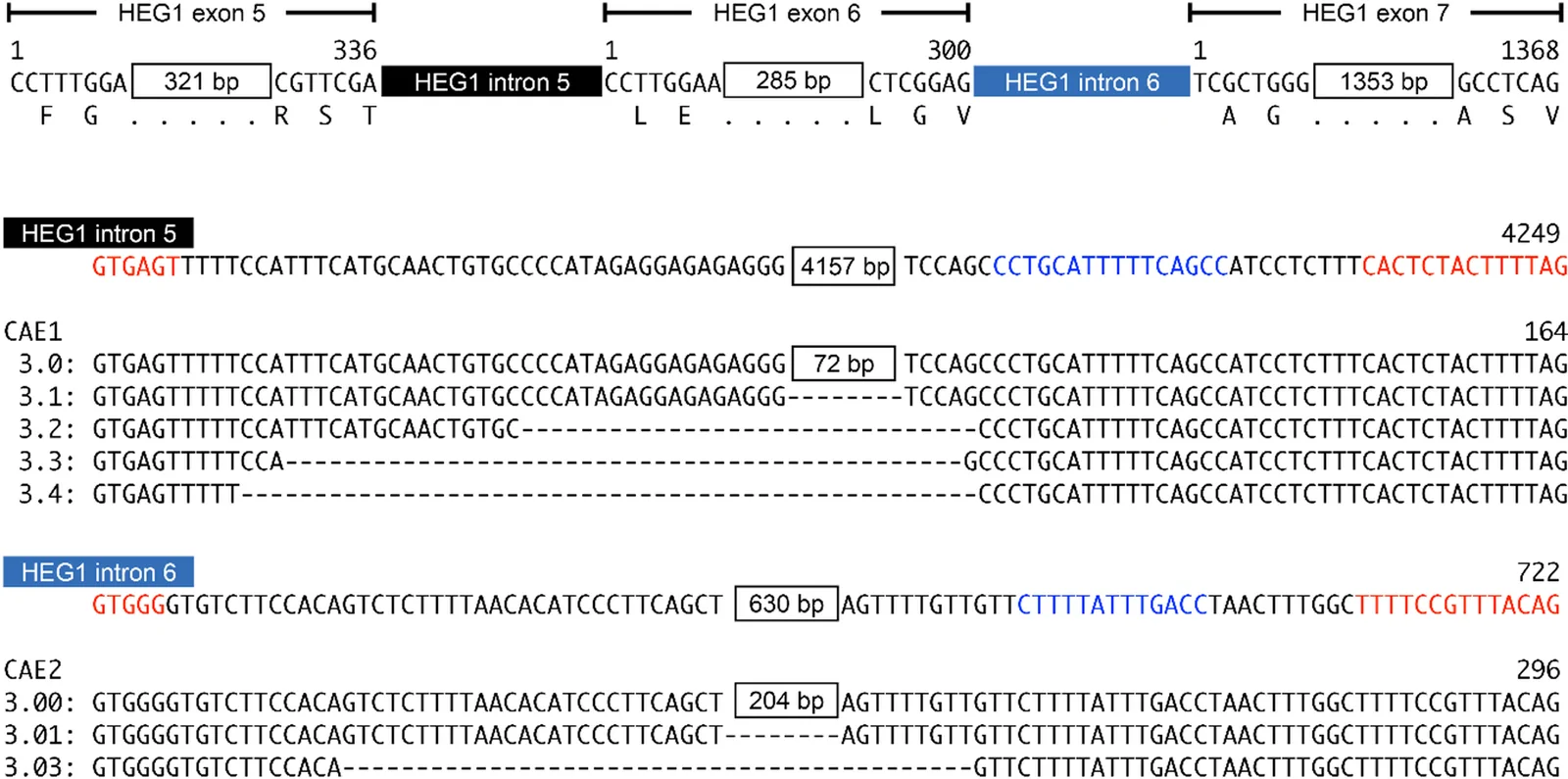
HEG1 gene structure surrounding exon 6 and CAE sequences. Red sequences indicate splicing donors or acceptors; blue sequences indicate putative branch sites. CAE1_3.0 and CAE2_3.00 contain spacer regions derived from HEG1 introns 14 and 11, respectively. HEG1 gene sequences were retrieved from GenBank (accession no. NC_000003, region 124965710–125055997). Exon 5 (336 bp) is located at 36,041–36,736, intron 5 is 4249 bp, exon 6 (300 bp) is located at 40,986–41,285, intron 6 is 722 bp, and exon 7 (1368 bp) is located at 42,008–43,375. Sequences of spacers, exons, and introns are shown in Supplementary Data 1.

In the design of the CAE system, the LC transcript includes the HC coding region downstream of the LC stop codon; however, HC translation is not expected because its initiation codon is located far downstream from the 5′ cap. To minimize intronic length while retaining function, we constructed synthetic introns by linking the proximal 5′ splice donor (47 bp) and distal 3′ splice acceptor (45 bp) regions of *HEG1* intron 5, which contains predicted branch sites (Fig. 2, CAE1_3.1). Another variant (CAE1_3.0) incorporated a short spacer derived from *HEG1* intron 14 between these elements. Similarly, CAE2_3.01 was generated from *HEG1* intron 6 by joining the 5′ donor (43 bp) and 3′ acceptor (49 bp) regions, and CAE2_3.00 included an additional spacer sequence from intron 11 (Fig. 2). Sequences of spacers, exons, and introns are provided in Supplementary Data 1.

**Fig. 3:**
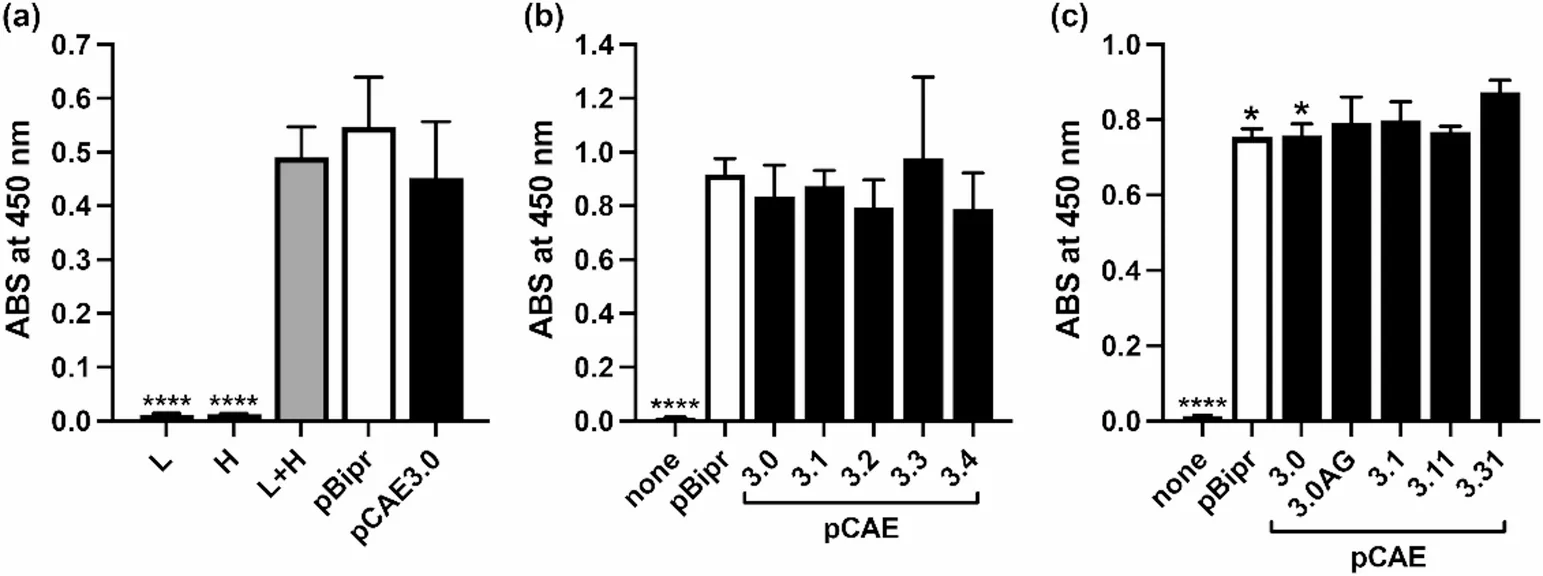
Construction and evaluation of pCAE plasmids. Antibody expression was measured by ELISA. Values in the figures represent mean ± SD of triplicates. The measurement range of the ELISA is shown as a representative standard curve in Supplementary Data 9. The representative standard curve confirmed that the absorbance measurements fell within the linear detection range of this assay. (**a**) Active antibody expression by CAE expression system. L or H is a transfection sample of pCXN2 (CAG promoter) containing the LC or pEF-BOS (EF-1α promoter) containing the HC, respectively. L + H is a sample in which both the LC and HC plasmids were co-transfected. pBipr is a sample of the original bi-promoter plasmid vector, in which the expression of the LC or HC is driven by the CAG promoter or the EF-1α promoter. pCAE3.0 is a sample of a newly constructed expression plasmid including CAE1_3.0 and CAE2_3.00 (chicken β-actin (CB) promoter). The plasmid maps of the used vectors and the inserted antibody sequences are shown in Supplementary Data 2. Statistical significance was determined by Dunnett’s one-way ANOVA. The control group was pCAE3.0. ****P < 0.0001. (**b**) Comparison of pCAE3.0-based mutants with replaced CAE1 region. As CAE2 region in the mutants, CAE2_3.00 was used. The horizontal axis numbers (3.0–3.4) indicate the replaced CAE1 sequences. Sample of pBipr was used as a positive control. Similar results were obtained in two independent experiments. The figure shows representative results. Statistical significance was determined by Dunnett’s one-way ANOVA. The control group was pCAE3.3. ****P < 0.0001. (**c**) Analysis of truncated CAE mutants. The horizontal axis numbers indicate the following CAE sequence combinations: 3.0, CAE1_3.0+CAE2_3.00; 3.1, CAE1_3.1+CAE2_3.00; 3.11, CAE1_3.1+CAE2_3.01; 3.31, CAE1_3.3+CAE2_3.01. 3.0AG indicates pCAE3.0 in which the immediately upstream sequences (CGA) of CAE1 were replaced with CAG. pBipr was used as positive control. Similar results were obtained in two independent experiments. The figure shows representative results. Statistical significance was determined by one-way ANOVA followed by Tukey’s multiple comparison test. ****P < 0.0001 and *P < 0.05 compared to pCAE3.31.

To apply the CAE system to antibody expression, we replaced the β-globin intron in the CAG promoter with CAE1_3.0, followed by an exon containing LC, CAE2_3.00, and HC sequences, generating plasmid pCAE3.0 (Figs. 1 and 2). Detailed plasmid information is provided in Supplementary Data 2. Transfection of pCAE3.0 into mammalian cells resulted in secretion of functional antibodies at levels comparable to those obtained with co-transfection of separate LC + HC plasmids or a conventional bi-promoter vector (pBipr) (Fig. 3a).

To identify more compact and efficient CAE elements, we analyzed truncated CAE1 variants (Fig. 2, CAE1_3.1–3.4). Among these, CAE1_3.3 modestly enhanced antibody production (Fig. 3b, pCAE3.3). Replacement of the exon 5 terminal sequences “CGA” with a more canonical splice boundary “CAG”^17^ did not significantly alter expression levels (Fig. 3c, 3.0AG). The inclusion or removal of the spacer within CAE2 had negligible impact (Fig. 3c, pCAE3.1 or pCAE3.11). Combining the optimal CAE1_3.3 and CAE2_3.01 elements yielded pCAE3.31, which slightly improved antibody secretion compared with earlier constructs (Fig. 3c).

### Analysis of CAE-mediated transcripts

**Fig. 4:**
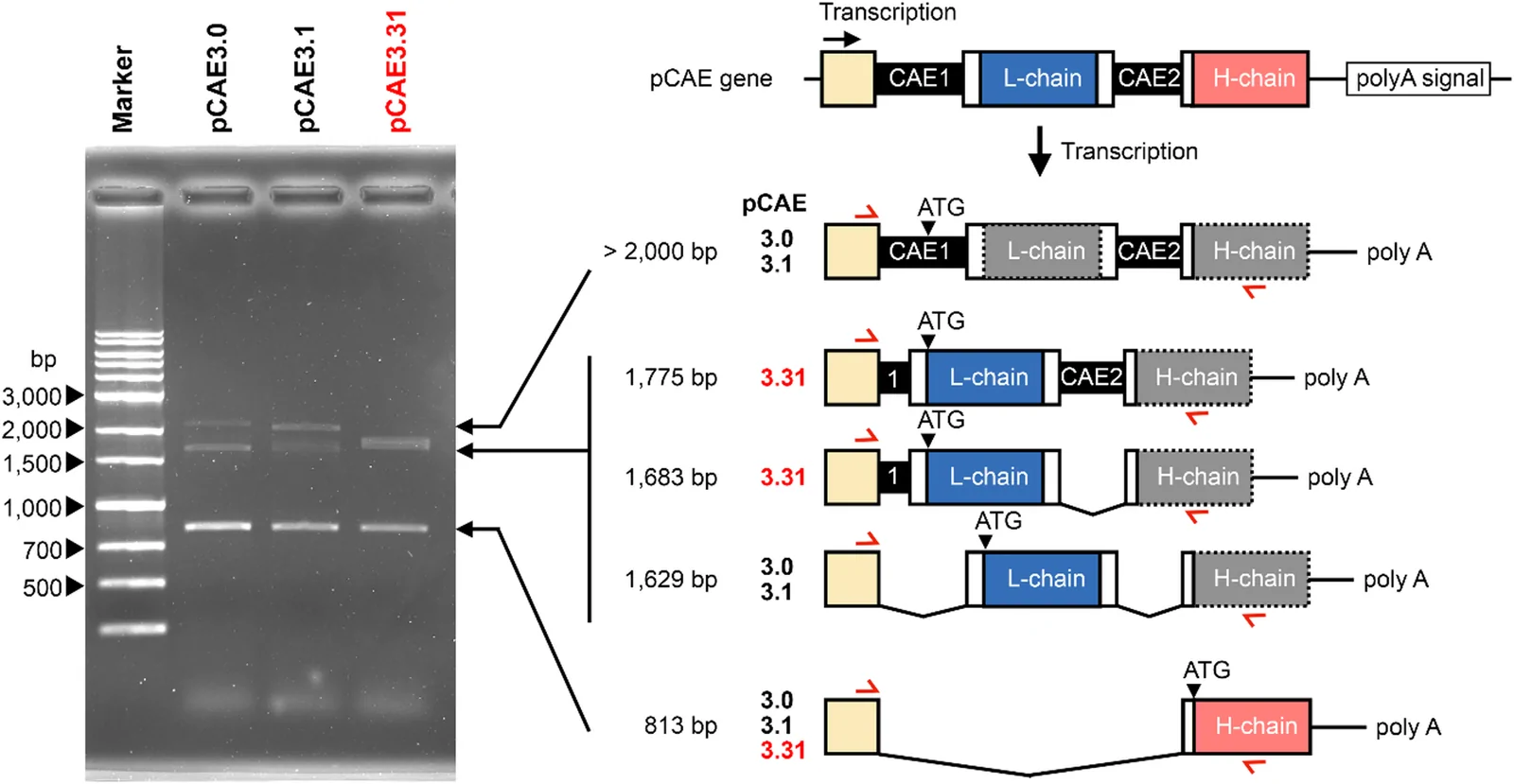
Splicing variants from pCAE plasmids. RT-PCR products from pCAE3.0, pCAE3.1, and pCAE3.31-transfected cells were analyzed. Red half-arrows indicate primer positions; grey dotted boxes indicate untranslated ORFs. Main PCR products were cloned and sequenced (Supplementary Data 3). Full-length gel image is presented in Supplementary Data 11.

To examine transcript profiles, total RNA was isolated from cells transfected with pCAE3.0, pCAE3.1, or pCAE3.31 and analyzed by RT-PCR. All constructs generated two or three major transcripts: a long form corresponding to LC mRNA and a shorter form corresponding to HC mRNA (Fig. 4). Sequencing confirmed that the 813 bp shorter product represented the designed HC transcript lacking the LC coding sequence (Supplementary Data 3).

In pCAE3.0 and pCAE3.1, the middle-length LC transcript (1,629 bp) exhibited the designed deletion of CAE1 and CAE2 regions, but approximately 25–30% of clones contained alternatively spliced variants with truncated LC C-termini due to unintended 5′ splice sites (Supplementary Data 3). In contrast, pCAE3.31 produced long LC mRNAs of 1,683 bp and 1,775 bp, corresponding to retention of CAE1_3.3 with or without CAE2. Because CAE1_3.3 lacks an initiation codon, its inclusion does not interfere with LC translation. Among 16 sequenced clones from long LC mRNAs of pCAE3.31, no aberrant splice variants lacking complete LC ORFs were detected, suggesting improved mRNA integrity and explaining the enhanced antibody expression. In pCAE3.0 and pCAE3.1, > 2,000 bp of long products contained both CAE1 and CAE2. The template mRNAs for these long products are not expected to translate LC because the CAE1 regions of pCAE3.0 and pCAE3.1 contain an initiation codon. The longer transcripts containing CAE2 (pCAE3.0 and pCAE3.1, > 2 kb; pCAE3.31, 1,775 bp) may represent un-spliced pre-mRNA species.

To further characterize transcript expression in the CAE system, the LC/HC mRNA ratio in pCAE3.31 was evaluated by real-time quantitative PCR. In pCAE3.31 driven by the CMVs promoter (see the section on Promoter Compatibility), the LC/HC transcript ratio was calculated to be 3.21 ± 0.28 (Supplementary Data 4). These results indicate that the CAE system produced both LC and HC transcripts from a single promoter at an appropriate transcription ratio while maintaining efficient antibody expression.

### Optimization of CAE sequences

**Fig. 5:**
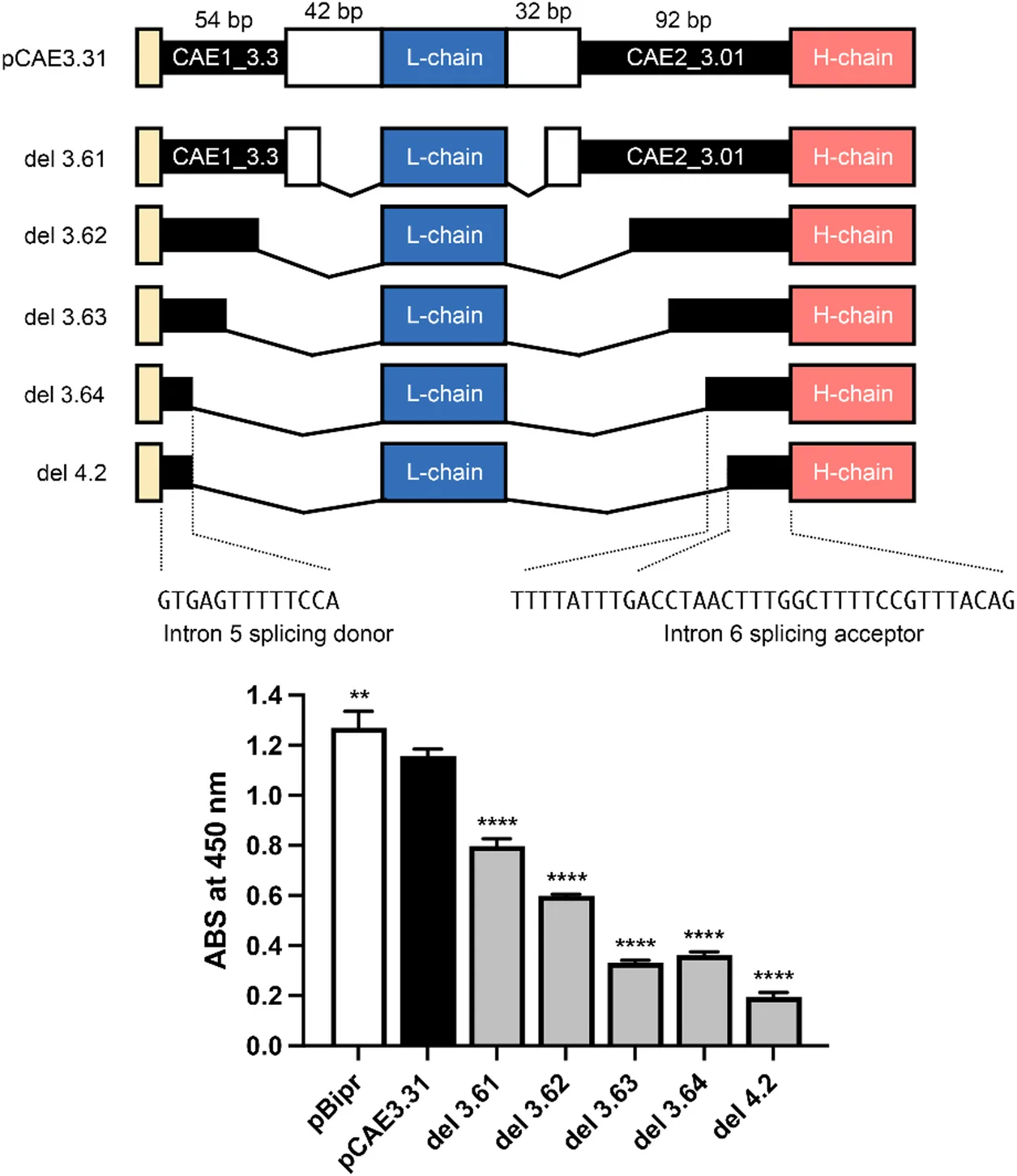
Further truncation of CAE region. Sequences around LC, including exon 7, were truncated from pCAE3.31. A schema of the truncation is shown in the figure. Detail sequence information was indicated in Supplementary Data 10. The del4.2 lacks the putative splicing branch site. Antibody concentration was measured by ELISA (mean ± SD of triplicates). Statistical significance was determined by Dunnett’s one-way ANOVA. The control group was pCAE3.31. ****P < 0.0001, **P < 0.01.

Given that CAE1_3.3 did not function as an intron and CAE2 had minimal effect on LC translation, we attempted to simplify the CAE structure by retaining only the CAE1 donor and CAE2 acceptor regions. Systematic deletions surrounding the LC region revealed that truncation within exon 7 reduced antibody expression (Fig. 5, del 3.61), suggesting that exon–intron boundaries near exon 7 are important for generating splicing variants that can drive antibody expression. Further deletions progressively decreased expression (Fig. 5, del 3.62–4.2).

**Fig. 6:**
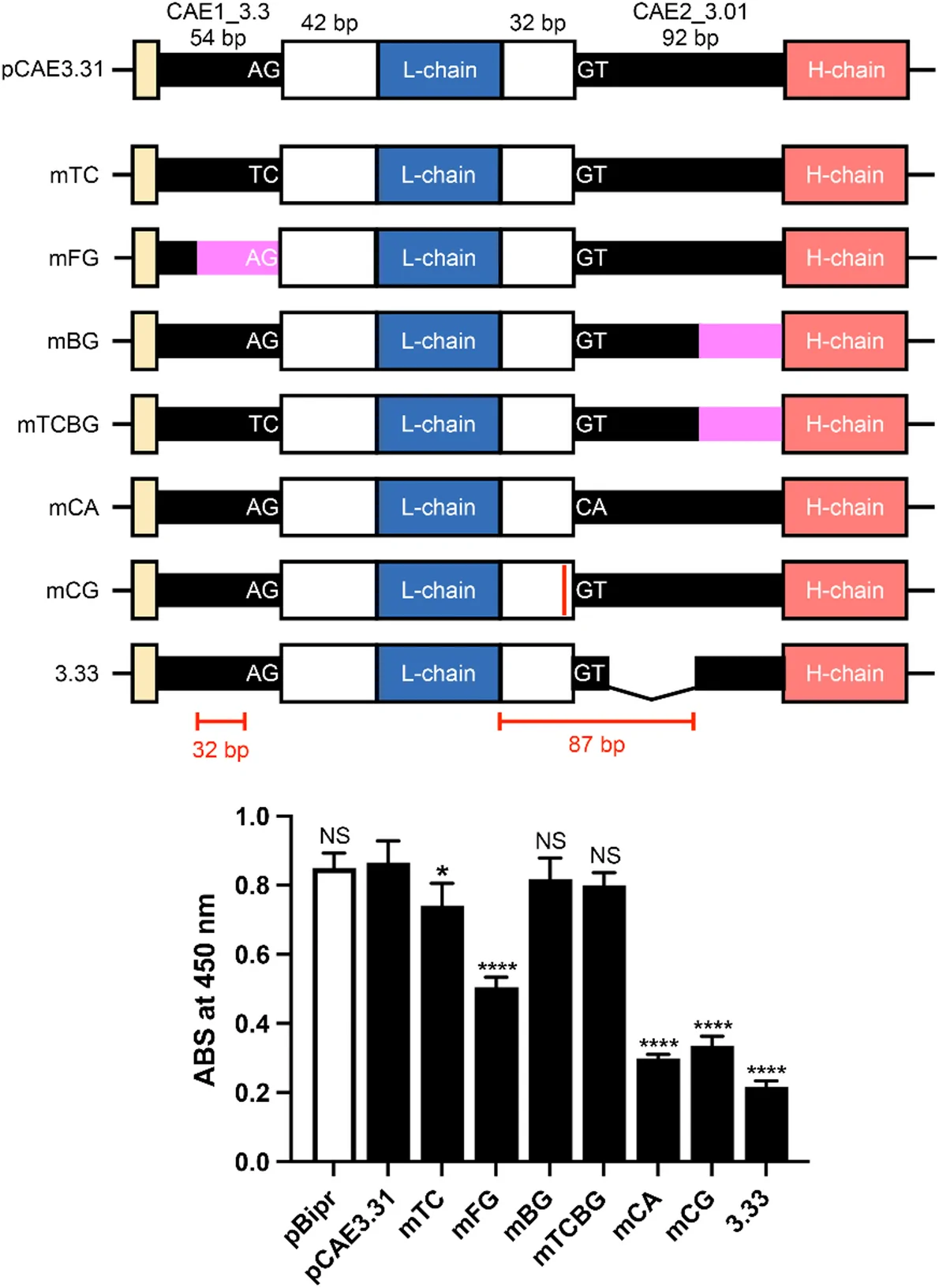
Regions responsible for alternative splicing between CAE1 and CAE2. The regions of splicing donor and accepter in CAE1 and CAE2 of pCAE3.31 were modified. A schema of replacement and truncation was shown in the figure. β-globin-derived splice acceptor is shown in magenta; red line in mCG indicates mutation in mCG. 3.33 corresponds to pCAE3.33 (CAE1_3.3+CAE2_3.03). Detail sequence information was indicated in Supplementary Data 10. Antibody concentration was measured by ELISA (mean ± SD of triplicates). Similar results were obtained in two independent experiments. The figure shows representative results. Statistical significance was determined by Dunnett’s one-way ANOVA. The control group was pCAE3.31. ****P < 0.0001, *P < 0.05.

To define sequence elements responsible for the splicing variants that generate LC and HC, several CAE mutants were tested (Fig. 6). Mutation of the CAE1 splice acceptor dinucleotide (“AG” → “TC”; mTC) had minimal effect, whereas replacement with a β-globin-derived acceptor (mFG) substantially reduced expression levels. These findings indicate that the native *HEG1* intron 5 acceptor sequence—specifically the 32 bp preceding the canonical “AG”—is essential for proper splicing. Conversely, the CAE2 acceptor could be replaced by the β-globin acceptor (mBG) without affecting expression levels. The mBG mutant that further replaced the CAE1 splice acceptor dinucleotides also unaffected expression (mTCBG). Mutations in the CAE2 donor (“GT” → “CA”; mCA) or in the exon 7 junction (“ACTCGGAG” → “CCAGGGAG”; mCG) decreased antibody production, as did shortening the CAE2 donor region (pCAE3.33). Collectively, these results demonstrate that CAE2 must be a functional intron containing both donor and acceptor motifs of sufficient length to achieve this alternative splicing. The 87 bp region encompassing exon 7 near CAE2 contributes to fine-tuning this balance. Based on these analyses, pCAE3.31 was selected as the optimal configuration for the CAE bicistronic expression system.

### Promoter compatibility and sequence optimization outside CAE

**Fig. 7:**
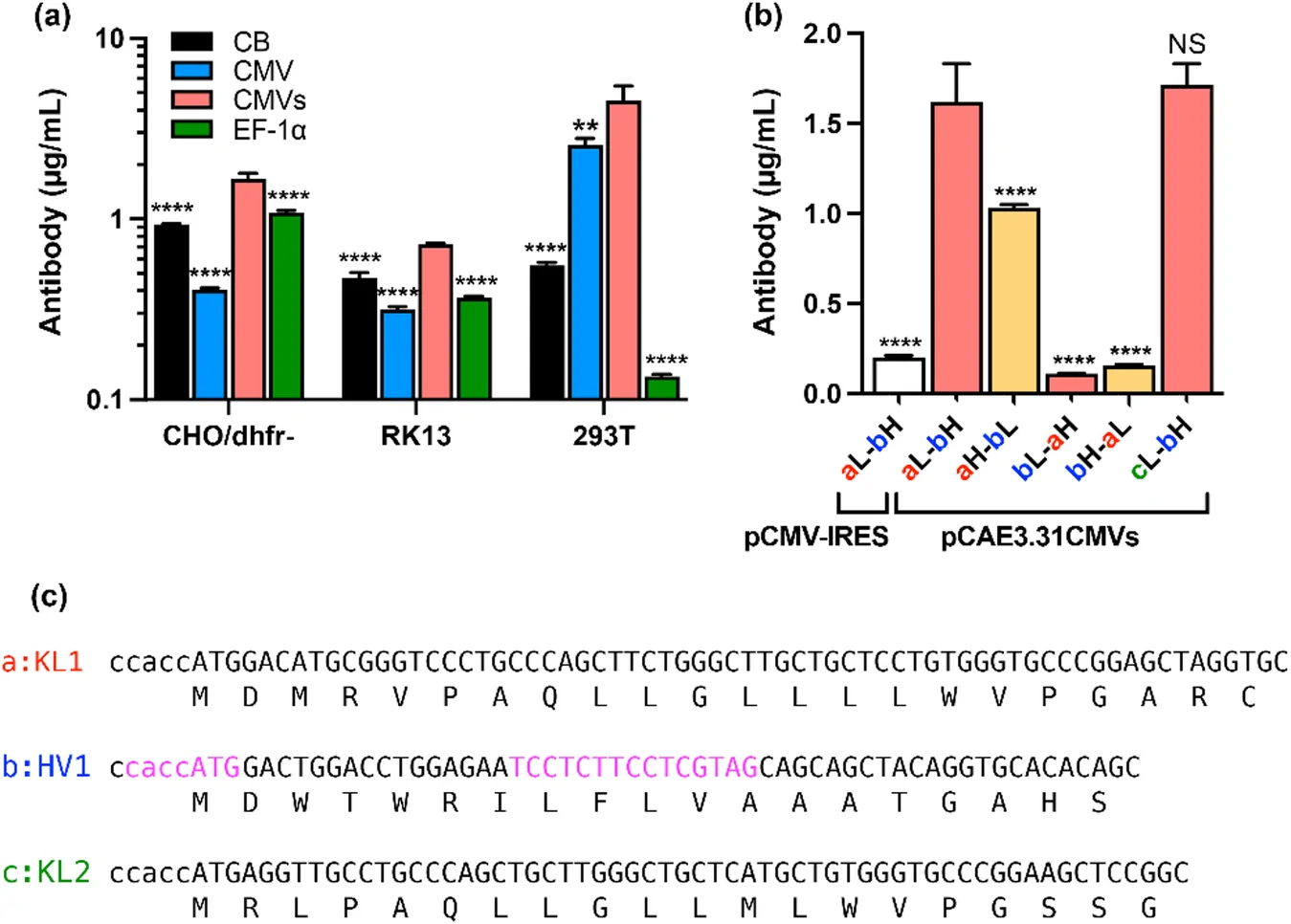
Effects of cell line, promoter, gene order, and signal peptides on antibody secretion. Active antibody concentration was measured by ELISA and calculated from purified standard antibody. Values represent the mean ± S.D. of triplicate determinations. (**a**) Comparison of promoters in three cell lines derived from different species. pCAE3.31 substituted with different strong promoters was transfected. CB, chicken β-actin promoter; CMV, CMV promoter; CMVs, CMV promoter with shortened GC-rich region following transcription start site; EF-1α, EF-1α promoter. In this figure, the y-axis shows a logarithmic scale. Statistical significance was determined by Dunnett’s one-way ANOVA. The control group was CMVs. ****P < 0.0001, **P < 0.01. (**b**) Comparison of bicistronic expression by IRES (pCMV-IRES) versus CAE (pCAE3.31CMVs). Replaced signal peptide sequences of LC or HC were indicated by lowercase letters. Statistical significance was determined by Dunnett’s one-way ANOVA. The control group was aL-bH in pCAE3.31CMVs. ****P < 0.0001. (**c**) Sequences of a: KL1, b: HV1, c: KL2; magenta in HV1 indicates putative acceptor and branch point.

To evaluate promoter compatibility, the chicken β-actin (CB) promoter in pCAE3.31 was replaced with alternative promoters (Supplementary Data 5) and transfected into several mammalian cell lines. All tested cell types produced functional antibodies with the original CB promoter (Fig. 7a). The cytomegalovirus (CMV) promoter reduced expression in CHO/dhfr- and RK13 cells but enhanced expression in 293T cells. A truncated CMV promoter lacking the GC-rich region downstream of the transcription start site (CMVs) markedly improved expression in all cell lines (Fig. 7a). Substitution with the human elongation factor-1α (EF-1α) promoter yielded comparable production to the CB promoter in CHO/dhfr- and RK13 cells but not in 293T cells.

When compared with a conventional IRES-based bicistronic construct (pCMV-IRES), the CAE-based plasmid pCAE3.31CMVs achieved an 8-fold higher antibody production (Fig. 7b, aL-bH in pCMV-IRES or pCAE3.31CMVs). Reversing the LC/HC order slightly reduced expression but maintained sufficient yield (aH-bL). These results suggest that CAE-mediated transcription provides a more suitable LC/HC ratio than IRES in mammalian cells.

Unexpected alternative splicing within the LC region was detected in earlier constructs (Supplementary Data 3). Because signal peptides are typically Leu/Phe-rich and encoded by C/T-rich codons, they tend to create cryptic splice acceptor sites. When such sequences were used upstream (b: HV1, we usually use as the signal sequences for downstream HC), antibody expression was almost completely lost (Fig. 7b, bL-aH, bH-aL). By contrast, replacing this sequence with another non-acceptor-containing signal peptide (c: KL2) restored expression (Fig. 7b, cL-bH). These results underscore the importance of avoiding potential intron acceptor motifs within upstream sequences in CAE system.

### Stable clone selection

**Fig. 8:**
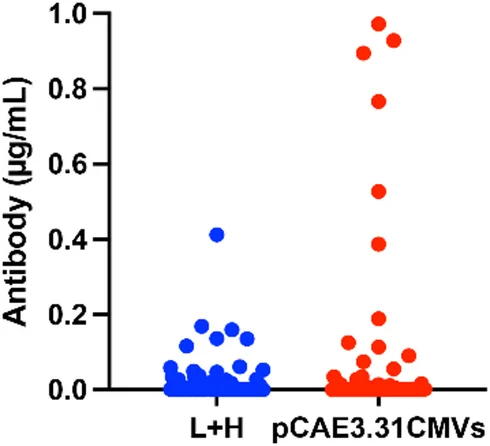
Stable expression of antibody using pCAE3.31CMVs. Sixty-nine drug-resistant clones were randomly selected, and antibody secretion was measured by ELISA. Most clones showed < 0.1 µg/mL, indicating loss of stable secretion. The difference of two group was not statistically significant on Brunner–Munzel test.

To assess the utility of CAE vectors for stable expression, CHO/dhfr– cells were transfected with pCAE3.31CMVs or with separate LC and HC plasmids. After selection in nucleotide-free medium containing selection drugs, sixty-nine drug-resistant clones from each condition were randomly selected from the grown colonies, expanded and then analyzed. The frequency of high-producing clones (~ 1 µg/mL, comparable to transient transfection) was higher for pCAE3.31CMVs. However, the difference was not statistically significant on Brunner–Munzel test because most clones had lost the ability to produce sufficient amounts of antibody (Fig. 8). These results suggest that CAE-based vectors may facilitate the isolation of high-producing stable clones.

### Antibody production in suspension culture

**Fig. 9:**
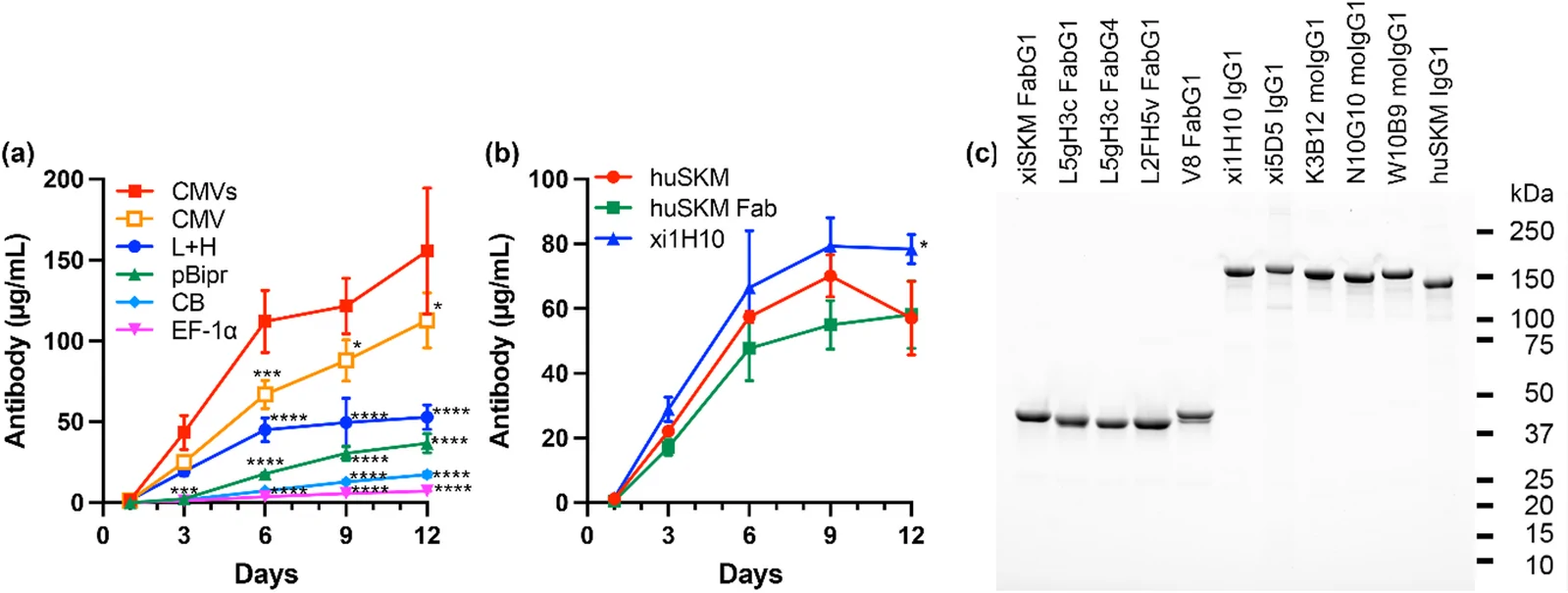
Antibody production in CHO suspension culture with pCAE3.31CMVs. Active antibody concentration was measured by ELISA and calculated from purified standard antibody. Values represent the mean ± S.D. of triplicate determinations. (**a**) Time-course of antibody production with different promoters (CMVs, CMV, CB, EF-1α) in pCAE3.31. L + H shows the co-transfection of two different plasmids for LC and HC with CMV promoters. Similar results were obtained in two independent experiments. The figure shows representative results. Statistical significance was determined by two-way ANOVA followed by Tukey’s multiple comparison test. ****P < 0.0001, ***P < 0.001 and *P < 0.05 compared to CMVs. (**b**) Time-course of Fab or different antibody production (1H10, anti-FGFR3). Statistical significance was determined by two-way ANOVA followed by Tukey’s multiple comparison test. *P < 0.05 compared to huSKM. (**c**) SDS-PAGE of affinity-purified Fab fragments or antibodies from ExpiCHO supernatants (3 µg/lane, non-reducing). Samples were resolved with Mini-PROTEAN TGX Stain-Free Gels 4–15% (BioRad Bio-Rad Laboratories Inc. Hercules, CA, USA) and the bands were detected by ChemiDoc Touch Imaging System (BioRad). Protein size marker: Precision Plus Protein Dual Color Standards (BioRad). Full-length gel image is presented in Supplementary Data 12.

Finally, we evaluated antibody production using pCAE3.31 constructs in the ExpiCHO transient expression system with high-density CHO-S cells. Antibody titers plateaued at similarly low levels with EF-1α- or CB-driven constructs (Fig. 9a), comparable to those achieved with the bi-promoter vector (pBipr). Co-transfection of separate LC and HC plasmids (L + H) resulted in an early cessation of production within 6 days. CAE constructs carrying CMV or CMVs promoters maintained continuous secretion, with CMVs showing faster initial expression but similar final titers (Fig. 9a).

Time-course analysis of another antibody clone (1H10) and a Fab fragment (huSKM Fab) confirmed comparable yields (Fig. 9b). Expression vectors containing a synthetic LC+CAE2 + VH cassette (~ 1.3 kb, Supplementary Data 6) enabled efficient production of diverse antibodies and fragments (Table 1). Purified products from 30 mL cultures reached 1–4 mg, encompassing human, mouse, and chimeric IgGs, Fabs, and bispecific formats. SDS-PAGE analysis confirmed correct LC: HC pairing (Fig. 9c). These results demonstrate that the CAE expression system provides a simple and versatile platform for efficient recombinant antibody and Fab production.

**Table 1:** Yield of antibodies or fragments purified from pCAE3.31CMVs-transfected ExpiCHO supernatants.

Form	Antibody name	Yield (mg)	Form	Antibody name	Yield (mg)
humanized IgG1	huSKM IgG1	4.4	humanized Fab	V8 FabG1	2.1
mouse-human chimera IgG1	xi1H10 IgG1	1.3		LV8 FabG1	1.8
	xi5D5 IgG1	2.9		MV8 FabG1	0.38
	xi1A2 IgG1	4.5		LsV8 FabG1	1.2
chiken-mouse chimera IgG	#110 moIgG1	1.0		sLV8 FabG1	1.4
	#110 moIgG2a	2.3		sMV8 FabG1	2.0
	#110 moIgG2b	2.7	mouse-human chimera Fab	xiSKM FabG1	2.2
mouse IgG1	KB12 moIgG1	4.1		xi1D2 FabG1	1.3
	NG10 moIgG1	4.0		xi5D11 FabG1	2.9
	W10B9 moIgG1	3.0		xiT6C7 FabG1	2.4
humanized Fab	L5gH3c FabG1	0.65		xi7D7 FabG1	1.4
	L5gH3c FabG4	0.42		xi5D5 FabG1	0.90
	L2FH5v FabG1	1.7	Fab-scFv bispecific fragments	hSKMOKTHis	2.9
	LI FabG1	0.50		LI FabOKTHis	3.4
	MI FabG1	1.0		LsI FabOKTHis	3.4
	LsI FabG1	0.52		xiSKMOKTHis*	2.8
	sLI FabG1	0.72			
	sMI FabG1	1.8			

Antibodies or fragments were affinity-purified using following the prepacked columns: humanized IgG1 and mouse-human chimera IgG1, HiTrap protein A HP 1 mL (Cytiva); mouse IgG1, HiTrap protein G HP 5 mL (Cytiva); humanized Fab, mouse-human chimera Fab, and Fab-scFv bispecific fragments, KANEKA KanCap G 1 mL (Kaneka Co.). Asterisk (xiSKMOKTHis) was purified by HisTrap excel 1 mL (Cytiva) with imidazole elution. Purification yields were calculated from absorbance at 280 nm and normalized to 30 mL culture supernatant. All samples were harvested 12 days post-transfection according to the High Titer Protocol of the ExpiCHO expression system.

## Discussion

In this study, we developed a novel bicistronic expression platform, termed the **cassette exon–associated (CAE) system**, and demonstrated its utility for recombinant antibody production. By exploiting an alternatively spliced cassette exon–derived mechanism from the HEG1 gene, the CAE system enables the generation of two transcripts encoding LC and HC from a single promoter. Compared with conventional bicistronic strategies such as IRES- or 2 A peptide–based systems, the CAE approach provides a more compact vector design while avoiding residual peptide sequences that can affect antibody quality. This architecture allows efficient expression of diverse antibody formats while maintaining a simple genetic configuration suitable for routine antibody engineering and screening applications. Together, these findings establish CAE as a versatile strategy for coordinated expression of antibody chains.

In the CAE system, native splice donor and acceptor sites derived from HEG1 are utilized, and the exon sequences adjacent to these splice sites are retained. This design is expected to preserve the ubiquitously balanced alternative splicing observed in HEG1^16^, thereby making the LC/HC production ratio less susceptible to the influence of inserted sequences. Consistent with this expectation, sufficient antibody expression was obtained for a variety of antibody clones and antibody fragments (Table 1). Structurally, the CAE system differs from the alternative splicing vector reported by Fallot et al.^15^, in which an intron is located upstream of the LC; instead, the CAE system contains an intron (CAE2) positioned between the LC and HC that is removed during mRNA maturation. The presence of a strong splice acceptor in this region may help suppress cryptic splicing arising from acceptor sites within the LC sequence, such as those reported by Fallot et al.^15^. Although cryptic splicing could theoretically occur between the splice donor upstream of the LC and splice acceptor motifs generated within the LC sequence, careful design of the signal peptide and framework 1 regions minimized its impact on antibody expression, as described in the next paragraph. In practice, differences between antibody clones had a greater impact on antibody yield than variations in splicing efficiency; antibody clones that exhibited low production in the CAE system also showed low yields when LC and HC were expressed from separate vectors (data not shown). Thus, a key advantage of the CAE system is that it can be applied to diverse antibody sequences without extensive optimization of the inserted genes.

One notable finding was that unintended splicing events occasionally occurred when the upstream coding sequence contained cryptic splice acceptor motifs. Such aberrant splicing reduced the amount of full-length LC transcripts and disrupted LC: HC balance, leading to diminished antibody yield. The signal peptide region, which is often rich in leucine and phenylalanine residues encoded by C- or T-rich codons, appeared particularly prone to generating these cryptic acceptor sites. This observation underscores the importance of codon composition during synthetic gene design. Ser/Pro/Leu-rich motifs in the VL framework 1 region (amino acid positions 7–15)^18^ may also form pseudo-acceptor motifs. Minimizing CT-rich codons and avoiding downstream “AG” dinucleotides—especially near residues 17–18 where Glu, Gln, Arg, or Lys are frequently encoded^18^—may mitigate this risk. Safe and risky sequences in signal peptide or VL framework 1 region are shown in Supplementary Data 7.

Predicting cryptic donor formation is more challenging. In pCAE3.0 and pCAE3.1, approximately 25–30% of transcripts exhibited alternative 5′ splicing at GT dinucleotides located around nucleotide positions 651 or 786 within the LC region. These donor sites shared a C/T-rich context followed by the “AAGGTG” (Lys–Val), suggesting that such sequences could serve as potential donor hotspots. These GT dinucleotides were the first and second nucleotides of the Val codon and therefore could not be replaced by any other nucleotide. Interestingly, these aberrant transcripts maintained correct CAE1 splicing but exhibited alternative 5′ junctions on the CAE2 side. In contrast, no aberrant transcripts were detected in pCAE3.31, which lacks the CAE1 splicing event. Although the exact mechanism remains unclear, the absence of CAE1 may reduce recognition efficiency at the CAE2 donor, thereby leading to incorrect mRNA processing. Use of pCAE3.31 would reduce this risk of 5′ alternative splicing.

Promoter expression performance varied depending on the host cell type^19^. In CHO-S cells, which support high transient protein expression, the CMVs promoter with a modified exon 1 provided the highest antibody yields and was relatively resistant to density-dependent suppression (Fig. 9a). Like CMVs, exon 1 modifications may improve the performance of CB or EF-1α, under dense culture conditions. In contrast, 293T cells transfected with EF-1α–driven constructs exhibited markedly reduced expression. Because the intron of EF-1α contains strong enhancer elements^20^, its replacement with the CAE cassette likely impaired promoter activity.

Although CAE-based vectors can yield a higher proportion of high-producing clones than conventional dual-vector systems, most clones lost the ability to produce detectable amounts of antibody. This loss may partly reflect the inherent properties of CHO/dhfr- cells. When drug-resistant stable clones were isolated by colony picking in CHO/dhfr- cells, the frequency of stable HEG1-expressing clones was low even when HEG1 was expressed using conventional expression vectors such as pcDNA (data not shown). On the other hand, this phenomenon may also reflect differences in LC: HC expression ratios, which are critical for efficient assembly of functional IgG molecules. Previous studies have shown that a slight HC predominance (~ 1.5-fold) enhances transient antibody expression^4,21,22^, whereas LC predominance (2–5-fold) favors long-term stable expression^4,23,24^. In the optimized CAE system, pCAE3.31CMVs, the LC: HC transcript ratio was approximately 3:1 in favor of LC expression, even under transient transfection conditions (Supplementary Data 4). Further tuning of splice-site strength or exon 7 sequences may enable optimization of the LC: HC ratio for improved stable clone generation. In the present study, stable expression was evaluated using circular plasmids and adherent cell cultures for ease of cloning and testing. Future studies aimed at improving stable expression should assess large-scale and long-term performance in suspension-adapted cell lines using linearized plasmids and analyze correlations between antibody yield and direct quantitative measurements of LC: HC transcript and protein ratios.

Beyond full-length IgGs, the CAE system proved advantageous for Fab fragment expression. Accurate affinity measurements require monomeric Fab fragments to avoid avidity artifacts from IgG dimerization. However, traditional bicistronic vectors often yield low expression levels or include residual peptide tags that may alter binding properties. The CAE system overcomes these issues: simple insertion of a ~ 1.3 kb synthetic LC+CAE2 + VH cassette enables rapid generation of diverse Fab constructs. Even small-scale (10 mL) transient expression yielded sufficient purified Fab quantities for affinity analysis. This compact and flexible system therefore provides a practical platform for high-throughput antibody and fragment screening.

In summary, the CAE expression system represents a robust, size-efficient, and promoter-compatible bicistronic platform that enables sufficient expression of LC and HC without additional peptide sequences. Its simplicity, adaptability, and compatibility with major mammalian promoters make it a promising tool for both rapid antibody screening and stable production of therapeutic mAbs.

## Materials and methods

### Plasmid construction

HEG1 introns, antibody genes, and promoter sequences are listed in Supplementary Data 1, 2, or 5. cDNAs were synthesized or amplified by PCR using PrimeSTAR HS DNA polymerase (Takara Bio Inc.). IRES and dihydrofolate reductase gene were amplified from pOptiVec (Thermo Fisher Scientific Inc.), neomycin resistance gene and CAG promoter from pCXN2, and ampicillin resistance gene and origin of replication from pUC19. Plasmids were assembled into pCAE constructs via In-Fusion cloning (Takara Bio). Point and deletion mutants were generated by site-directed mutagenesis. Plasmid maps of original vectors are shown in Supplementary Data 5.

### Cell culture and transfection

CHO/dhfr- cells (ECACC, EC94060607) were maintained in MEM α with nucleotides and 5% fetal bovine serum. RK13 or 293T cells (RIKEN Cell Bank, RCB0183 or RCB2202) were cultured in RPMI-1640 or DMEM with 5% fetal bovine serum. Plasmids were transfected using Lipofectamine LTX reagent with Plus reagent (Thermo Fisher Scientific) (CHO/dhfr-) or Lipofectamine 3000 (Thermo Fisher Scientific) (RK13, 293T) at 0.55 µg per 0.5 mL in 24-well plates. ExpiCHO-S cells were cultured with the ExpiCHO Expression System (Thermo Fisher Scientific).

### ELISA

Humanized SKM9-2 IgG1 was used as a model antibody^25^. Culture supernatants were assayed by ELISA. Recombinant 762EGF ligand, including SKM9-2 epitope, was purified from culture supernatant of stably transfected 293 H cells via Ni-affinity and anion exchange chromatography (Supplementary Data 8). Plates were coated with 1 µg/mL 762EGF, blocked with 20% Blocking One (Nacalai tesque, Inc.), incubated with diluted samples for 3 h, washed, and incubated with HRP-conjugated goat anti-human Fcγ or F(ab’)_2_ (Jackson ImmunoResearch Laboratories, Inc.). Detection was performed using 1-Step Ultra TMB (Thermo Fisher Scientific) and stopped with 2 M H_2_SO_4_. Purified SKM9-2 was used as a standard. The measurement range of the ELISA is shown as a representative standard curve in Supplementary Data 9. Statistical analyses were performed using GraphPad Prism 10 software. *P*-values were calculated as indicated in the figure legends.

### RT-PCR

Total RNA was extracted with TRIzol Reagent (Invitrogen) from CHO/dhfr- transfected after 2 d. After treating with RQ1 RNase-free DNase (Promega K.K.) and ice-cooling, the RNA (25 ng per 20 µL of reaction volume) was reverse transcribed and amplified as cDNA with the OneStep RT-PCR kit (Qiagen GmbH) with 0.6 µM primers (forward: 5’-GCTCTGACTGACCGCGTTACTCCCA-3’, reverse: 5’-TCCCAGGAGTTCTGGTGCAGGACAA-3’). Reverse transcription: 45 °C, 30 min. PCR: 95 °C, 15 min; 32 cycles of 94 °C, 10 s; 59 °C, 30 s; 68 °C, 3 min. Products were resolved on 1.3% agarose gels, cloned using Mighty TA-Cloning (Takara Bio), and sequenced (> 11 clones per band). 1 kbp DNA Ladder One (Nacalai Tesque) was used as marker.

### Stable clone selection

To isolate stable expression clones of humanized SKM9-2, pCAE3.31CMVs or co-transfection with pcDNA3.1 (for LC) and pOptiVec (for HC) (Thermo Fisher Scientific Inc.) was used. Plasmids (0.55 µg) were transfected into CHO/dhfr- in 24-well plates with Lipofectamine LTX reagent with Plus reagent (Thermo Fisher Scientific Inc.). After 1 day of culture, the cells were re-seeded onto 10-cm dishes and the stable expression clones were selected with cultivation in Minimum Essential Medium α without nucleotides, containing 5% fetal bovine serum and 750 µg/mL of Geneticin (Thermo Fisher Scientific). The colonies of stable clone were isolated, re-cultured in 96-well plates until they reached confluency, and then the culture supernatants (150 µL) were collected after 2 d incubation following a medium change. The concentration of active antibody was measured by ELISA.

## Supplementary Information

Below is the link to the electronic supplementary material.

Supplementary Material 1

## Data Availability

All data generated or analyzed during this study are included in this published article and its Supplementary Information files.
