# Supplementary material for: A novel bicistronic vector inducing balanced alternative splicing for antibody production
Source: Sci Rep. 2026 Jun 11;16:26632. doi: 10.1038/s41598-026-57819-1 (PMC13503707; doi:10.1038/s41598-026-57819-1)

Supplementary Information

A novel bicistronic vector inducing balanced alternative splicing for antibody production.

Shoutaro Tsuji[1]*

[1]Department of Medical Technology & Clinical Engineering, Gunma University of Health and Welfare, Gunma, 371-0823 , Japan.

*Address correspondence to: Shoutaro Tsuji, Ph.D.: Gunma University of Health and Welfare, 191-1 Kawamagari-machi, Maebashi, Gunma 371-0823, Japan; e-mail: tsuji-s@shoken-gakuen.ac.jp.

Supplementary Data 1

Sequences from exon 5 to exon 7 of human HEG1, including intron 5 and intron 6. These sequences are located at 36041–43375 in GenBank accession no. NC_000003 (region 124965710–125055997). Uppercase sequence regions were used for CAE expression. The intron 11 of HEG1 is located at 53742-53985. The uppercase sequences were inserted in CAE2_3.00 as a spacer. The intron 14 of HEG1 is located at 65055-65173. The uppercase sequences were inserted in CAE1_3.0 as a spacer.

HEG1 exon 5 (336 bp)
cctttggagaacatcagcttgccagcagctctgaggtgcaaaatggaagtcccatgtctcagactgagactgtgtctaggtcagtcg
cacccatgagaggtggagagatcactgcacactggctcttgaccaacagcacaacatctgcagatgtgacaggaagctctgcttca
tatcctgaaggtgtgaatgcttcagtgttgacccagttctcagactctactgtacagtctggaggaagtcacacagcattgggagata
ggagttattcagagtcttcatctacatcttcctcggaaagcttgaattcatcagcaccacgtggagaacgttCGA

HEG1 intron 5 (4249 bp)
GTGAGTTTTTCCATTTCATGCAACTGTGCCCCATAGAGGAGAGAGGGactcatgatgctt
atgaaatctgtggcgtattaaaaccacgcatgagggcctagcgtggtggctcacgcctgtaatcccagcactttgggaggccgagg
tgggtggatcatctgaggtgaggggttcaagacctgactgacctacatggtgaaaccctgtctctactaacaatacaaaaaattagcc
gggtgtgatggcaggcacctgtaatcccagctacttgggaggctgaggcaggagaatcgtttgaacccaggaggtggaggttgca
gttagctgagatcgcaccactgcactccagcctgggcgacaaaagcgaaagtgtctcaaaaaaaaaaaaaaaaaaaaagcatgc
atgaatgaaataactcctcaggcccctccaccttccttccttatttcccaggcccttcttctagctggttgatacagatacacctatatatc
ccccagtttttctggtacatctctgagaagctgttcttgccacttcctcttcccacacacagctcagggacttctctgtccacatcctctc
attccctgtagccaacccataactccatacctgcctcagtcacagagcttctggaaggtgaaaatatcaccttctcgttagtagttgtcg
gggcattcatccaaataaattctctgtttttaaaaaccagctttattgaggtatatttcatattccatagtatttgcccatttaaagcacaca
attcagtgatttttagtgtagtcacaaagctgtgcagcatcaccactgtctaattccagagcatttccatcaccccacaaagaaactcca
aactcatcagcagtcaactcccattctcccctcctcccagcccctggcaatcactcctgtgctttctgtctctgtagatttgtccattttag
acatttcctataaatgaaatcatacaatatgtggtcctttgtgtctttcccttagtgtcatgttttcaaggttcatccacgttgtatcacaactt
cgttccttttcgtggcttaataatatgccgtagcagggatataccacactttgtttatcaattgtcatcaattgatggatgtttgggttgttcc
caccttttggctattatgaataatgctgtggtgaacattcaggtacaagtgtatatgtggtcgtaaattttcaatttatttttttttttttgagatg
gagtctctctctgttgcccaggctagagtgcagtggcaccatcccggctcactgcaatctccgacttccgggttcacgccattctcct
gcctcagcctcccgagtagctgggactacaggtgcccaccaccacgcctggctaattttttttttttttttatattttcagtagagacaggg
tttcaccatgttagccaggatggtcccgatctcctgacctcatgatccgcctgcttcagcctcccaaagtgctgggattgcaggcatg
agccaccatgcctggccataaattttcaattatttaggaatggccaggttatgtggctgtggctgctctatttttaaccttctgaggaact
gccgaactcttttccaaagtggctgcatcattttatattcccaccagctgtgtacaagtgttctgatgttgctacatcttcaccaacacttgt
aattatccatcttttctgattatagccatctgagtgggggtggaggcatctcattgtggttttgacttgtattttccagttgactaataatgct
gagcatcttttcatgtgcttattggccacttgtatatcttctttgaatatgtctagtccaatcttttgactattaaaattgggttatttgacttttta
ttattaaattgtaagagttctttatggccggctgtggtggcttacgcctgtaatcccagcactttgggaggtcaaggcgggtgaatcact
tgaggtcaggagttcaagaccagcccggccaacatggcaaaaccctgtctctactaaaaatacaaaaattagccagacatagtggc
acgtgcctgtaatctcagctacttgggaggctgaggcaggagaatcacttgaacctgtgaggtggaggttgcagtgaactgagatc
atgccactgcactctagcctgggtgacaaacagaccctgtctcaaaaaaaaaaaaaaaagttctttgcatattttggatacagtcctttat
caaatatataatttgcagacatttctcctattctgtgggttgtcttttctctttcttgatggaatcatttgtagcacaaaagtttttaaatttgaa
gaagtccaatttgtcaatttgagaaaatttttcttttgatgtttctttttaataatgcttagggataatttttaaaacaattctcacagtctgttat
caatgtaaagtatattctgactaaagcaaaaatgtattagctgatcatttaagctttgaataggtttaaattcttacttctccatcactagtta

tagcttcatttcccccatataagaatcctaattgatgaggaagattagcatttattagtactcacgtgatgtggaaaagcatatgcttatca
tgaaagaataccacacacacaagcgggggatatttatgctcttttatgtttgtgcctggaaaacacattatctatgtcctaagcaaaagg
aatcatttttcaccctcactactgtgggggtcaggacttctaccttctcaaaccaggatcttgagtttcatagcctttctcttttacatctctt
cagtacccgcttctctgcaattaatgctcagttggttcatgaggaacatatgatttcagtgtgatcagatcttggagttcatttataaagag
ctagatagaaagtagccacattcctgaaaccatctcactgtgggttaacacaatactttatctttttccctcagagtcacccaaagctgg
cggggttctgcactccggccttttagagttcttggcccgctcccttttctgcattcagagtctccatttcatgtctgactctctctctactttg
ttaagcctgtaaccctgatgtggtcattggcctaaatttcatttaacagagagtttaggatctccccatccaagaccttttctctttgccca
gagaaatcgtacttagatcctgctcttgcgcctctcctggttggctctccacaggtcttgagcccttcagactggcttgagtcgcatctc
tgtctggcttctggctttctcagtgatgctcgtggcccgtgctcattcctgtctctgtcaattcaaacctatcccaaaatgaatttagccaa
cactcagctttacactgttttccatcaaagcatggccatggcgtactgacagccagtctgggctgggaatgtgaataagaaccagaa
aagagcctgtttttgtttttgggtttttttttctttttgtacagccagattaggaaggatttaggccaaggagaattggacctcattttcatc
atttctgaagagaagggtttatctgataatctaacaatctaaaattagttctaagaccctttaggtttgtagcataaacacatacccaaaa
gagaatgaaacagacatttattcatgtgtcgtctgccctcttccatccccatcaatagaataaaacacatgcattttaaaaaataaaaga
agacaaaaaaacaagcccacctttcacccccacctcaggctgtttctttgccattgtactaatgccgtgtaatgactggccttgtggc
cattcatgtattcctggtccttccgggaggaagagccttccaaggaggactatggcctcccttctgggccttgtgcaaatgtctgaag
ccccagtaggattattagtctttcaccaactgtttacctcctcaccaccactaccaatttctctgacataaactctatttattatgtatcactg
agcagaaatctagtatcgatcctatttgctaaagtaatataccctcctataaatccagggtgcctgaacagttgtgctgatccacagttc
ctcttgaggtttcaatcactgagccatgtttgcaagggcaacagacatctcagataaagaccctaaaagtacaggggagcgtcttgg
ctcccacagcccaggaagtgaggcttggggcacgatcctgtcgcTCCAGCCCTGCATTTTTCAGCCATCCT
CTTTCACTCTACTTTTAG

HEG1 exon 6 (300 bp)
CCTTGGAAGACAGCCGAGAGCCAGGCCAAGCACTAGGTGACAGttccgccaatgcagag
gacaggacttctggggtgccctctctcggcacccacaccttggctactgtcactggaaacggggaacgcacactgcggtctgtcac
cctcaccaacaccagcatgagcacgacttctggggaagcaggcagccctgcagcggccatgcaccaagaaacagagggtgcct
ctctgcacgtaaacgtgacggacgacatgggcctggtcTCACGGTCACTGGCCGCCTCCAGTGCACTC
GGAG

HEG1 intron 6 (722 bp)
GTGGGGTGTCTTCCACAGTCTCTTTTAACACATCCCTTCAGCTcatctattaatttactcaccgt
cctgggatgattcagttagctgtcaagccagaaagtgccttacaagctctttatggagagaactttctagattctcccacctcaggaag
gtgaataccccctccccagttcccacccccagccctccacacacactcacctaaggaatttgggatagtgtttgctgggagacttaaaa
taagagaccaaccacttgtggctaaggtcaatctcctgggaaagaaatatctcattctatagaatggaaagttgggaagattttcctttt
tcatttaggcataacagttattctatttggggtcttaagagcttttcagataccagtttgcttttatcgaaaaaggcatgtggaggaaatttg
caactcctctccatttttagacccttagtccccacttctcttttgttaccatttccactgccgctaccactgcttctctcaggaggtcagtct
gctcagaggagacactgaccactcctgcctgcccacaatgtctttattcagagtgctcacccttgaaaaccacctctccaaagcaagt
gtttcacccacttgaattcattgagtttcatgacactgaaagtctgtttgatagtgcatatttcagAGTTTTGTTGTTCTTTT
ATTTGACCTAACTTTGGCTTTTCCGTTTACAG

HEG1 exon 7 (1368 bp)
TCGCTgggattagctacggtcaagtgcgtggcacagctattgaacaaaggacttccagcgaccacacagaccacacctacctg
tcatctactttcaccaaaggagaacgggcgttactgtccattacagataacagttcatcctcagacattgtggagagctcaacttcttat
attaaaatctcaaactcttcacattcagagtattcctcctttttttcatgctcagactgagagaagtaacatctcatcctatgacggggaata
tgctcagccttctactgagtcgccagttctgcatacatccaaccttccgtcctacacacccaccattaatatgccgaacacttcggttgtt

ctggacactgatgctgagtttgttagtgactcctcctcctcctcttcctcctcctcctcttcttcttcttcagggcctcctttgcctctgccct
ctgtgtcacaatcccaccatttattttcatcaattttaccatcaaccagggcctctgtgcatctactaaagtctacctctgatgcatccaca
ccatggtcttcctcaccatcacctttaccagtatccttaacgacatctacatctgccccactttctgtctcacaaacaaccttgccacagt
catcttctacccctgtcctgcccagggcaagggagactcctgtgacttcatttcagacatcaacaatgacatcattcatgacaatgctc
catagtagtcaaactgcagaccttaagagccagagcaccccacaccaagagaaagtcattacagaatcaaagtcaccaagcctgg
tgtctctgcccacagagtccaccaaagctgtaacaacaaactctcctttgcctccatccttaacagagtcctccacagagcaaacccctt
ccagccacaagcaccaacttagcacaaatgtctccaactttcacaactaccattctgaagacctctcagcctcttatgaccactcctgg
caccctgtcaagcacagcatctctggtcactggccctatagccgtacagactacagctggaaaacagctctcgctgacccatcctga
aatactagttcctcaaatctcaacagaaggtggcatcagcacagaaaggaaccgagtgattgtggatgctaccactggattgatccc
tttgaccagtgtacccacatcagcaaaagaaatgaccacaaagcttggcgttacagcagagtacagcccagcttcacgttccctcgg
aacatctccttctccccaaaccacagttgtttccacggctgaagacttggctcccaaatctgccacctttgctgttcagagcagcacac
agtcaccaacaacagtgtcctcttcagcctcag

HEG1 intron 11 (244 bp)
gtaagtaaCAGAATTATATTTGCTTGTGAACTAGTCTGTACAAAGGGGAGGCTGTTCT
AACATGGGTGATAGCAACAAAACCGGGCTGTTTTCACCCAGCCATGGGGCAATC
CAGGGCCAAGGTCACTGACCTTCATGCTCTCCCATCCCGTCACTGGTGAATGGC
GATGACATTCATTTCCAAAAGCCTTGGGACCCACGTGTCATTTCAGggctgttaaaaacc
cacaaactttgcctatag

HEG1 intron 14 (119 bp)
gtaggCTCCATGTTGATTTAGTCTAATTTTGTTACAAATCTTATTTAGATAAATTGCCT
CACTTATTTCTTTTGAGAttggaaactaattcttgcccttttttctttcttggcttgcag

Supplementary Data 2

pCAE3.0 plasmid map

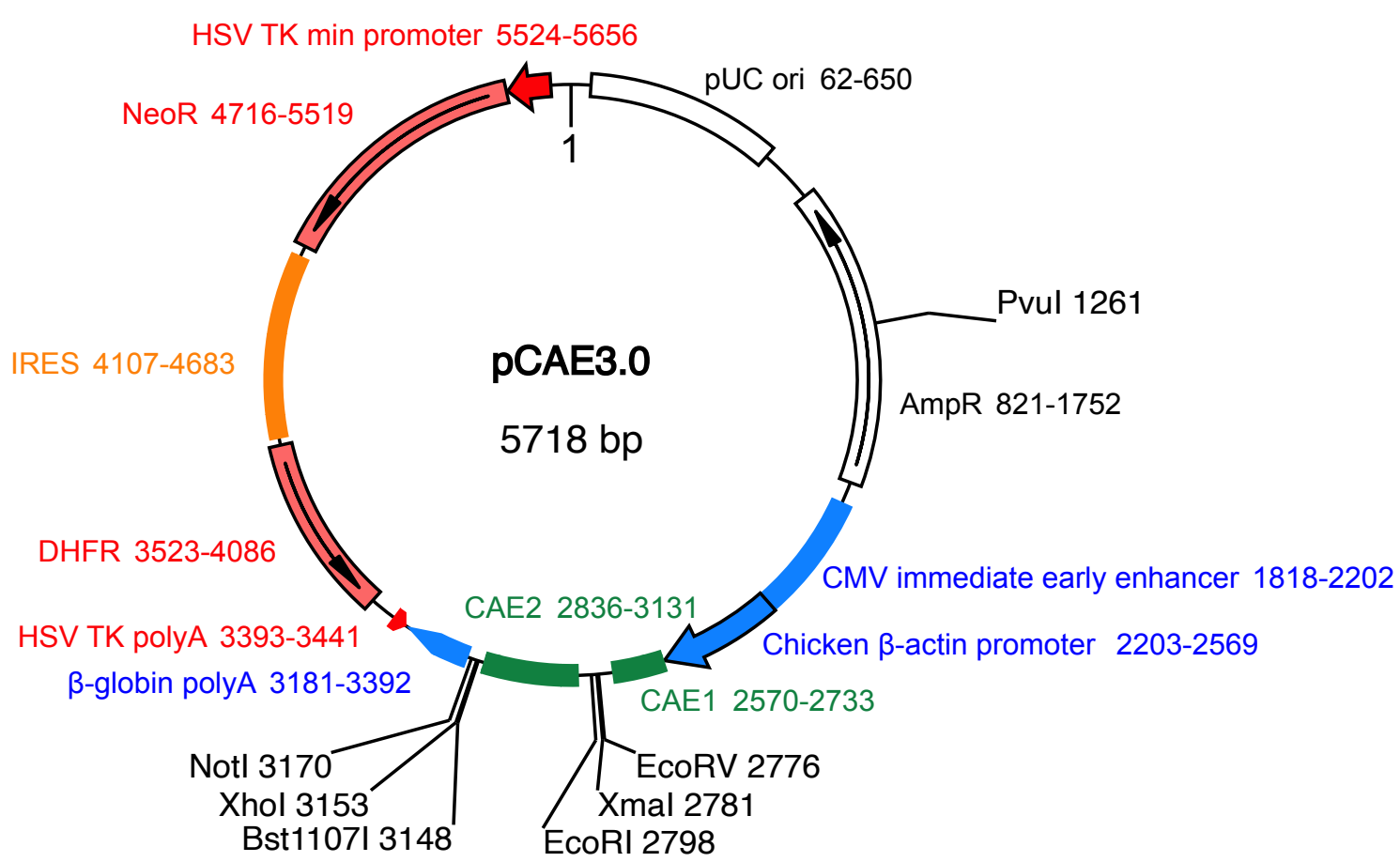

pCAE3.0 sequences

ACATGTGAGCAAAAGGCCAGCAAAAGGCCAGGAACCGTAAAAAGGCCGCGTT
GCTGGCGTTTTTCCATAGGCTCCGCCCCCCTGACGAGCATCACAAAAATCGACG
CTCAAGTCAGAGGTGGCGAAACCCGACAGGACTATAAAGATACCAGGCGTTTCC
CCCTGGAAGCTCCCTCGTGCGCTCTCCTGTTCCGACCCTGCCGCTTACCGGATAC
CTGTCCGCCTTTCTCCCTTCGGGAAGCGTGGCGCTTTCTCATAGCTCACGCTGTA
GGTATCTCAGTTCGGTGTAGGTCGTTCGCTCCAAGCTGGGCTGTGTGCACGAAC
CCCCCGTTCAGCCCGACCGCTGCGCCTTATCCGGTAACTATCGTCTTGAGTCCAA
CCCGGTAAGACACGACTTATCGCCACTGGCAGCAGCCACTGGTAACAGGATTAG
CAGAGCGAGGTATGTAGGCGGTGCTACAGAGTTCTTGAAGTGGTGGCCTAACTA
CGGCTACACTAGAAGAACAGTATTTGGTATCTGCGCTCTGCTGAAGCCAGTTACC
TTCGGAAAAAGAGTTGGTAGCTCTTGATCCGGCAAACAAACCACCGCTGGTAGC
GGTGGTTTTTTTGTTTGCAAGCAGCAGATTACGCGCAGAAAAAAAGGATCTCAA
GAAGATCCTTTGATCTTTTCTACGGGGTCTGACGCTCAGTGGAACGAAAACTCA
CGTTAAGGGATTTTGGTCATGAGATTATCAAAAAGGATCTTCACCTAGATCCTTTT
AAATTAAAAATGAAGTTTTAAATCAATCTAAAGTATATATGAGTAAACTTGGTCTG
ACAGTTACCAATGCTTAATCAGTGAGGCACCTATCTCAGCGATCTGTCTATTTCGT
TCATCCATAGTTGCCTGACTCCCCGTCGTGTAGATAACTACGATACGGGAGGGCT
TACCATCTGGCCCCAGTGCTGCAATGATACCGCGAGACCCACGCTCACCGGCTC
CAGATTTATCAGCAATAAACCAGCCAGCCGGAAGGGCCGAGCGCAGAAGTGGT
CCTGCAACTTTATCCGCCTCCATCCAGTCTATTAATTGTTGCCGGGAAGCTAGAG
TAAGTAGTTCGCCAGTTAATAGTTTGCGCAACGTTGTTGCCATTGCTACAGGCAT
CGTGGTGTCACGCTCGTCGTTTGGTATGGCTTCATTCAGCTCCGGTTCCCAACGA
TCAAGGCGAGTTACATGATCCCCCATGTTGTGCAAAAAAGCGGTTAGCTCCTTC
GGTCCTCCGATCGTTGTCAGAAGTAAGTTGGCCGCAGTGTTATCACTCATGGTTA

TGGCAGCACTGCATAATTCTCTTACTGTCATGCCATCCGTAAGATGCTTTTCTGTG
ACTGGTGAGTACTCAACCAAGTCATTCTGAGAATAGTGTATGCGGCGACCGAGT
TGCTCTTGCCCGGCGTCAATACGGGATAATACCGCGCCACATAGCAGAACTTTAA
AAGTGCTCATCATTGGAAAACGTTCTTCGGGGCGAAAACTCTCAAGGATCTTAC
CGCTGTTGAGATCCAGTTCGATGTAACCCACTCGTGCACCCAACTGATCTTCAGC
ATCTTTTACTTTCACCAGCGTTTCTGGGTGAGCAAAAACAGGAAGGCAAAATGC
CGCAAAAAAGGGAATAAGGGCGACACGGAAATGTTGAATACTCATACTCTTCCT
TTTTCAATATTATTGAAGCATTTATCAGGGTTATTGTCTCATGAGCGGATACATATT
TGAATGTATTTAGAAAAATAAACAAATAGGGGTTCCGCGCACATTTCCCCGAAA
AGTGCCACCTGACGTCGTCGACATTGATTATTGACTAGTTATTAATAGTAATCAAT
TACGGGGTCATTAGTTCATAGCCCATATATGGAGTTCCGCGTTACATAACTTACGG
TAAATGGCCCGCCTGGCTGACCGCCCAACGACCCCCGCCCATTGACGTCAATAA
TGACGTATGTTCCCATAGTAACGCCAATAGGGACTTTCCATTGACGTCAATGGGT
GGAGTATTTACGGTAAACTGCCCACTTGGCAGTACATCAAGTGTATCATATGCCA
AGTACGCCCCCTATTGACGTCAATGACGGTAAATGGCCCGCCTGGCATTATGCCC
AGTACATGACCTTATGGGACTTTCCTACTTGGCAGTACATCTACGTATTAGTCATC
GCTATTACCATGGTCGAGGTGAGCCCCACGTTCTGCTTCACTCTCCCCATCTCCC
CCCCCTCCCCACCCCCAATTTTGTATTTATTTATTTTTTAATTATTTTGTGCAGCGA
TGGGGGCGGGGGGGGGGGGGCGCGCGCCAGGCGGGGCGGGGCGGGGCGAGG
GGCGGGGCGGGGCGAGGCGGAGAGGTGCGGCGGCAGCCAATCAGAGCGGCGC
GCTCCGAAAGTTTCCTTTTATGGCGAGGCGGCGGCGGCGGCGGCCCTATAAAAA
GCGAAGCGCGCGGCGGGCGGGAGTCGCTGCGCGCTGCCTTCGCCCCGTGCCCC
GCTCCGCCGCCGCCTCGCGCCGCCCGCCCCGGCTCTGACTGACCGCGTTACTCC
CACGAGTGAGTTTTTCCATTTCATGCAACTGTGCCCCATAGAGGAGAGAGGGCT
CCATGTTGATTTAGTCTAATTTTGTTACAAATCTTATTTAGATAAATTGCCTCACTT
ATTTCTTTTGAGATCCAGCCCTGCATTTTTCAGCCATCCTCTTTCACTCTACTTTT
AGCCTTGGAAGACAGCCGAGAGCCAGGCCAAGCACTAGGTGACAGATATCCCG
GGccaccatgtaaGAATTCACGGTCACTGGCCGCCTCCAGTGCACTCGGAGGTGGGGT
GTCTTCCACAGTCTCTTTTAACACATCCCTTCAGCTCAGAATTATATTTGCTTGTG
AACTAGTCTGTACAAAGGGGAGGCTGTTCTAACATGGGTGATAGCAACAAAACC
GGGCTGTTTTCACCCAGCCATGGGGCAATCCAGGGCCAAGGTCACTGACCTTCA
TGCTCTCCCATCCCGTCACTGGTGAATGGCGATGACATTCATTTCCAAAAGCCTT
GGGACCCACGTGTCATTTCAGAGTTTTGTTGTTCTTTTATTTGACCTAACTTTGGC
TTTTCCGTTTACAGTCGCTCGTGAGGAATTGTATACTCGAGccaccatgtaaGCGGCCG
CTGAGCTCAATTCACTCCTCAGGTGCAGGCTGCCTATCAGAAGGTGGTGGCTGG
TGTGGCCAATGCCCTGGCTCACAAATACCACTGAGATCTTTTTCCCTCTGCCAAA
AATTATGGGGACATCATGAAGCCCCTTGAGCATCTGACTTCTGGCTAATAAAGGA
AATTTATTTTCATTGCAATAGTGTGTTGGAATTTTTTGTGTCTCTCATTTATGAACA
AACGACCCAACACCCGTGCGTTTTATTCTGTCTTTTTATTGCCGTCATAGCGCGG
GTTCCTTCCGGTATTGTCTCCTTCCGTGTTTCAGTTAGCCTCCCCCGTTTAAACTC
ATTACTAACCGGTTTTAGTCTTTCTTCTCGTAGACTTCAAACTTATACTTGATGCC
TTTTTCCTCCTGGACCTCAGAGAGGACGCCTGGGTATTCTGGGAGAAGTTTATAT
TTCCCCAAATCAATTTCTGGGAAAAACGTGTCACTTTCAAATTCCTGCATGATCC
TTGTCACAAAGAGTCTGAGGTGGCCTGGTTGATTCATGGCTTCCTGGTAAACAG

AACTGCCTCCGACTATCCAAACCATGTCTACTTTACTTGCCAATTCCGGTTGTTC
AATAAGTCTTAAGGCATCATCCAAACTTTTGGCAAGAAAATGAGCTCCTCGTGG
TGGTTCTTTGAGTTCTCTACTGAGAACTATATTAATTCTGTCCTTTAAAGGTCGAT
TCTTCTCAGGAATGGAGAACCAGGTTTTCCTACCCATAATCACCAGATTCTGTTT
ACCTTCCACTGAAGAGGTTGTGGTCATTCTTTGGAAGTACTTGAACTCGTTCCTG
AGCGGAGGCCAGGGTAGGTCTCCGTTCTTGCCAATCCCCATATTTTGGGACACG
GCGACGATGCAGTTCAATGGTCGAACCATGGTGGCAGATCTTGTGGCCATATTAT
CATCGTGTTTTTCAAAGGAAAACCACGTCCCCGTGGTTCGGGGGGCCTAGACGT
TTTTTTAACCTCGACTAAACACATGTAAAGCATGTGTACCGAGGCCCCAGATCAG
ATCCCATACAATGGGGTACCTTCTGGGCATCCTTCAGCCCCTTGTTGAATACGCTT
GAGGAGAGCCATTTGACTCTTTCCACAACTATCCAACTCACAACGTGGCACTGG
GGTTGTGCCGCCTTTGCAGGTGTATCTTATACACGTGGCTTTTGGCCGCAGAGGC
ACCTGTCGCCAGGTGGGGGGTTCCGCTGCCTGCAAAGGGTCGCTACAGACGTT
GTTTGTCTTCAAGAAGCTTCCAGAGGAACTGCTTCCTTCACGACATTCAACAGA
CCTTGCATTCCTTTGGCGAGAGGGGAAAGACCCCTAGGAATGCTCGTCAAGAAG
ACAGGGCCAGGTTTCCGGGCCCTCACATTGCCAAAAGACGGCAATATGGTGGAA
AATAACATATAGACAAACGCACACCGGCCTTATTCCAAGCGGCTTCGGCCAGTA
ACGTTAGGGGGGGGGGAGGGAGAGGGGCGGCGTTAACTGCGCGGCCGTACAG
AACCTTGCTCAGAAGAACTCGTCAAGAAGGCGATAGAAGGCGATGCGCTGCGA
ATCGGGAGCGGCGATACCGTAAAGCACGAGGAAGCGGTCAGCCCATTCGCCGC
CAAGCTCTTCAGCAATATCACGGGTAGCCAACGCTATGTCCTGATAGCGGTCCGC
CACACCCAGCCGGCCACAGTCGATGAATCCAGAAAAGCGGCCATTTTCCACCAT
GATATTCGGCAAGCAGGCATCGCCATGGGTCACGACGAGATCATCGCCGTCGGG
CATGCGCGCCTTGAGCCTGGCGAACAGTTCGGCTGGCGCGAGCCCCTGATGCTC
TTCGTCCAGATCATCCTGATCGACAAGACCGGCTTCCATCCGAGTACGTGCTCGC
TCGATGCGATGTTTCGCTTGGTGGTCGAATGGGCAGGTAGCCGGATCAAGCGTAT
GCAGCCGCCGCATTGCATCAGCCATGATGGATACTTTCTCGGCAGGAGCAAGGT
GAGATGACAGGAGATCCTGCCCCGGCACTTCGCCCAATAGCAGCCAGTCCCTTC
CCGCTTCAGTGACAACGTCGAGCACAGCTGCGCAAGGAACGCCCGTCGTGGCC
AGCCACGATAGCCGCGCTGCCTCGTCCTGCAGTTCATTCAGGGCACCGGACAGG
TCGGTCTTGACAAAAAGAACCGGGCGCCCCTGCGCTGACAGCCGGAACACGGC
GGCATCAGAGCAGCCGATTGTCTGTTGTGCCCAGTCATAGCCGAATAGCCTCTCC
ACCCAAGCGGCCGGAGAACCTGCGTGCAATCCATCTTGTTCAATGGCCGATCCC
ATATTGGCTGCAGGGTCGCTCGGTGTTCGAGGCCACACGCGTCACCTTAATATGC
GAAGTGGACCTGGGACCGCGCCGCCCCGACTGCATCTGCGTGTTCGAATTAATT
CGCCAATGACAAGACGCTGGGCGGGGTTTGCTCGACATTGGGTGGAAACATTCC
AGGCCTGGGTGGAGAGGCTTTTTGCTTCCTCTTGCAAA

Humanized SKM9-2 L-chain (human Ig kappa) sequences
ATGGACATGCGGGTCCCTGCCCAGCTTCTGGGCTTGCTGCTCCTGTGGGTGCCC
GGAGCTAGGTGCGACGTCGTTATGACCCAGAGCCCACTGTCACTGCCCGTGACA
CTGGGACAACCTGCAAGCATATCCTGCAGATCTTCACAGAATATCGTGCATCAGA
ACGGGCAGACCTATCTTGGTTGGTACTTGCAGAAACCCGGGCAATCCCCTCAAC
TGCTGATCTTCAGAGTGAGCAACCGCTTTTCAGGCGTTCCTGATCGCTTTAGTGG

CTCAGGATCTGGGACGGACTTTACCCTGAAGATTAGCCGTGTCGAAGCCGAGGA
TGTTGGCGTCTACTACTGCTTCCAGGGTTCTCACATACCACGGACATTTGGTCAG
GGAACAAGACTGGAGATCAAGAGGACTGTCGCTGCTCCGAGTGTGTTCATCTTT
CCGCCATCCGATGAGCAGCTGAAGAGCGGAACTGCATCTGTGGTCTGCTTGCTC
AACAACTTCTATCCCAGGGAAGCTAAGGTGCAGTGGAAAGTGGACAATGCCCT
GCAATCCGGCAATTCCCAGGAAAGCGTGACAGAACAGGACAGCAAGGATTCTA
CCTACTCCCTGTCAAGCACCCTTACTCTGTCAAAGGCCGACTATGAGAAACACA
AGGTGTATGCGTGTGAGGTGACCCATCAAGGCCTCAGTAGTCCCGTGACGAAAA
GCTTCAATCGGGGAGAGTGTTAA

Humanized SKM9-2 L-chain (human Ig kappa) amino acid sequences
MDMRVPAQLLGLLLLWVPGARCDVVMTQSPLSLPVTLGQPASISCRSSQNIVHQNG
QTYLGWYLQKPGQSPQLLIFRVSNRFSGVPDRFSGSGSGTDFTLKISRVEAEDVGVY
YCFQGSHIPRTFGQGTRLEIKRTVAAPSVFIFPPSDEQLKSGTASVVCLLNNFYPREA
KVQWKVDNALQSGNSQESVTEQDSKDSTYSLSSTLTLSKADYEKHKVYACEVTHQ
GLSSPVTKSFNRGEC

Humanized SKM9-2 H-chain (human IgG1)
ATGGACTGGACCTGGAGAATCCTCTTCCTCGTAGCAGCAGCTACAGGTGCACAC
AGCCAGGTTCAGCTTGTGCAGAGCGGAGCCGAGGTCAAGAAACCAGGCGCTTC
TGTGAAAGTGTCATGCAAAGCTTCTGGCTTTAGCTTCACCACATACTGGATCACA
TGGGTGAGACAGGCTCCCGGGCAAGGGCTGGAATGGATTGGAATGATACACCCC
TCTGACGCCGAAACTCGACTTAGCCAGACTTTCAAGGACAAAGCCACACTGAC
CGCCGATAAGAGCACCAGCACCGCCTATATGGAGCTGAGTAGCCTGAGATCCGA
GGATACCGCCGTCTATTATTGTGCGCGGGATTTCGACGTATGGGGTCAAGGGACT
CTGGTTACTGTCTCAAGTGCGTCCACAAAGGGGCCTTCTGTGTTTCCTCTCGCTC
CCTCCAGTAAGTCAACTAGCGGAGGAACTGCCGCTTTGGGGTGTCTGGTGAAG
GACTACTTTCCAGAACCAGTAACCGTATCTTGGAATTCTGGTGCTCTGACGAGCG
GCGTGCATACCTTTCCTGCCGTGCTCCAGTCATCTGGTCTGTACTCCCTCTCAAG
CGTAGTGACAGTTCCGAGTTCCAGTCTGGGCACGCAGACATACATCTGCAATGT
GAACCACAAACCCTCTAACACAAAAGTGGATAAGCGCGTCGAGCCTAAATCTTG
CGACAAGACACACACTTGTCCGCCTTGTCCTGCACCAGAACTCCTGGGAGGTCC
CAGCGTATTCCTGTTTCCTCCAAAGCCTAAGGACACCCTCATGATTTCACGGACA
CCCGAGGTTACATGCGTTGTGGTGGATGTGTCCCACGAAGATCCCGAAGTGAAA
TTCAACTGGTATGTGGATGGTGTTGAGGTACACAATGCGAAAACGAAACCCAGG
GAAGAGCAGTACAACAGCACCTATAGGGTCGTCAGTGTCCTCACTGTGCTGCAT
CAGGACTGGTTGAATGGAAAGGAGTATAAATGCAAAGTCTCTAACAAGGCCCTT
CCCGCACCAATCGAAAAGACCATCAGCAAAGCAAAGGGGCAACCGAGAGAAC
CCCAGGTGTACACACTGCCACCTTCCCGCGAGGAGATGACCAAGAATCAGGTTA
GCCTCACGTGTCTGGTTAAGGGCTTTTACCCATCTGACATTGCCGTCGAGTGGGA
GTCAAATGGCCAACCCGAGAATAACTACAAAACCACCCCGCCTGTGTTGGACAG
CGATGGCTCCTTCTTTCTGTATAGCAAGCTGACTGTCGACAAGAGCCGTTGGCA
ACAGGGGAATGTGTTCAGCTGCTCCGTGATGCATGAAGCACTTCATAACCACTA
CACTCAGAAATCCCTGTCCTTGAGTCCAGGAAAGTAA

Humanized SKM9-2 H-chain (human IgG1) amino acid sequences

MDWTWRILFLVAAATGAHSQVQLVQSGAEVKKPGASVKVSCKASGFSFTTYWITW
VRQAPGQGLEWIGMIHPSDAETRLSQTFKDKATLTADKSTSTAYMELSSLRSEDTAV
YYCARDFDVWGQGTLVTVSSASTKGPSVFPLAPSSKSTSGGTAALGCLVKDYFPEP
VTVSWNSGALTSGVHTFPAVLQSSGLYSLSSVVTVPSSSLGTQTYICNVNHKPSNTK
VDKRVEPKSCDKTHTCPPCPAPELLGGPSVFLFPPKPKDTLMISRTPEVTCVVVDVS
HEDPEVKFNWYVDGVEVHNAKTKPREEQYNSTYRVVSVLTVLHQDWLNGKEYK
CKVSNKALPAPIEKTISKAKGQPREPQVYTLPPSREEMTKNQVSLTCLVKGFYPSDI
AVEWESNGQPENNYKTTPPVLDSDGSFFLYSKLTVDKSRWQQGNVFSCSVMHEAL
HNHYTQKSLSLSPGK

Supplementary Data 3

pCAE3.0 long product (CAE1+CAE2+: 2,089 bp) 12/12
GCTCTGACTGACCGCGTTACTCCCACGAGTGAGTTTTTCCATTTCATGCAACTG
TGCCCCATAGAGGAGAGAGGGCTCCATGTTGATTTAGTCTAATTTTGTTACAAA
TCTTATTTAGATAAATTGCCTCACTTATTTCTTTTGAGATCCAGCCCTGCATTTT
TCAGCCATCCTCTTTCACTCTACTTTTAGCCTTGGAAGACAGCCGAGAGCCAGG
CCAAGCACTAGGTGACAGATATCCACCATGGACATGCGGGTCCCTGCCCAGCT
TCTGGGCTTGCTGCTCCTGTGGGTGCCCGGAGCTAGGTGCGACGTCGTTATGAC
CCAGAGCCCACTGTCACTGCCCGTGACACTGGGACAACCTGCAAGCATATCCT
GCAGATCTTCACAGAATATCGTGCATCAGAACGGGCAGACCTATCTTGGTTGGT
ACTTGCAGAAACCCGGGCAATCCCCTCAACTGCTGATCTTCAGAGTGAGCAAC
CGCTTTTCAGGCGTTCCTGATCGCTTTAGTGGCTCAGGATCTGGGACGGACTTT
ACCCTGAAGATTAGCCGTGTCGAAGCCGAGGATGTTGGCGTCTACTACTGCTTC
CAGGGTTCTCACATACCACGGACATTTGGTCAGGGAACAAGACTGGAGATCAA
GAGGACTGTCGCTGCTCCGAGTGTGTTCATCTTTCCGCCATCCGATGAGCAGCT
GAAGAGCGGAACTGCATCTGTGGTCTGCTTGCTCAACAACTTCTATCCCAGGGA
AGCTAAGGTGCAGTGGAAAGTGGACAATGCCCTGCAATCCGGCAATTCCCAGG
AAAGCGTGACAGAACAGGACAGCAAGGATTCTACCTACTCCCTGTCAAGCACC
CTTACTCTGTCAAAGGCCGACTATGAGAAACACAAGGTGTATGCGTGTGAGGT
GACCCATCAAGGCCTCAGTAGTCCCGTGACGAAAAGCTTCAATCGGGGAGAGT
GTTAAGAATTCACGGTCACTGGCCGCCTCCAGTGCACTCGGAGGTGGGGTGTCT
TCCACAGTCTCTTTTAACACATCCCTTCAGCTCAGAATTATATTTGCTTGTGAAC
TAGTCTGTACAAAGGGGAGGCTGTTCTAACATGGGTGATAGCAACAAAACCGG
GCTGTTTTCACCCAGCCATGGGGCAATCCAGGGCCAAGGTCACTGACCTTCATG
CTCTCCCATCCCGTCACTGGTGAATGGCGATGACATTCATTTCCAAAAGCCTTG
GGACCCACGTGTCATTTCAGAGTTTTGTTGTTCTTTTATTTGACCTAACTTTGGC
TTTTCCGTTTACAGTCGCTCGTGAGGAATTGTATACTCGAGCCACCATGGACTG
GACCTGGAGAATCCTCTTCCTCGTAGCAGCAGCTACAGGTGCACACAGCCAGG
TTCAGCTTGTGCAGAGCGGAGCCGAGGTCAAGAAACCAGGCGCTTCTGTGAAA
GTGTCATGCAAAGCTTCTGGCTTTAGCTTCACCACATACTGGATCACATGGGTG
AGACAGGCTCCCGGGCAAGGGCTGGAATGGATTGGAATGATACACCCCTCTGA
CGCCGAAACTCGACTTAGCCAGACTTTCAAGGACAAAGCCACACTGACCGCCG
ATAAGAGCACCAGCACCGCCTATATGGAGCTGAGTAGCCTGAGATCCGAGGAT
ACCGCCGTCTATTATTGTGCGCGGGATTTCGACGTATGGGGTCAAGGGACTCTG
GTTACTGTCTCAAGTGCGTCCACAAAGGGGCCTTCTGTGTTTCCTCTCGCTCCCT
CCAGTAAGTCAACTAGCGGAGGAACTGCCGCTTTGGGGTGTCTGGTGAAGGAC
TACTTTCCAGAACCAGTAACCGTATCTTGGAATTCTGGTGCTCTGACGAGCGGC
GTGCATACCTTTCCTGCCGTGCTCCAGTCATCTGGTCTGTACTCCCTCTCAAGCG
TAGTGACAGTTCCGAGTTCCAGTCTGGGCACGCAGACATACATCTGCAATGTG
AACCACAAACCCTCTAACACAAAAGTGGATAAGCGCGTCGAGCCTAAATCTTG
CGACAAGACACACACTTGTCCGCCTTGTCCTGCACCAGAACTCCTGGGA

pCAE3.0 middle length products (CAE1-CAE2-: 1,629 bp) 12/16

GCTCTGACTGACCGCGTTACTCCCACGACCTTGGAAGACAGCCGAGAGCCAGG
CCAAGCACTAGGTGACAGATATCCACCATGGACATGCGGGTCCCTGCCCAGCT
TCTGGGCTTGCTGCTCCTGTGGGTGCCCGGAGCTAGGTGCGACGTCGTTATGAC
CCAGAGCCCACTGTCACTGCCCGTGACACTGGGACAACCTGCAAGCATATCCT
GCAGATCTTCACAGAATATCGTGCATCAGAACGGGCAGACCTATCTTGGTTGGT
ACTTGCAGAAACCCGGGCAATCCCCTCAACTGCTGATCTTCAGAGTGAGCAAC
CGCTTTTCAGGCGTTCCTGATCGCTTTAGTGGCTCAGGATCTGGGACGGACTTT
ACCCTGAAGATTAGCCGTGTCGAAGCCGAGGATGTTGGCGTCTACTACTGCTTC
CAGGGTTCTCACATACCACGGACATTTGGTCAGGGAACAAGACTGGAGATCAA
GAGGACTGTCGCTGCTCCGAGTGTGTTCATCTTTCCGCCATCCGATGAGCAGCT
GAAGAGCGGAACTGCATCTGTGGTCTGCTTGCTCAACAACTTCTATCCCAGGGA
AGCTAAGGTGCAGTGGAAAGTGGACAATGCCCTGCAATCCGGCAATTCCCAGG
AAAGCGTGACAGAACAGGACAGCAAGGATTCTACCTACTCCCTGTCAAGCACC
CTTACTCTGTCAAAGGCCGACTATGAGAAACACAAGGTGTATGCGTGTGAGGT
GACCCATCAAGGCCTCAGTAGTCCCGTGACGAAAAGCTTCAATCGGGGAGAGT
GTTAAGAATTCACGGTCACTGGCCGCCTCCAGTGCACTCGGAGTCGCTCGTGAG
GAATTGTATACTCGAGCCACCATGGACTGGACCTGGAGAATCCTCTTCCTCGTA
GCAGCAGCTACAGGTGCACACAGCCAGGTTCAGCTTGTGCAGAGCGGAGCCGA
GGTCAAGAAACCAGGCGCTTCTGTGAAAGTGTCATGCAAAGCTTCTGGCTTTA
GCTTCACCACATACTGGATCACATGGGTGAGACAGGCTCCCGGGCAAGGGCTG
GAATGGATTGGAATGATACACCCCTCTGACGCCGAAACTCGACTTAGCCAGAC
TTTCAAGGACAAAGCCACACTGACCGCCGATAAGAGCACCAGCACCGCCTATA
TGGAGCTGAGTAGCCTGAGATCCGAGGATACCGCCGTCTATTATTGTGCGCGG
GATTTCGACGTATGGGGTCAAGGGACTCTGGTTACTGTCTCAAGTGCGTCCACA
AAGGGGCCTTCTGTGTTTCCTCTCGCTCCCTCCAGTAAGTCAACTAGCGGAGGA
ACTGCCGCTTTGGGGTGTCTGGTGAAGGACTACTTTCCAGAACCAGTAACCGTA
TCTTGGAATTCTGGTGCTCTGACGAGCGGCGTGCATACCTTTCCTGCCGTGCTC
CAGTCATCTGGTCTGTACTCCCTCTCAAGCGTAGTGACAGTTCCGAGTTCCAGT
CTGGGCACGCAGACATACATCTGCAATGTGAACCACAAACCCTCTAACACAAA
AGTGGATAAGCGCGTCGAGCCTAAATCTTGCGACAAGACACACACTTGTCCGC
CTTGTCCTGCACCAGAACTCCTGGGA

pCAE3.0 middle length products (CAE1-CAE2- partial L Δ786: 1,516 bp) 4/16
GCTCTGACTGACCGCGTTACTCCCACGACCTTGGAAGACAGCCGAGAGCCAGG
CCAAGCACTAGGTGACAGATATCCACCATGGACATGCGGGTCCCTGCCCAGCT
TCTGGGCTTGCTGCTCCTGTGGGTGCCCGGAGCTAGGTGCGACGTCGTTATGAC
CCAGAGCCCACTGTCACTGCCCGTGACACTGGGACAACCTGCAAGCATATCCT
GCAGATCTTCACAGAATATCGTGCATCAGAACGGGCAGACCTATCTTGGTTGGT
ACTTGCAGAAACCCGGGCAATCCCCTCAACTGCTGATCTTCAGAGTGAGCAAC
CGCTTTTCAGGCGTTCCTGATCGCTTTAGTGGCTCAGGATCTGGGACGGACTTT
ACCCTGAAGATTAGCCGTGTCGAAGCCGAGGATGTTGGCGTCTACTACTGCTTC
CAGGGTTCTCACATACCACGGACATTTGGTCAGGGAACAAGACTGGAGATCAA
GAGGACTGTCGCTGCTCCGAGTGTGTTCATCTTTCCGCCATCCGATGAGCAGCT
GAAGAGCGGAACTGCATCTGTGGTCTGCTTGCTCAACAACTTCTATCCCAGGGA

AGCTAAGGTGCAGTGGAAAGTGGACAATGCCCTGCAATCCGGCAATTCCCAGG
AAAGCGTGACAGAACAGGACAGCAAGGATTCTACCTACTCCCTGTCAAGCACC
CTTACTCTGTCAAAGGCCGACTATGAGAAACACAAGTCGCTCGTGAGGAATTG
TATACTCGAGCCACCATGGACTGGACCTGGAGAATCCTCTTCCTCGTAGCAGCA
GCTACAGGTGCACACAGCCAGGTTCAGCTTGTGCAGAGCGGAGCCGAGGTCAA
GAAACCAGGCGCTTCTGTGAAAGTGTCATGCAAAGCTTCTGGCTTTAGCTTCAC
CACATACTGGATCACATGGGTGAGACAGGCTCCCGGGCAAGGGCTGGAATGGA
TTGGAATGATACACCCCTCTGACGCCGAAACTCGACTTAGCCAGACTTTCAAGG
ACAAAGCCACACTGACCGCCGATAAGAGCACCAGCACCGCCTATATGGAGCTG
AGTAGCCTGAGATCCGAGGATACCGCCGTCTATTATTGTGCGCGGGATTTCGAC
GTATGGGGTCAAGGGACTCTGGTTACTGTCTCAAGTGCGTCCACAAAGGGGCC
TTCTGTGTTTCCTCTCGCTCCCTCCAGTAAGTCAACTAGCGGAGGAACTGCCGC
TTTGGGGTGTCTGGTGAAGGACTACTTTCCAGAACCAGTAACCGTATCTTGGAA
TTCTGGTGCTCTGACGAGCGGCGTGCATACCTTTCCTGCCGTGCTCCAGTCATC
TGGTCTGTACTCCCTCTCAAGCGTAGTGACAGTTCCGAGTTCCAGTCTGGGCAC
GCAGACATACATCTGCAATGTGAACCACAAACCCTCTAACACAAAAGTGGATA
AGCGCGTCGAGCCTAAATCTTGCGACAAGACACACACTTGTCCGCCTTGTCCTG
CACCAGAACTCCTGGGA

pCAE3.0 short products (H-chain: 813bp) 16/16
GCTCTGACTGACCGCGTTACTCCCACGATCGCTCGTGAGGAATTGTATACTCGA
GCCACCATGGACTGGACCTGGAGAATCCTCTTCCTCGTAGCAGCAGCTACAGG
TGCACACAGCCAGGTTCAGCTTGTGCAGAGCGGAGCCGAGGTCAAGAAACCAG
GCGCTTCTGTGAAAGTGTCATGCAAAGCTTCTGGCTTTAGCTTCACCACATACT
GGATCACATGGGTGAGACAGGCTCCCGGGCAAGGGCTGGAATGGATTGGAATG
ATACACCCCTCTGACGCCGAAACTCGACTTAGCCAGACTTTCAAGGACAAAGC
CACACTGACCGCCGATAAGAGCACCAGCACCGCCTATATGGAGCTGAGTAGCC
TGAGATCCGAGGATACCGCCGTCTATTATTGTGCGCGGGATTTCGACGTATGGG
GTCAAGGGACTCTGGTTACTGTCTCAAGTGCGTCCACAAAGGGGCCTTCTGTGT
TTCCTCTCGCTCCCTCCAGTAAGTCAACTAGCGGAGGAACTGCCGCTTTGGGGT
GTCTGGTGAAGGACTACTTTCCAGAACCAGTAACCGTATCTTGGAATTCTGGTG
CTCTGACGAGCGGCGTGCATACCTTTCCTGCCGTGCTCCAGTCATCTGGTCTGT
ACTCCCTCTCAAGCGTAGTGACAGTTCCGAGTTCCAGTCTGGGCACGCAGACAT
ACATCTGCAATGTGAACCACAAACCCTCTAACACAAAAGTGGATAAGCGCGTC
GAGCCTAAATCTTGCGACAAGACACACACTTGTCCGCCTTGTCCTGCACCAGAA
CTCCTGGGA

pCAE3.1 long product (CAE1+CAE2+: 2,017 bp) 15/15
GCTCTGACTGACCGCGTTACTCCCACGAGTGAGTTTTTCCATTTCATGCAACTG
TGCCCCATAGAGGAGAGAGGGTCCAGCCCTGCATTTTTCAGCCATCCTCTTTCA
CTCTACTTTTAGCCTTGGAAGACAGCCGAGAGCCAGGCCAAGCACTAGGTGAC
AGATATCCACCATGGACATGCGGGTCCCTGCCCAGCTTCTGGGCTTGCTGCTCC
TGTGGGTGCCCGGAGCTAGGTGCGACGTCGTTATGACCCAGAGCCCACTGTCA
CTGCCCGTGACACTGGGACAACCTGCAAGCATATCCTGCAGATCTTCACAGAA

TATCGTGCATCAGAACGGGCAGACCTATCTTGGTTGGTACTTGCAGAAACCCG
GGCAATCCCCTCAACTGCTGATCTTCAGAGTGAGCAACCGCTTTTCAGGCGTTC
CTGATCGCTTTAGTGGCTCAGGATCTGGGACGGACTTTACCCTGAAGATTAGCC
GTGTCGAAGCCGAGGATGTTGGCGTCTACTACTGCTTCCAGGGTTCTCACATAC
CACGGACATTTGGTCAGGGAACAAGACTGGAGATCAAGAGGACTGTCGCTGCT
CCGAGTGTGTTCATCTTTCCGCCATCCGATGAGCAGCTGAAGAGCGGAACTGC
ATCTGTGGTCTGCTTGCTCAACAACTTCTATCCCAGGGAAGCTAAGGTGCAGTG
GAAAGTGGACAATGCCCTGCAATCCGGCAATTCCCAGGAAAGCGTGACAGAAC
AGGACAGCAAGGATTCTACCTACTCCCTGTCAAGCACCCTTACTCTGTCAAAGG
CCGACTATGAGAAACACAAGGTGTATGCGTGTGAGGTGACCCATCAAGGCCTC
AGTAGTCCCGTGACGAAAAGCTTCAATCGGGGAGAGTGTTAAGAATTCACGGT
CACTGGCCGCCTCCAGTGCACTCGGAGGTGGGGTGTCTTCCACAGTCTCTTTA
ACACATCCCTTCAGCTCAGAATTATATTTGCTTGTGAACTAGTCTGTACAAAGG
GGAGGCTGTTCTAACATGGGTGATAGCAACAAAACCGGGCTGTTTTCACCCAG
CCATGGGGCAATCCAGGGCCAAGGTCACTGACCTTCATGCTCTCCCATCCCGTC
ACTGGTGAATGGCGATGACATTCATTTCCAAAAGCCTTGGGACCCACGTGTCAT
TTCAGAGTTTTGTTGTTCTTTTATTTGACCTAACTTTGGCTTTTCCGTTTACAGTC
GCTCGTGAGGAATTGTATACTCGAGCCACCATGGACTGGACCTGGAGAATCCT
CTTCCTCGTAGCAGCAGCTACAGGTGCACACAGCCAGGTTCAGCTTGTGCAGA
GCGGAGCCGAGGTCAAGAAACCAGGCGCTTCTGTGAAAGTGTCATGCAAAGCT
TCTGGCTTTAGCTTCACCACATACTGGATCACATGGGTGAGACAGGCTCCCGGG
CAAGGGCTGGAATGGATTGGAATGATACACCCCTCTGACGCCGAAACTCGACT
TAGCCAGACTTTCAAGGACAAAGCCACACTGACCGCCGATAAGAGCACCAGCA
CCGCCTATATGGAGCTGAGTAGCCTGAGATCCGAGGATACCGCCGTCTATTATT
GTGCGCGGGATTTCGACGTATGGGGTCAAGGGACTCTGGTTACTGTCTCAAGTG
CGTCCACAAAGGGGCCTTCTGTGTTTCCTCTCGCTCCCTCCAGTAAGTCAACTA
GCGGAGGAACTGCCGCTTTGGGGTGTCTGGTGAAGGACTACTTTCCAGAACCA
GTAACCGTATCTTGGAATTCTGGTGCTCTGACGAGCGGCGTGCATACCTTTCCT
GCCGTGCTCCAGTCATCTGGTCTGTACTCCCTCTCAAGCGTAGTGACAGTTCCG
AGTTCCAGTCTGGGCACGCAGACATACATCTGCAATGTGAACCACAAACCCTC
TAACACAAAAGTGGATAAGCGCGTCGAGCCTAAATCTTGCGACAAGACACACA
CTTGTCCGCCTTGTCCTGCACCAGAACTCCTGGGA

pCAE3.1 middle length products (CAE1-CAE2-: 1,629 bp) 10/14
GCTCTGACTGACCGCGTTACTCCCACGACCTTGGAAGACAGCCGAGAGCCAGG
CCAAGCACTAGGTGACAGATATCCACCATGGACATGCGGGTCCCTGCCCAGCT
TCTGGGCTTGCTGCTCCTGTGGGTGCCCGGAGCTAGGTGCGACGTCGTTATGAC
CCAGAGCCCACTGTCACTGCCCGTGACACTGGGACAACCTGCAAGCATATCCT
GCAGATCTTCACAGAATATCGTGCATCAGAACGGGCAGACCTATCTTGGTTGGT
ACTTGCAGAAACCCGGGCAATCCCCTCAACTGCTGATCTTCAGAGTGAGCAAC
CGCTTTTCAGGCGTTCCTGATCGCTTTAGTGGCTCAGGATCTGGGACGGACTTT
ACCCTGAAGATTAGCCGTGTCGAAGCCGAGGATGTTGGCGTCTACTACTGCTTC
CAGGGTTCTCACATACCACGGACATTTGGTCAGGGAACAAGACTGGAGATCAA
GAGGACTGTCGCTGCTCCGAGTGTGTTCATCTTTCCGCCATCCGATGAGCAGCT

GAAGAGCGGAACTGCATCTGTGGTCTGCTTGCTCAACAACTTCTATCCCAGGGA
AGCTAAGGTGCAGTGGAAAGTGGACAATGCCCTGCAATCCGGCAATTCCCAGG
AAAGCGTGACAGAACAGGACAGCAAGGATTCTACCTACTCCCTGTCAAGCACC
CTTACTCTGTCAAAGGCCGACTATGAGAAACACAAGGTGTATGCGTGTGAGGT
GACCCATCAAGGCCTCAGTAGTCCCGTGACGAAAAGCTTCAATCGGGGAGAGT
GTTAAGAATTCACGGTCACTGGCCGCCTCCAGTGCACTCGGAGTCGCTCGTGAG
GAATTGTATACTCGAGCCACCATGGACTGGACCTGGAGAATCCTCTTCCTCGTA
GCAGCAGCTACAGGTGCACACAGCCAGGTTCAGCTTGTGCAGAGCGGAGCCGA
GGTCAAGAAACCAGGCGCTTCTGTGAAAGTGTCATGCAAAGCTTCTGGCTTTA
GCTTCACCACATACTGGATCACATGGGTGAGACAGGCTCCCGGGCAAGGGCTG
GAATGGATTGGAATGATACACCCCTCTGACGCCGAAACTCGACTTAGCCAGAC
TTTCAAGGACAAAGCCACACTGACCGCCGATAAGAGCACCAGCACCGCCTATA
TGGAGCTGAGTAGCCTGAGATCCGAGGATACCGCCGTCTATTATTGTGCGCGG
GATTTCGACGTATGGGGTCAAGGGACTCTGGTTACTGTCTCAAGTGCGTCCACA
AAGGGGCCTTCTGTGTTTCCTCTCGCTCCCTCCAGTAAGTCAACTAGCGGAGGA
ACTGCCGCTTTGGGGTGTCTGGTGAAGGACTACTTTCCAGAACCAGTAACCGTA
TCTTGGAATTCTGGTGCTCTGACGAGCGGCGTGCATACCTTTCCTGCCGTGCTC
CAGTCATCTGGTCTGTACTCCCTCTCAAGCGTAGTGACAGTTCCGAGTTCCAGT
CTGGGCACGCAGACATACATCTGCAATGTGAACCACAAACCCTCTAACACAAA
AGTGGATAAGCGCGTCGAGCCTAAATCTTGCGACAAGACACACACTTGTCCGC
CTTGTCCTGCACCAGAACTCCTGGGA

pCAE3.1 middle length products (CAE1-CAE2- partial L Δ786: 1,516 bp) 2/14
GCTCTGACTGACCGCGTTACTCCCACGACCTTGGAAGACAGCCGAGAGCCAGG
CCAAGCACTAGGTGACAGATATCCACCATGGACATGCGGGTCCCTGCCCAGCT
TCTGGGCTTGCTGCTCCTGTGGGTGCCCGGAGCTAGGTGCGACGTCGTTATGAC
CCAGAGCCCACTGTCACTGCCCGTGACACTGGGACAACCTGCAAGCATATCCT
GCAGATCTTCACAGAATATCGTGCATCAGAACGGGCAGACCTATCTTGGTTGGT
ACTTGCAGAAACCCGGGCAATCCCCTCAACTGCTGATCTTCAGAGTGAGCAAC
CGCTTTTCAGGCGTTCCTGATCGCTTTAGTGGCTCAGGATCTGGGACGGACTTT
ACCCTGAAGATTAGCCGTGTCGAAGCCGAGGATGTTGGCGTCTACTACTGCTTC
CAGGGTTCTCACATACCACGGACATTTGGTCAGGGAACAAGACTGGAGATCAA
GAGGACTGTCGCTGCTCCGAGTGTGTTCATCTTTCCGCCATCCGATGAGCAGCT
GAAGAGCGGAACTGCATCTGTGGTCTGCTTGCTCAACAACTTCTATCCCAGGGA
AGCTAAGGTGCAGTGGAAAGTGGACAATGCCCTGCAATCCGGCAATTCCCAGG
AAAGCGTGACAGAACAGGACAGCAAGGATTCTACCTACTCCCTGTCAAGCACC
CTTACTCTGTCAAAGGCCGACTATGAGAAACACAAGTCGCTCGTGAGGAATTG
TATACTCGAGCCACCATGGACTGGACCTGGAGAATCCTCTTCCTCGTAGCAGCA
GCTACAGGTGCACACAGCCAGGTTCAGCTTGTGCAGAGCGGAGCCGAGGTCAA
GAAACCAGGCGCTTCTGTGAAAGTGTCATGCAAAGCTTCTGGCTTTAGCTTCAC
CACATACTGGATCACATGGGTGAGACAGGCTCCCGGGCAAGGGCTGGAATGGA
TTGGAATGATACACCCCTCTGACGCCGAAACTCGACTTAGCCAGACTTTCAAGG
ACAAAGCCACACTGACCGCCGATAAGAGCACCAGCACCGCCTATATGGAGCTG
AGTAGCCTGAGATCCGAGGATACCGCCGTCTATTATTGTGCGCGGGATTTCGAC

GTATGGGGTCAAGGGACTCTGGTTACTGTCTCAAGTGCGTCCACAAAGGGGCC
TTCTGTGTTTCCTCTCGCTCCCTCCAGTAAGTCAACTAGCGGAGGAACTGCCGC
TTTGGGGTGTCTGGTGAAGGACTACTTTCCAGAACCAGTAACCGTATCTTGGAA
TTCTGGTGCTCTGACGAGCGGCGTGCATACCTTTCCTGCCGTGCTCCAGTCATC
TGGTCTGTACTCCCTCTCAAGCGTAGTGACAGTTCCGAGTTCCAGTCTGGGCAC
GCAGACATACATCTGCAATGTGAACCACAAACCCTCTAACACAAAAGTGGATA
AGCGCGTCGAGCCTAAATCTTGCGACAAGACACACACTTGTCCGCCTTGTCCTG
CACCAGAACTCCTGGGA

pCAE3.1 middle length products (CAE1-CAE2- partial L Δ651: 1,381 bp) 2/14
GCTCTGACTGACCGCGTTACTCCCACGACCTTGGAAGACAGCCGAGAGCCAGG
CCAAGCACTAGGTGACAGATATCCACCATGGACATGCGGGTCCCTGCCCAGCT
TCTGGGCTTGCTGCTCCTGTGGGTGCCCGGAGCTAGGTGCGACGTCGTTATGAC
CCAGAGCCCACTGTCACTGCCCGTGACACTGGGACAACCTGCAAGCATATCCT
GCAGATCTTCACAGAATATCGTGCATCAGAACGGGCAGACCTATCTTGGTTGGT
ACTTGCAGAAACCCGGGCAATCCCCTCAACTGCTGATCTTCAGAGTGAGCAAC
CGCTTTTCAGGCGTTCCTGATCGCTTTAGTGGCTCAGGATCTGGGACGGACTTT
ACCCTGAAGATTAGCCGTGTCGAAGCCGAGGATGTTGGCGTCTACTACTGCTTC
CAGGGTTCTCACATACCACGGACATTTGGTCAGGGAACAAGACTGGAGATCAA
GAGGACTGTCGCTGCTCCGAGTGTGTTCATCTTTCCGCCATCCGATGAGCAGCT
GAAGAGCGGAACTGCATCTGTGGTCTGCTTGCTCAACAACTTCTATCCCAGGGA
AGCTAAGTCGCTCGTGAGGAATTGTATACTCGAGCCACCATGGACTGGACCTG
GAGAATCCTCTTCCTCGTAGCAGCAGCTACAGGTGCACACAGCCAGGTTCAGC
TTGTGCAGAGCGGAGCCGAGGTCAAGAAACCAGGCGCTTCTGTGAAAGTGTCA
TGCAAAGCTTCTGGCTTTAGCTTCACCACATACTGGATCACATGGGTGAGACAG
GCTCCCGGGCAAGGGCTGGAATGGATTGGAATGATACACCCCTCTGACGCCGA
AACTCGACTTAGCCAGACTTTCAAGGACAAAGCCACACTGACCGCCGATAAGA
GCACCAGCACCGCCTATATGGAGCTGAGTAGCCTGAGATCCGAGGATACCGCC
GTCTATTATTGTGCGCGGGATTTCGACGTATGGGGTCAAGGGACTCTGGTTACT
GTCTCAAGTGCGTCCACAAAGGGGCCTTCTGTGTTTCCTCTCGCTCCCTCCAGT
AAGTCAACTAGCGGAGGAACTGCCGCTTTGGGGTGTCTGGTGAAGGACTACTT
TCCAGAACCAGTAACCGTATCTTGGAATTCTGGTGCTCTGACGAGCGGCGTGCA
TACCTTTCCTGCCGTGCTCCAGTCATCTGGTCTGTACTCCCTCTCAAGCGTAGTG
ACAGTTCCGAGTTCCAGTCTGGGCACGCAGACATACATCTGCAATGTGAACCA
CAAACCCTCTAACACAAAAGTGGATAAGCGCGTCGAGCCTAAATCTTGCGACA
AGACACACACTTGTCCGCCTTGTCCTGCACCAGAACTCCTGGGA

pCAE3.1 short products (H:813) 4/4
GCTCTGACTGACCGCGTTACTCCCACGATCGCTCGTGAGGAATTGTATACTCGA
GCCACCATGGACTGGACCTGGAGAATCCTCTTCCTCGTAGCAGCAGCTACAGG
TGCACACAGCCAGGTTCAGCTTGTGCAGAGCGGAGCCGAGGTCAAGAAACCAG
GCGCTTCTGTGAAAGTGTCATGCAAAGCTTCTGGCTTTAGCTTCACCACATACT
GGATCACATGGGTGAGACAGGCTCCCGGGCAAGGGCTGGAATGGATTGGAATG
ATACACCCCTCTGACGCCGAAACTCGACTTAGCCAGACTTTCAAGGACAAAGC

CACACTGACCGCCGATAAGAGCACCAGCACCGCCTATATGGAGCTGAGTAGCC
TGAGATCCGAGGATACCGCCGTCTATTATTGTGCGCGGATTTCGACGTATGGG
GTCAAGGGACTCTGGTTACTGTCTCAAGTGCGTCCACAAAGGGGCCTTCTGTGT
TTCCTCTCGCTCCCTCCAGTAAGTCAACTAGCGGAGGAACTGCCGCTTTGGGGT
GTCTGGTGAAGGACTACTTTCCAGAACCAGTAACCGTATCTTGGAATTCTGGTG
CTCTGACGAGCGGCGTGCATACCTTTCCTGCCGTGCTCCAGTCATCTGGTCTGT
ACTCCCTCTCAAGCGTAGTGACAGTTCCGAGTTCCAGTCTGGGCACGCAGACAT
ACATCTGCAATGTGAACCACAAACCCTCTAACACAAAAGTGGATAAGCGCGTC
GAGCCTAAATCTTGCGACAAGACACACACTTGTCCGCCTTGTCCTGCACCAGAA
CTCCTGGGA

pCAE3.31 long product (CAE1+CAE2+: 1,775 bp) 9/16
GCTCTGACTGACCGCGTTACTCCCACGAGTGAGTTTTTCCAGCCCTGCATTTTTC
AGCCATCCTCTTTCACTCTACTTTTAGCCTTGGAAGACAGCCGAGAGCCAGGCC
AAGCACTAGGTGACAGATATCCACCATGGACATGCGGGTCCCTGCCCAGCTTC
TGGGCTTGCTGCTCCTGTGGGTGCCCGGAGCTAGGTGCGACGTCGTTATGACCC
AGAGCCCACTGTCACTGCCCGTGACACTGGGACAACCTGCAAGCATATCCTGC
AGATCTTCACAGAATATCGTGCATCAGAACGGGCAGACCTATCTTGGTTGGTAC
TTGCAGAAACCCGGGCAATCCCCTCAACTGCTGATCTTCAGAGTGAGCAACCG
CTTTTCAGGCGTTCCTGATCGCTTTAGTGGCTCAGGATCTGGGACGGACTTTAC
CCTGAAGATTAGCCGTGTCGAAGCCGAGGATGTTGGCGTCTACTACTGCTTCCA
GGGTTCTCACATACCACGGACATTTGGTCAGGGAACAAGACTGGAGATCAAGA
GGACTGTCGCTGCTCCGAGTGTGTTCATCTTTCCGCCATCCGATGAGCAGCTGA
AGAGCGGAACTGCATCTGTGGTCTGCTTGCTCAACAACTTCTATCCCAGGGAAG
CTAAGGTGCAGTGGAAAGTGGACAATGCCCTGCAATCCGGCAATTCCCAGGAA
AGCGTGACAGAACAGGACAGCAAGGATTCTACCTACTCCCTGTCAAGCACCCT
TACTCTGTCAAAGGCCGACTATGAGAAACACAAGGTGTATGCGTGTGAGGTGA
CCCATCAAGGCCTCAGTAGTCCCGTGACGAAAAGCTTCAATCGGGGAGAGTGT
TAAGAATTCACGGTCACTGGCCGCCTCCAGTGCACTCGGAGGTGGGGTGTCTTC
CACAGTCTCTTTTAACACATCCCTTCAGCTAGTTTTGTTGTTCTTTTATTTGACCT
AACTTTGGCTTTTCCGTTTACAGTCGCTCGTGAGGAATTGTATACTCGAGCCAC
CATGGACTGGACCTGGAGAATCCTCTTCCTCGTAGCAGCAGCTACAGGTGCAC
ACAGCCAGGTTCAGCTTGTGCAGAGCGGAGCCGAGGTCAAGAAACCAGGCGCT
TCTGTGAAAGTGTCATGCAAAGCTTCTGGCTTTAGCTTCACCACATACTGGATC
ACATGGGTGAGACAGGCTCCCGGGCAAGGGCTGGAATGGATTGGAATGATACA
CCCCTCTGACGCCGAAACTCGACTTAGCCAGACTTTCAAGGACAAAGCCACAC
TGACCGCCGATAAGAGCACCAGCACCGCCTATATGGAGCTGAGTAGCCTGAGA
TCCGAGGATACCGCCGTCTATTATTGTGCGCGGATTTCGACGTATGGGGTCAA
GGGACTCTGGTTACTGTCTCAAGTGCGTCCACAAAGGGGCCTTCTGTGTTTCCT
CTCGCTCCCTCCAGTAAGTCAACTAGCGGAGGAACTGCCGCTTTGGGGTGTCTG
GTGAAGGACTACTTTCCAGAACCAGTAACCGTATCTTGGAATTCTGGTGCTCTG
ACGAGCGGCGTGCATACCTTTCCTGCCGTGCTCCAGTCATCTGGTCTGTACTCC
CTCTCAAGCGTAGTGACAGTTCCGAGTTCCAGTCTGGGCACGCAGACATACATC
TGCAATGTGAACCACAAACCCTCTAACACAAAAGTGGATAAGCGCGTCGAGCC

TAAATCTTGCGACAAGACACACACTTGTCCGCCTTGTCCTGCACCAGAACTCCT
GGGA

pCAE3.31 long product (CAE1+CAE2-: 1,683 bp) 7/16
GCTCTGACTGACCGCGTTACTCCCACGAGTGAGTTTTTCCAGCCCTGCATTTTTC
AGCCATCCTCTTTCACTCTACTTTTAGCCTTGGAAGACAGCCGAGAGCCAGGCC
AAGCACTAGGTGACAGATATCCACCATGGACATGCGGGTCCCTGCCCAGCTTC
TGGGCTTGCTGCTCCTGTGGGTGCCCGGAGCTAGGTGCGACGTCGTTATGACCC
AGAGCCCACTGTCACTGCCCGTGACACTGGGACAACCTGCAAGCATATCCTGC
AGATCTTCACAGAATATCGTGCATCAGAACGGGCAGACCTATCTTGGTTGGTAC
TTGCAGAAACCCGGGCAATCCCCTCAACTGCTGATCTTCAGAGTGAGCAACCG
CTTTTCAGGCGTTCCTGATCGCTTTAGTGGCTCAGGATCTGGGACGGACTTTAC
CCTGAAGATTAGCCGTGTCGAAGCCGAGGATGTTGGCGTCTACTACTGCTTCCA
GGGTTCTCACATACCACGGACATTTGGTCAGGGAACAAGACTGGAGATCAAGA
GGACTGTCGCTGCTCCGAGTGTGTTCATCTTTCCGCCATCCGATGAGCAGCTGA
AGAGCGGAACTGCATCTGTGGTCTGCTTGCTCAACAACTTCTATCCCAGGGAAG
CTAAGGTGCAGTGGAAAGTGGACAATGCCCTGCAATCCGGCAATTCCCAGGAA
AGCGTGACAGAACAGGACAGCAAGGATTCTACCTACTCCCTGTCAAGCACCCT
TACTCTGTCAAAGGCCGACTATGAGAAACACAAGGTGTATGCGTGTGAGGTGA
CCCATCAAGGCCTCAGTAGTCCCGTGACGAAAAGCTTCAATCGGGGAGAGTGT
TAAGAATTCACGGTCACTGGCCGCCTCCAGTGCACTCGGAGTCGCTCGTGAGG
AATTGTATACTCGAGCCACCATGGACTGGACCTGGAGAATCCTCTTCCTCGTAG
CAGCAGCTACAGGTGCACACAGCCAGGTTCAGCTTGTGCAGAGCGGAGCCGAG
GTCAAGAAACCAGGCGCTTCTGTGAAAGTGTCATGCAAAGCTTCTGGCTTTAGC
TTCACCACATACTGGATCACATGGGTGAGACAGGCTCCCGGGCAAGGGCTGGA
ATGGATTGGAATGATACACCCCTCTGACGCCGAAACTCGACTTAGCCAGACTTT
CAAGGACAAAGCCACACTGACCGCCGATAAGAGCACCAGCACCGCCTATATGG
AGCTGAGTAGCCTGAGATCCGAGGATACCGCCGTCTATTATTGTGCGCGGGATT
TCGACGTATGGGGTCAAGGGACTCTGGTTACTGTCTCAAGTGCGTCCACAAAG
GGGCCTTCTGTGTTTCCTCTCGCTCCCTCCAGTAAGTCAACTAGCGGAGGAACT
GCCGCTTTGGGGTGTCTGGTGAAGGACTACTTTCCAGAACCAGTAACCGTATCT
TGGAATTCTGGTGCTCTGACGAGCGGCGTGCATACCTTTCCTGCCGTGCTCCAG
TCATCTGGTCTGTACTCCCTCTCAAGCGTAGTGACAGTTCCGAGTTCCAGTCTG
GGCACGCAGACATACATCTGCAATGTGAACCACAAACCCTCTAACACAAAAGT
GGATAAGCGCGTCGAGCCTAAATCTTGCGACAAGACACACACTTGTCCGCCTT
GTCCTGCACCAGAACTCCTGGGA

pCAE3.31 short products (H:813) 4/4
GCTCTGACTGACCGCGTTACTCCCACGATCGCTCGTGAGGAATTGTATACTCGA
GCCACCATGGACTGGACCTGGAGAATCCTCTTCCTCGTAGCAGCAGCTACAGG
TGCACACAGCCAGGTTCAGCTTGTGCAGAGCGGAGCCGAGGTCAAGAAACCAG
GCGCTTCTGTGAAAGTGTCATGCAAAGCTTCTGGCTTTAGCTTCACCACATACT
GGATCACATGGGTGAGACAGGCTCCCGGGCAAGGGCTGGAATGGATTGGAATG
ATACACCCCTCTGACGCCGAAACTCGACTTAGCCAGACTTTCAAGGACAAAGC

CACACTGACCGCCGATAAGAGCACCAGCACCGCCTATATGGAGCTGAGTAGCC
TGAGATCCGAGGATACCGCCGTCTATTATTGTGCGCGGGATTTCGACGTATGGG
GTCAAGGGACTCTGGTTACTGTCTCAAGTGCGTCCACAAAGGGGCCTTCTGTGT
TTCCTCTCGCTCCCTCCAGTAAGTCAACTAGCGGAGGAACTGCCGCTTTGGGGT
GTCTGGTGAAGGACTACTTTCCAGAACCAGTAACCGTATCTTGGAATTCTGGTG
CTCTGACGAGCGGCGTGCATACCTTTCCTGCCGTGCTCCAGTCATCTGGTCTGT
ACTCCCTCTCAAGCGTAGTGACAGTTCCGAGTTCCAGTCTGGGCACGCAGACAT
ACATCTGCAATGTGAACCACAAACCCTCTAACACAAAAGTGGATAAGCGCGTC
GAGCCTAAATCTTGCGACAAGACACACACTTGTCCGCCTTGTCCTGCACCAGAA
CTCCTGGGA

Supplementary Data 4

RT-PCR

Total RNA was extracted with TRIzol Reagent (Invitrogen) from CHO/dhfr- transfected with pCAE3.31CMVs after 2 d. After treating with RQ1 RNase-free DNase (Promega K.K.) and ice-cooling, the RNA (25 ng per 20 µL of reaction volume) was reverse transcribed and amplified as cDNA with the OneStep RT-PCR kit (Qiagen GmbH) with 0.6 µM primers (forward: 5'-GCTCTGACTGACCGCGTTACTCCCA-3', reverse: 5'-TCCCAGGAGTTCTGGTGCAGGACAA-3'). Reverse transcription: 45°C, 30 min. PCR: 95°C, 15 min; 32 or 34 cycles of 94°C, 10 s; 59°C, 30 s; 68°C, 3 min. Products were resolved on 1.3% agarose gels. 1 kbp DNA Ladder One (Nacalai Tesque) was used as marker.

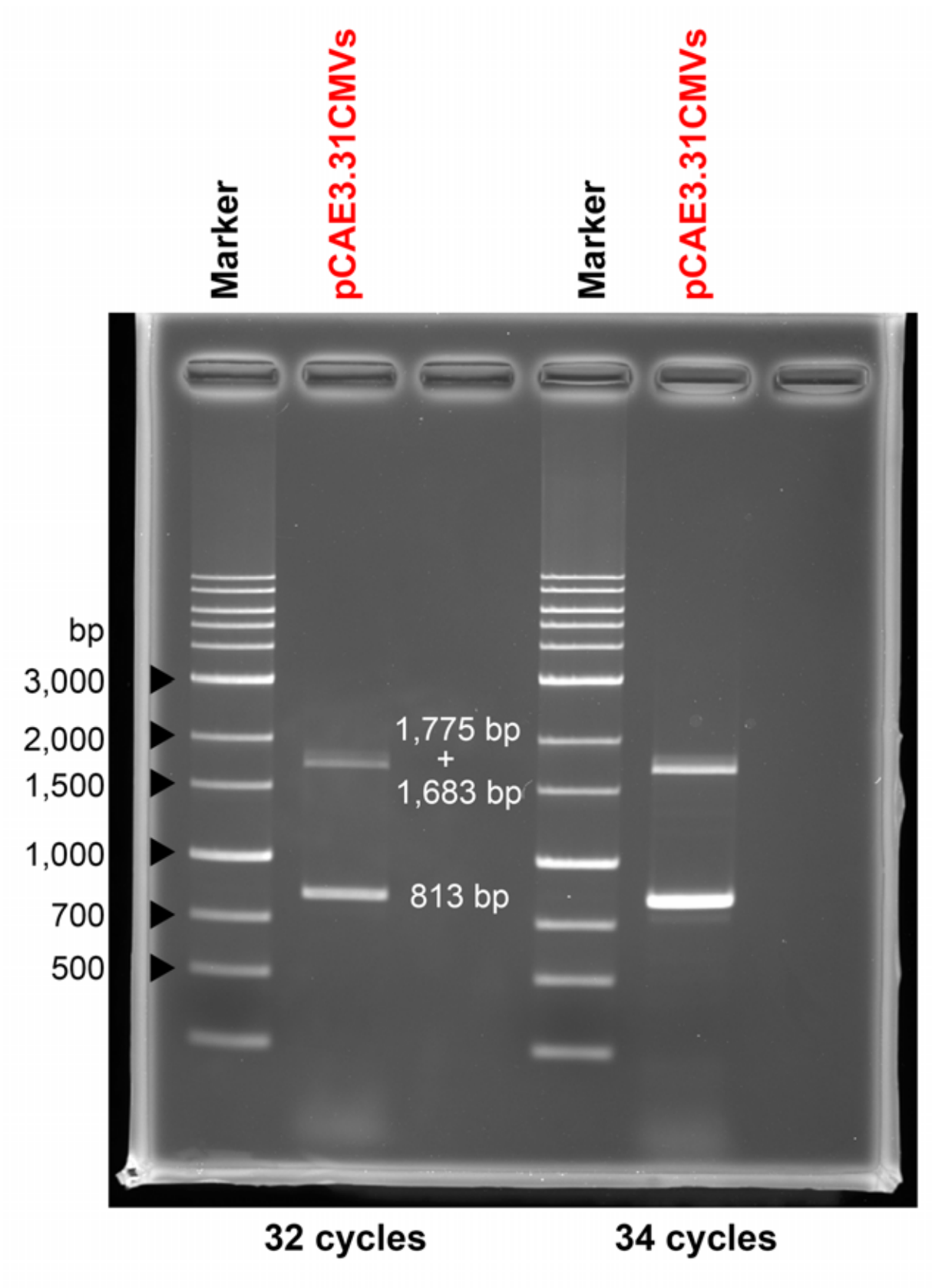

Real-time quantitative PCR.

The same RNase-free DNase-treated total RNA in the above RT-PCR was used. The first cDNA strand was synthesized from 0.55 μg of total RNA using ReverTra Ace -α- (Toyobo Co. Ltd.). Quantitative PCR was performed in triplicate, in a total reaction volume of 20 μL using SYBR Green Realtime PCR Master Mix -Plus- (Toyobo) and 1 μL cDNA per reaction, on a 96CFX (Bio-Rad Laboratories). The PCR protocol consisted of an initial cDNA denaturation (2 min at 95 °C), followed by 40 cycles of 95 °C for 5 s and 60 °C for 30 s. Primers for amplification were as follows: LC sense primer located in LC open-reading frame, 5'-GGAGCTAGGTGCGACGTCGTTA-3'; LC antisense primer located in LC open-reading frame, 5'-TGAAAAGCGGTTGCTCACTCTGA-3'; HC sense primer located in intron-exon junction of HC, 5'-ACTCCCACGATCGCTCGTGA-3'; HC antisense primer located in HC open-reading frame, 5'-TGAAGCTAAAGCCAGAAGCTTTGC-3'. Each primer position was described in the below. Relative mRNA levels were calculated by the -ΔΔCt method.

Results

	LC Ct	HC Ct	ddCt	2(-ddCt)
Sample 1	21.80	23.72	-1.92	3.78
Sample 2	22.00	23.56	-1.56	2.95
Sample 3	22.04	23.58	-1.54	2.90

Average Ct ± SEM = 3.21 ± 0.28

The underlined areas indicate the positions of RT-PCR primers. The cyan-filled areas indicate the positions of real-time quantitative PCR primers. The green-filled areas indicate the CAE2_3.01 sequence that is removed as an intron.

LC mRNA

```
        10         20         30         40         50         60         70         80         90        100        110        120        130        140        150
gctctgactgaccgcgttactcccacgagtgagtttttccagccctgcatttttcagccatcctctttcactctacttttagccttggaagacagccgagagccaggccaagcactaggtgacagatatccaccATGGACATGCGGGTCC
                                                                                                                                               M  D  M  R  V  P
       160        170        180        190        200        210        220        230        240        250        260        270        280        290        300
CTGCCCAGCTTCTGGGCTTGCTGCTCCTGTGGGTGCCCGGAGCTAGGTGCGACGTCGTTATGACCCAGAGCCCACTGTCACTGCCCGTGACACTGGGACAACCTGCAAGCATATCCTGCAGATCTTCACAGAATATCGTGCATCAGAACG
 A  Q  L  L  G  L  L  L  L  W  V  P  G  A  R  C  D  V  V  M  T  Q  S  P  L  S  L  P  V  T  L  G  Q  P  A  S  I  S  C  R  S  S  Q  N  I  V  H  Q  N  G
       310        320        330        340        350        360        370        380        390        400        410        420        430        440        450
GGCAGACCTATCTTGGTTGGTACTTGCAGAAACCCGGGCAATCCCCTCAACTGCTGATCTTCAGAGTGAGCAACCGCTTTTCAGGCGTTCCTGATCGCTTTAGTGGCTCAGGATCTGGGACGGACTTTACCCTGAAGATTAGCCGTGTCG
 Q  T  Y  L  G  W  Y  L  Q  K  P  G  Q  S  P  Q  L  L  I  F  R  V  S  N  R  F  S  G  V  P  D  R  F  S  G  S  G  S  G  T  D  F  T  L  K  I  S  R  V  E
       460        470        480        490        500        510        520        530        540        550        560        570        580        590        600
AAGCCGAGGATGTTGGCGTCTACTACTGCTTCCAGGGTTCTCACATACCACGGACATTTGGTCAGGGAACAAGACTGGAGATCAAGAGGACTGTCGCTGCTCCGAGTGTGTTCATCTTTCCGCCATCCGATGAGCAGCTGAAGAGCGGAA
 A  E  D  V  G  V  Y  Y  C  F  Q  G  S  H  I  P  R  T  F  G  Q  G  T  R  L  E  I  K  R  T  V  A  A  P  S  V  F  I  F  P  P  S  D  E  Q  L  K  S  G  T
       610        620        630        640        650        660        670        680        690        700        710        720        730        740        750
CTGCATCTGTGGTCTGCTTGCTCAACAACTTCTATCCCAGGGAAGCTAAGGTGCAGTGGAAAGTGGACAATGCCCTGCAATCCGGCAATTCCCAGGAAAGCGTGACAGAACAGGACAGCAAGGATTCTACCTACTCCCTGTCAAGCACCC
 A  S  V  V  C  L  L  N  N  F  Y  P  R  E  A  K  V  Q  W  K  V  D  N  A  L  Q  S  G  N  S  Q  E  S  V  T  E  Q  D  S  K  D  S  T  Y  S  L  S  S  T  L
       760        770        780        790        800        810        820        830        840        850        860        870        880        890        900
TTACTCTGTCAAAGGCCGACTATGAGAAACACAAGGTGTATGCGTGTGAGGTGACCCATCAAGGCCTCAGTAGTCCCGTGACGAAAAGCTTCAATCGGGGAGAGTGTTAAgaattcacggtcactggccgcctccagtgcactcggaggt
 T  L  S  K  A  D  Y  E  K  H  K  V  Y  A  C  E  V  T  H  Q  G  L  S  S  P  V  T  K  S  F  N  R  G  E  C  *
       910        920        930        940        950        960        970        980        990       1000       1010       1020       1030       1040       1050
ggggtgtcttccacagtctcttttaacacatcccttcagctagttttgttgttcttttatttgacctaactttggcttttccgtttacagtcgctcgtgaggaattgtatactcgagccaccATGGACTGGACCTGGAGAATCCTCTTCC
                                                                                                                                      M  D  W  T  W  R  I  L  F  L
      1060       1070       1080       1090       1100       1110       1120       1130       1140       1150       1160       1170       1180       1190       1200
TCGTAGCAGCAGCTACAGGTGCACACAGCCAGGTTCAGCTTGTGCAGAGCGGAGCCGAGGTCAAGAAACCAGGCGCTTCTGTGAAAGTGTCATGCAAAGCTTCTGGCTTTAGCTTCACCACATACTGGATCACATGGGTGAGACAGGCTC
 V  A  A  A  T  G  A  H  S  Q  V  Q  L  V  Q  S  G  A  E  V  K  K  P  G  A  S  V  K  V  S  C  K  A  S  G  F  S  F  T  T  Y  W  I  T  W  V  R  Q  A  P
      1210       1220       1230       1240       1250       1260       1270       1280       1290       1300       1310       1320       1330       1340       1350
CCGGGCAAGGGCTGGAATGGATTGGAATGATACACCCCTCTGACGCCGAAACTCGACTTAGCCAGACTTTCAAGGACAAAGCCACACTGACCGCCGATAAGAGCACCAGCACCGCCTATATGGAGCTGAGTAGCCTGAGATCCGAGGATA
 G  Q  G  L  E  W  I  G  M  I  H  P  S  D  A  E  T  R  L  S  Q  T  F  K  D  K  A  T  L  T  A  D  K  S  T  S  T  A  Y  M  E  L  S  S  L  R  S  E  D  T
      1360       1370       1380       1390       1400       1410       1420       1430       1440       1450       1460       1470       1480       1490       1500
CCGCCGTCTATTATTGTGCGCGGGATTTCGACGTATGGGGTCAAGGGACTCTGGTTACTGTCTCAAGTGCGTCCACAAAGGGGCCTTCTGTGTTTCCTCTCGCTCCCTCCAGTAAGTCAACTAGCGGAGGAACTGCCGCTTTGGGGTGTC
 A  V  Y  Y  C  A  R  D  F  D  V  W  G  Q  G  T  L  V  T  V  S  S  A  S  T  K  G  P  S  V  F  P  L  A  P  S  S  K  S  T  S  G  G  T  A  A  L  G  C  L
      1510       1520       1530       1540       1550       1560       1570       1580       1590       1600       1610       1620       1630       1640       1650
TGGTGAAGGACTACTTTCCAGAACCAGTAACCGTATCTTGGAATTCTGGTGCTCTGACGAGCGGCGTGCATACCTTTCCTGCCGTGCTCCAGTCATCTGGTCTGTACTCCCTCTCAAGCGTAGTGACAGTTCCGAGTTCCAGTCTGGGCA
 V  K  D  Y  F  P  E  P  V  T  V  S  W  N  S  G  A  L  T  S  G  V  H  T  F  P  A  V  L  Q  S  S  G  L  Y  S  L  S  S  V  V  T  V  P  S  S  S  L  G  T
      1660       1670       1680       1690       1700       1710       1720       1730       1740       1750       1760       1770
CGCAGACATACATCTGCAATGTGAACCACAAACCCTCTAACACAAAAGTGGATAAGCGCGTCGAGCCTAAATCTTGCGACAAGACACACACTTGTCCGCCTTGTCCTGCACCAGAACTCCTGGGAGGTCCCAGCGTATTCCTGTTTCCTC
 Q  T  Y  I  C  N  V  N  H  K  P  S  N  T  K  V  D  K  R  V  E  P  K  S  C  D  K  T  H  T  C  P  P  C  P  A  P  E  L  L  G  G  P  S  V  F  L  F  P  P
CAAAGCCTAAGGACACCCTCATGATTTCACGGACACCCGAGGTTACATGCGTTGTGGTGGATGTGTCCCACGAAGATCCCGAAGTGAAATTCAACTGGTATGTGGATGGTGTTGAGGTACACAATGCGAAAACGAAACCCAGGGAAGAGC
 K  P  K  D  T  L  M  I  S  R  T  P  E  V  T  C  V  V  V  D  V  S  H  E  D  P  E  V  K  F  N  W  Y  V  D  G  V  E  V  H  N  A  K  T  K  P  R  E  E  Q
AGTACAACAGCACCTATAGGGTCGTCAGTGTCCTCACTGTGCTGCATCAGGACTGGTTGAATGGAAAGGAGTATAAATGCAAAGTCTCTAACAAGGCCCTTCCCGCACCAATCGAAAAGACCATCAGCAAAGCAAAGGGGCAACCGAGAG
 Y  N  S  T  Y  R  V  V  S  V  L  T  V  L  H  Q  D  W  L  N  G  K  E  Y  K  C  K  V  S  N  K  A  L  P  A  P  I  E  K  T  I  S  K  A  K  G  Q  P  R  E
AACCCCAGGTGTACACACTGCCACCTTCCCGCGAGGAGATGACCAAGAATCAGGTTAGCCTCACGTGTCTGGTTAAGGGCTTTTACCCATCTGACATTGCCGTCGAGTGGGAGTCAAATGGCCAACCCGAGAATAACTACAAAACCACCC
 P  Q  V  Y  T  L  P  P  S  R  E  E  M  T  K  N  Q  V  S  L  T  C  L  V  K  G  F  Y  P  S  D  I  A  V  E  W  E  S  N  G  Q  P  E  N  N  Y  K  T  T  P
CGCCTGTGTTGGACAGCGATGGCTCCTTCTTTCTGTATAGCAAGCTGACTGTCGACAAGAGCCGTTGGCAACAGGGGAATGTGTTCAGCTGCTCCGTGATGCATGAAGCACTTCATAACCACTACACTCAGAAATCCCTGTCCTTGAGTC
 P  V  L  D  S  D  G  S  F  F  L  Y  S  K  L  T  V  D  K  S  R  W  Q  Q  G  N  V  F  S  C  S  V  M  H  E  A  L  H  N  H  Y  T  Q  K  S  L  S  L  S  P
CAGGAAAGTAAgcggccgc
 G  K  *
```

HC mRNA

```
        10        20        30        40        50        60        70        80        90       100       110       120       130       140       150
gctctgactgaccgcgttactcccacgatcgctcgtgaggaattgtatactcgagccaccATGGACTGGACCTGGAGAATCCTCTTCCTCGTAGCAGCAGCTACAGGTGCACACAGCCAGGTTCAGCTTGTGCAGAGCGGAGCCGAGGTC
                          ▲ exon-intron junction            M  D  W  T  W  R  I  L  F  L  V  A  A  A  T  G  A  H  S  Q  V  Q  L  V  Q  S  G  A  E  V
       160       170       180       190       200       210       220       230       240       250       260       270       280       290       300
AAGAAACCAGGCGCTTCTGTGAAAGTGTCATGCAAAGCTTCTGGCTTTAGCTTCACCACATACTGGATCACATGGGTGAGACAGGCTCCCGGGCAAGGGCTGGAATGGATTGGAATGATACACCCCTCTGACGCCGAAACTCGACTTAGC
K  K  P  G  A  S  V  K  V  S  C  K  A  S  G  F  S  F  T  T  Y  W  I  T  W  V  R  Q  A  P  G  Q  G  L  E  W  I  G  M  I  H  P  S  D  A  E  T  R  L  S
       310       320       330       340       350       360       370       380       390       400       410       420       430       440       450
CAGACTTTCAAGGACAAAGCCACACTGACCGCCGATAAGAGCACCAGCACCGCCTATATGGAGCTGAGTAGCCTGAGATCCGAGGATACCGCCGTCTATTATTGTGCGCGGGATTTCGACGTATGGGGTCAAGGGACTCTGGTTACTGTC
Q  T  F  K  D  K  A  T  L  T  A  D  K  S  T  S  T  A  Y  M  E  L  S  S  L  R  S  E  D  T  A  V  Y  Y  C  A  R  D  F  D  V  W  G  Q  G  T  L  V  T  V
       460       470       480       490       500       510       520       530       540       550       560       570       580       590       600
TCAAGTGCGTCCACAAAGGGGCCTTCTGTGTTTCCTCTCGCTCCCTCCAGTAAGTCAACTAGCGGAGGAACTGCCGCTTTGGGGTGTCTGGTGAAGGACTACTTTCCAGAACCAGTAACCGTATCTTGGAATTCTGGTGCTCTGACGAGC
S  S  A  S  T  K  G  P  S  V  F  P  L  A  P  S  S  K  S  T  S  G  G  T  A  A  L  G  C  L  V  K  D  Y  F  P  E  P  V  T  V  S  W  N  S  G  A  L  T  S
       610       620       630       640       650       660       670       680       690       700       710       720       730       740       750
GGCGTGCATACCTTTCCTGCCGTGCTCCAGTCATCTGGTCTGTACTCCCTCTCAAGCGTAGTGACAGTTCCGAGTTCCAGTCTGGGCACGCAGACATACATCTGCAATGTGAACCACAAACCCTCTAACACAAAAGTGGATAAGCGCGTC
G  V  H  T  F  P  A  V  L  Q  S  S  G  L  Y  S  L  S  S  V  V  T  V  P  S  S  S  L  G  T  Q  T  Y  I  C  N  V  N  H  K  P  S  N  T  K  V  D  K  R  V
       760       770       780       790       800       810
GAGCCTAAATCTTGCGACAAGACACACACTTGTCCGCCTTGTCCTGCACCAGAACTCCTGGGAGGTCCCAGCGTATTCCTGTTTCCTCCAAAGCCTAAGGACACCCTCATGATTTCACGGACACCCGAGGTTACATGCGTTGTGGTGGAT
E  P  K  S  C  D  K  T  H  T  C  P  P  C  P  A  P  E  L  L  G  G  P  S  V  F  L  F  P  P  K  P  K  D  T  L  M  I  S  R  T  P  E  V  T  C  V  V  V  D
GTGTCCCACGAAGATCCCGAAGTGAAATTCAACTGGTATGTGGATGGTGTTGAGGTACACAATGCGAAAACGAAACCCAGGGAAGAGCAGTACAACAGCACCTATAGGGTCGTCAGTGTCCTCACTGTGCTGCATCAGGACTGGTTGAAT
V  S  H  E  D  P  E  V  K  F  N  W  Y  V  D  G  V  E  V  H  N  A  K  T  K  P  R  E  E  Q  Y  N  S  T  Y  R  V  V  S  V  L  T  V  L  H  Q  D  W  L  N
GGAAAGGAGTATAAATGCAAAGTCTCTAACAAGGCCCTTCCCGCACCAATCGAAAAGACCATCAGCAAAGCAAAGGGGCAACCGAGAGAACCCCAGGTGTACACACTGCCACCTTCCCGCGAGGAGATGACCAAGAATCAGGTTAGCCTC
G  K  E  Y  K  C  K  V  S  N  K  A  L  P  A  P  I  E  K  T  I  S  K  A  K  G  Q  P  R  E  P  Q  V  Y  T  L  P  P  S  R  E  E  M  T  K  N  Q  V  S  L
ACGTGTCTGGTTAAGGGCTTTTACCCATCTGACATTGCCGTCGAGTGGGAGTCAAATGGCCAACCCGAGAATAACTACAAAACCACCCCGCCTGTGTTGGACAGCGATGGCTCCTTCTTTCTGTATAGCAAGCTGACTGTCGACAAGAGC
T  C  L  V  K  G  F  Y  P  S  D  I  A  V  E  W  E  S  N  G  Q  P  E  N  N  Y  K  T  T  P  P  V  L  D  S  D  G  S  F  F  L  Y  S  K  L  T  V  D  K  S
CGTTGGCAACAGGGGAATGTGTTCAGCTGCTCCGTGATGCATGAAGCACTTCATAACCACTACACTCAGAAATCCCTGTCCTTGAGTCCAGGAAAGTAAgcggccgc
R  W  Q  Q  G  N  V  F  S  C  S  V  M  H  E  A  L  H  N  H  Y  T  Q  K  S  L  S  L  S  P  G  K  *  A  A
```

Supplementary Data 5

Plasmid map of used original vectors.

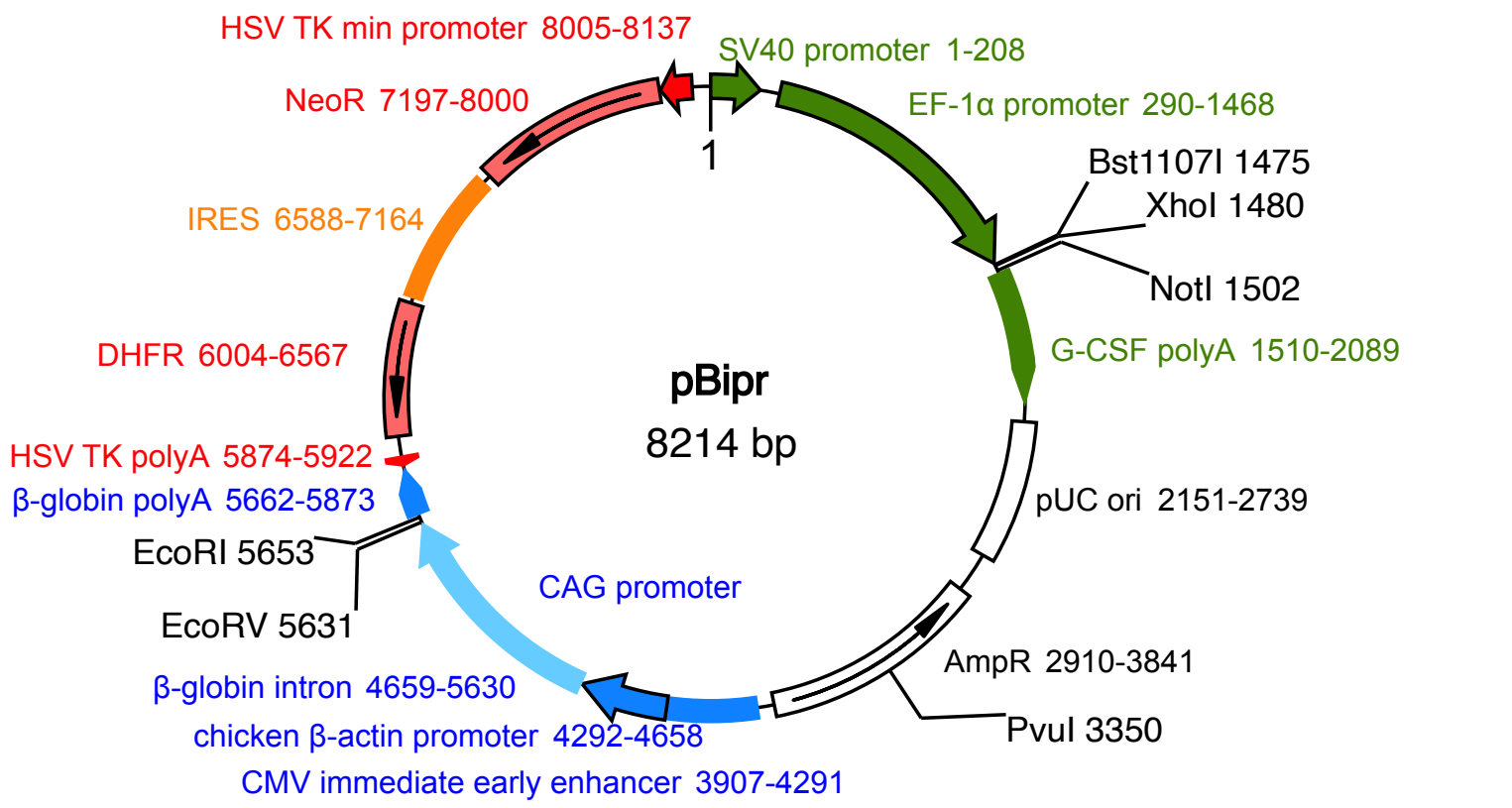

L-chain gene was inserted between *Eco*RV and *Eco*RI sites. H-chain gene was inserted between *Xho*I and *Not*I sites.

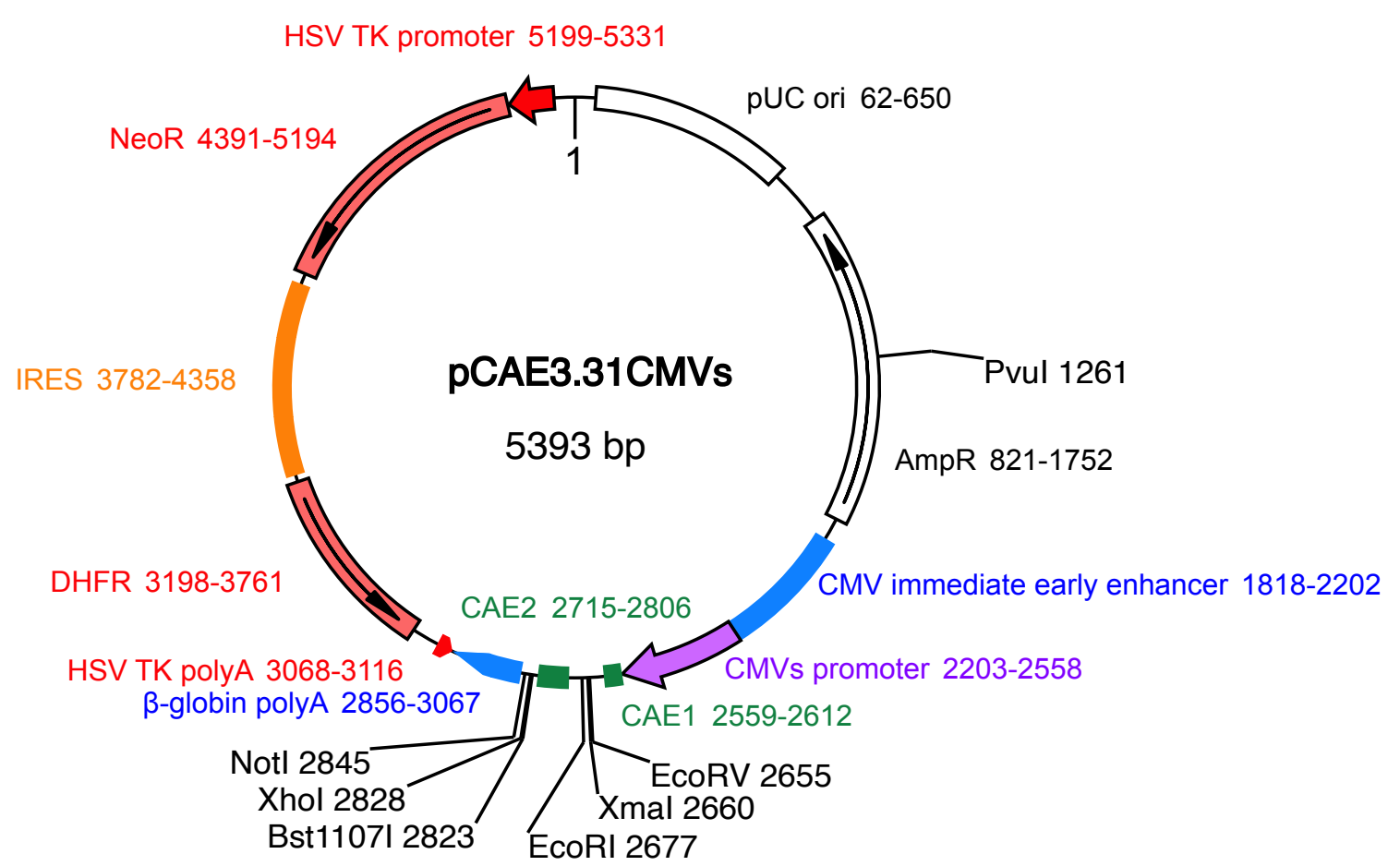

L-chain gene was inserted between *Eco*RV and *Eco*RI sites. H-chain gene was inserted between *Xho*I and *Not*I sites.

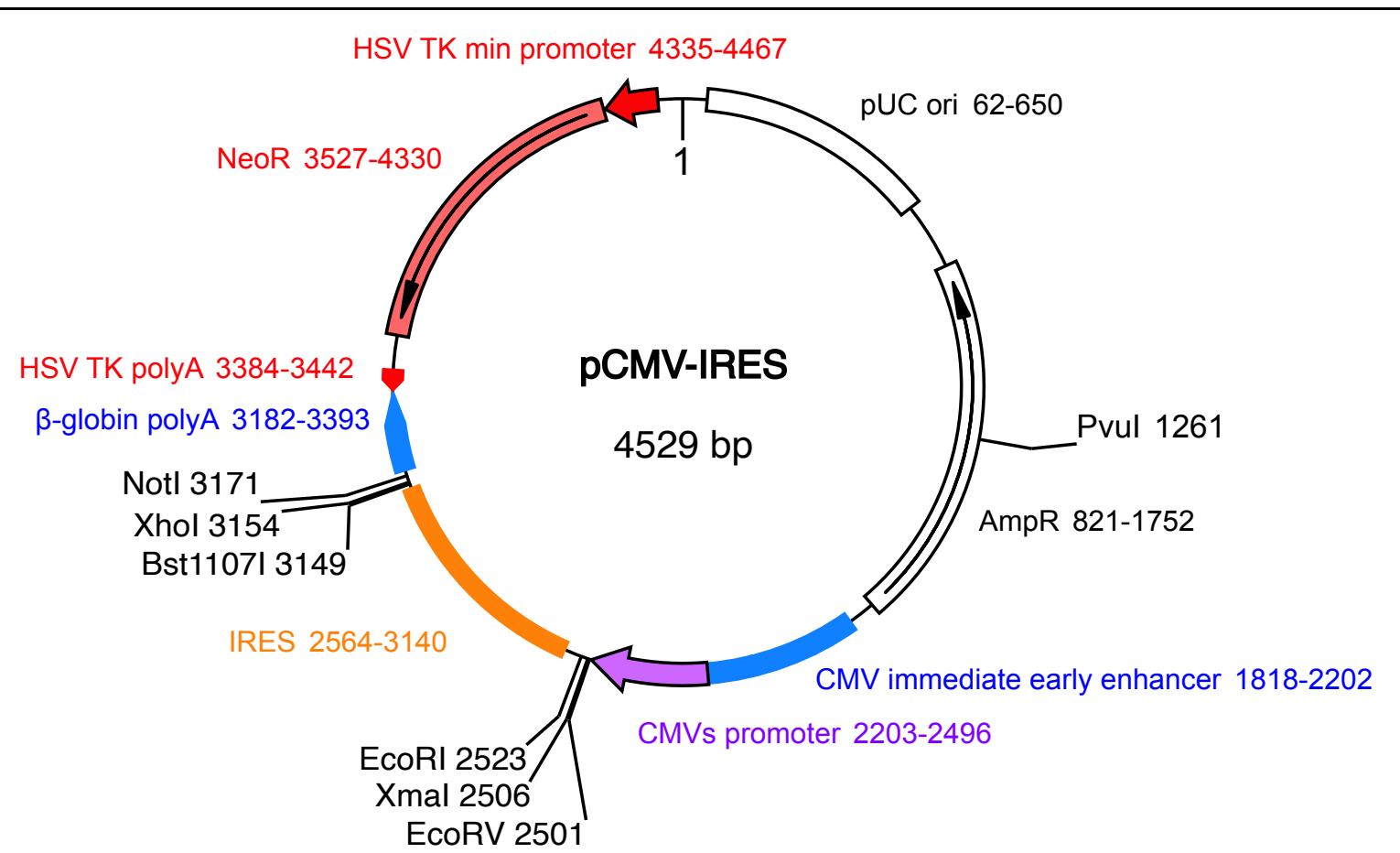

L-chain gene was inserted between *Eco*RV and *Eco*RI sites. H-chain gene was inserted between *Xho*I and *Not*I sites.

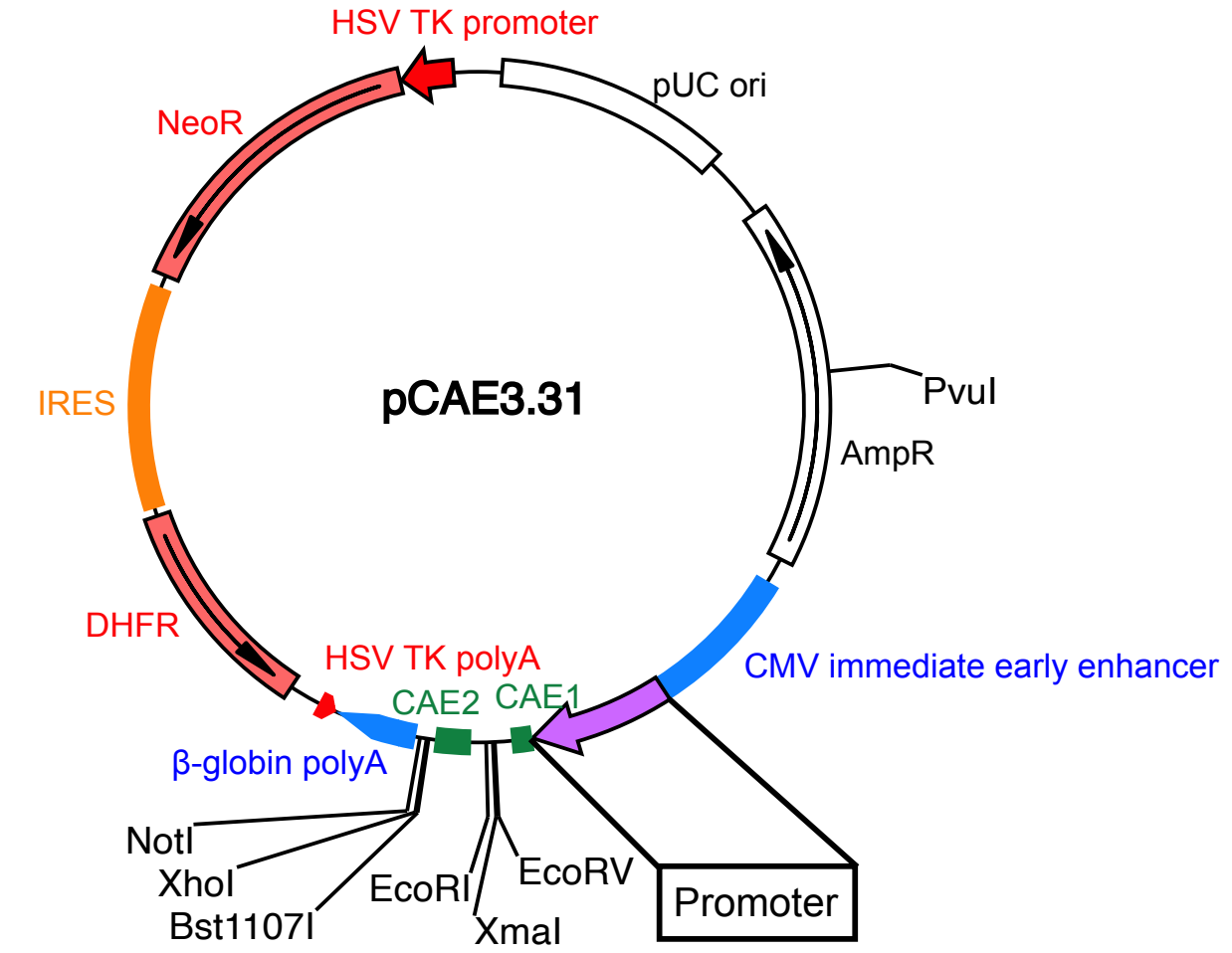	Promoter region of pCAE3.31 was replaced by CB, CMV, CMVs, or EF-1α promoter. L-chain gene was inserted between *Eco*RV and *Eco*RI sites. H-chain gene was inserted between *Xho*I and *Not*I sites.
CGAGGTGAGCCCCACGTTCTGCTTCACT<br>CTCCCCATCTCCCCCCCCTCCCCACCCC<br>CAATTTTGTATTTATTTATTTTTTAATTA<br>TTTTGTGCAGCGATGGGGGCGGGGGGG<br>GGGGGGCGCGCGCCAGGCGGGGCGGGG<br>CGGGGCGAGGGGCGGGGCGGGGCGAG<br>GCGGAGAGGTGCGGCGGCAGCCAATCA<br>GAGCGGCGCGCTCCGAAAGTTTCCTTTT<br>ATGGCGAGGCGGCGGCGGCGGCGGCCC<br>TATAAAAAGCGAAGCGCGCGGCGGGCG<br>GGAGTCGCTGCGCGCTGCCTTCGCCCCG<br>TGCCCCGCTCCGCCGCCGCCTCGCGCCG<br>CCCGCCCCGGCTCTGACTGACCGCGTTA<br>CTCCCACGA	CB promoter sequences
GATGCGGTTTTGGCAGTACATCAATGGG<br>CGTGGATAGCGGTTTGACTCACGGGGA<br>TTTCCAAGTCTCCACCCCATTGACGTCA<br>ATGGGAGTTTGTTTTGGCACCAAAATCA<br>ACGGGACTTTCCAAAATGTCGTAACAA<br>CTCCGCCCCATTGACGCAAATGGGCGGT<br>AGGCGTGTACGGTGGGAGGTCTATATA<br>AGCAGAGCTCGTTTAGTGAACCGTCAG<br>ATCGCCTGGAGACGCCATCCACGCTGTT<br>TTGACCTCCATAGAAGACACCGGGACC<br>GATCCAGCCTCCGGACTCTAGTCGCTGC<br>GCGCTGCCTTCGCCCCGTGCCCCGCTCC<br>GCCGCCGCCTCGCGCCGCCCGCCCCGGC<br>TCTGACTGACCGCGTTACTCCCACGA	CMV promoter sequences
GATGCGGTTTTGGCAGTACATCAATGGG	CMVs promoter sequences

CGTGGATAGCGGTTTGACTCACGGGGA<br>TTTCCAAGTCTCCACCCCATTGACGTCA<br>ATGGGAGTTTGTTTTGGCACCAAAATCA<br>ACGGGACTTTCCAAAATGTCGTAACAA<br>CTCCGCCCCATTGACGCAAATGGGCGGT<br>AGGCGTGTACGGTGGGAGGTCTATATA<br>AGCAGAGCTCGTTTAGTGAACCGTCAG<br>ATCGCCTGGAGACGCCATCCACGCTGTT<br>TTGACCTCCATAGAAGACACCGGGACC<br>GATCCAGCCTCCGGACTCTAGTCGCTGC<br>GCGCTGCCTCGCGCCGCCCGCCCCGGCT<br>CTGACTGACCGCGTTACTCCCACGA	
GGCTCCGGTGCCCGTCAGTGGGCAGAG<br>CGCACATCGCCCACAGTCCCCGAGAAG<br>TTGGGGGGAGGGGTCGGCAATTGAACC<br>GGTGCCTAGAGAAGGTGGCGCGGGGTA<br>AACTGGGAAAGTGATGTCGTGTACTGG<br>CTCCGCCTTTTTCCCGAGGGTGGGGGAG<br>AACCGTATATAAGTGCAGTAGTCGCCGT<br>GAACGTTCTTTTTCGCAACGGGTTTGCC<br>GCCAGAACACAGGCGCTGCGCGCTGCC<br>TCGCGCCGCCCGCCCCGGCTCTGACTGA<br>CCGCGTTACTCCCACGA	EF-1α promoter

Supplementary Data 6

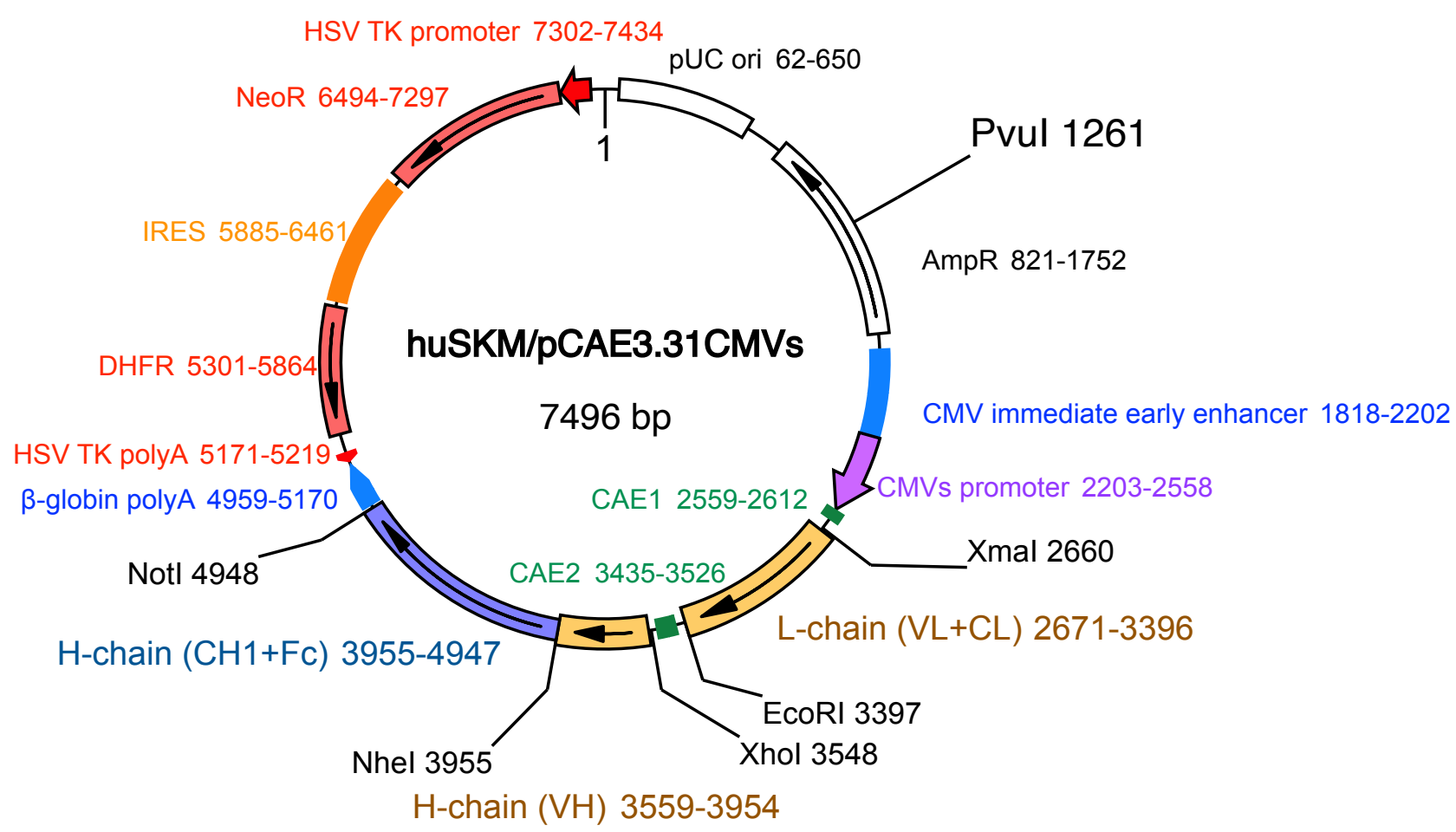

Sequences of humanized SKM9-2 in pCAE3.31CMVs. For other antibody production, blue sequences between Xma I and Nhe I was replaced with alternative antibody sequences.

```
        10        20        30        40        50        60        70        80        90       100       110       120
cccgggccaccATGGACATGCGGGTCCCTGCCCAGCTTCTGGGCTTGCTGCTCCTGTGGGTGCCCGGAGCTAGGTGCGACATCCAGATGACACAGTCCCCTTCCTCCTTGTCTGCATCTG
XmaI       M  D  M  R  V  P  A  Q  L  L  G  L  L  L  L  W  V  P  G  A  R  C  D  I  Q  M  T  Q  S  P  S  S  L  S  A  S  V
       130       140       150       160       170       180       190       200       210       220       230       240
TAGGCGATAGGGTGACCATCACATGTCGAAGTAGCCAGAACATCGTCCATAACAATGGCAACACATACCTGGGCTGGTATCAGCAGAAACCAGGTAAAGCTCCAAAGCTCCTGATCTTCA
  G  D  R  V  T  I  T  C  R  S  S  Q  N  I  V  H  N  N  G  N  T  Y  L  G  W  Y  Q  Q  K  P  G  K  A  P  K  L  L  I  F  K
       250       260       270       280       290       300       310       320       330       340       350       360
AGGTGAGCAATCGGTTCTCAGGAGTTCCTAGCCGCTTTTCTGGGTCAGGAAGTGGGACTGACTTCACTCTGACGATAAGCTCCCTTCAACCCGAAGATTTCGCCACCTATTACTGCTTTC
  V  S  N  R  F  S  G  V  P  S  R  F  S  G  S  G  S  G  T  D  F  T  L  T  I  S  S  L  Q  P  E  D  F  A  T  Y  Y  C  F  Q
       370       380       390       400       410       420       430       440       450       460       470       480
AGGGAAGTCACATTCCCAGAACCTTTGGTCAAGGCACCAAGGTGGAGATTAAGAGGACTGTCGCTGCTCCGAGTGTGTTCATCTTTCCGCCATCCGATGAGCAGCTGAAGAGCGGAACTG
  G  S  H  I  P  R  T  F  G  Q  G  T  K  V  E  I  K  R  T  V  A  A  P  S  V  F  I  F  P  P  S  D  E  Q  L  K  S  G  T  A
       490       500       510       520       530       540       550       560       570       580       590       600
CATCTGTGGTCTGCTTGCTCAACAACTTCTATCCCAGGGAAGCTAAGGTGCAGTGGAAAGTGGACAATGCCCTGCAATCCGGCAATTCCCAGGAAAGCGTGACAGAACAGGACAGCAAGG
  S  V  V  C  L  L  N  N  F  Y  P  R  E  A  K  V  Q  W  K  V  D  N  A  L  Q  S  G  N  S  Q  E  S  V  T  E  Q  D  S  K  D
       610       620       630       640       650       660       670       680       690       700       710       720
ATTCTACCTACTCCCTGTCAAGCACCCTTACTCTGTCAAAGGCCGACTATGAGAAACACAAGGTGTATGCGTGTGAGGTGACCCATCAAGGCCTCAGTAGTCCCGTGACGAAAAGCTTCA
  S  T  Y  S  L  S  S  T  L  T  L  S  K  A  D  Y  E  K  H  K  V  Y  A  C  E  V  T  H  Q  G  L  S  S  P  V  T  K  S  F  N
       730       740       750       760       770       780       790       800       810       820       830       840
ATCGGGGAGAGTGTTAAgaattcACGGTCACTGGCCGCCTCCAGTGCACTCGGAGGTGGGGTGTCTTCCACAGTCTCTTTTAACACATCCCTTCAGCTAGTTTTGTTGTTCTTTTATTTG
  R  G  E  C  *
       850       860       870       880       890       900       910       920       930       940       950       960
ACCTAACTTTGGCTTTTCCGTTTACAGTCGCTCGTGAGGAATTGTATActcgagccaccATGGACTGGACCTGGAGAATCCTCTTCCTCGTAGCAGCAGCTACAGGTGCACACAGCCAAG
                                                           M  D  W  T  W  R  I  L  F  L  V  A  A  A  T  G  A  H  S  Q  V
       970       980       990      1000      1010      1020      1030      1040      1050      1060      1070      1080
TGCAATTGCAGCAACCTGGCGCAGAACTGGTTCGACCTGGGGCAAGTGTCAAGCTGTCCTGTAAGGCCTCCGGGTTTTCCTTCACAACCTACTGGATAACATGGGTTAAGCAGCGTCCAG
  Q  L  Q  Q  P  G  A  E  L  V  R  P  G  A  S  V  K  L  S  C  K  A  S  G  F  S  F  T  T  Y  W  I  T  W  V  K  Q  R  P  G
      1090      1100      1110      1120      1130      1140      1150      1160      1170      1180      1190      1200
GGCAGGGCCTGGAGTGGATAGGGATGATTCATCCTAGTGACTCCGAGACAAGACTTTCACAGACCTTCAAGGACAAAGCTACGCTGACGGTTGATAAGTCCAGCAATACCGCCTACATGC
  Q  G  L  E  W  I  G  M  I  H  P  S  D  S  E  T  R  L  S  Q  T  F  K  D  K  A  T  L  T  V  D  K  S  S  N  T  A  Y  M  Q
      1210      1220      1230      1240      1250      1260      1270      1280      1290      1300      1310      1320
AGTTTTCCTCTCCAACCAGCGAGGATTCAGCTGTCTACTATTGCGTCCGGGATTTCGATGTATGGGGAGCTGGCACCACTGTTACCGTGTCAAGCgctagcACAAAGGGGCCTTCTGTGT
  F  S  S  P  T  S  E  D  S  A  V  Y  Y  C  V  R  D  F  D  V  W  G  A  G  T  T  V  T  V  S  S  A  S  T  K  G  P  S  V  F
                                                                                               NheI
```

```
      1330      1340      1350      1360      1370      1380      1390      1400      1410      1420      1430      1440
TTCCTCTCGCTCCCTCCAGTAAGTCAACTAGCGGAGGAACTGCCGCTTTGGGGTGTCTGGTGAAGGACTACTTTCCAGAACCAGTAACCGTATCTTGGAACTCTGGTGCTCTGACGAGCG
 P  L  A  P  S  S  K  S  T  S  G  G  T  A  A  L  G  C  L  V  K  D  Y  F  P  E  P  V  T  V  S  W  N  S  G  A  L  T  S  G
      1450      1460      1470      1480      1490      1500      1510      1520      1530      1540      1550      1560
GCGTGCATACCTTTCCTGCCGTGCTCCAGTCATCTGGTCTGTACTCCCTCTCAAGCGTAGTGACAGTTCCGAGTTCCAGTCTGGGCACGCAGACATACATCTGCAATGTGAACCACAAAC
 V  H  T  F  P  A  V  L  Q  S  S  G  L  Y  S  L  S  S  V  V  T  V  P  S  S  S  L  G  T  Q  T  Y  I  C  N  V  N  H  K  P
      1570      1580      1590      1600      1610      1620      1630      1640      1650      1660      1670      1680
CCTCTAACACAAAAGTGGATAAGCGCGTCGAGCCTAAATCTTGCGACAAGACACACACTTGTCCGCCTTGTCCTGCACCAGAACTCCTGGGAGGTCCCAGCGTATTCCTGTTTCCTCCAA
 S  N  T  K  V  D  K  R  V  E  P  K  S  C  D  K  T  H  T  C  P  P  C  P  A  P  E  L  L  G  G  P  S  V  F  L  F  P  P  K
      1690      1700      1710      1720      1730      1740      1750      1760      1770      1780      1790      1800
AGCCTAAGGACACCCTCATGATTTCACGGACACCCGAGGTTACATGCGTTGTGGTGGATGTGTCCCACGAAGATCCCGAAGTGAAATTCAACTGGTATGTGGATGGTGTTGAGGTACACA
 P  K  D  T  L  M  I  S  R  T  P  E  V  T  C  V  V  V  D  V  S  H  E  D  P  E  V  K  F  N  W  Y  V  D  G  V  E  V  H  N
      1810      1820      1830      1840      1850      1860      1870      1880      1890      1900      1910      1920
ATGCGAAAACGAAACCCAGGGAAGAGCAGTACAACAGCACCTATAGGGTCGTCAGTGTCCTCACTGTGCTGCATCAGGACTGGTTGAATGGAAAGGAGTATAAATGCAAAGTCTCTAACA
 A  K  T  K  P  R  E  E  Q  Y  N  S  T  Y  R  V  V  S  V  L  T  V  L  H  Q  D  W  L  N  G  K  E  Y  K  C  K  V  S  N  K
      1930      1940      1950      1960      1970      1980      1990      2000      2010      2020      2030      2040
AGGCCCTTCCCGCACCAATCGAAAAGACCATCAGCAAAGCAAAGGGGCAACCGAGAGAACCCCAGGTGTACACACTGCCACCTTCCCGCGAGGAGATGACCAAGAATCAGGTTAGCCTCA
 A  L  P  A  P  I  E  K  T  I  S  K  A  K  G  Q  P  R  E  P  Q  V  Y  T  L  P  P  S  R  E  E  M  T  K  N  Q  V  S  L  T
      2050      2060      2070      2080      2090      2100      2110      2120      2130      2140      2150      2160
CGTGTCTGGTTAAGGGCTTTTACCCATCTGACATTGCCGTCGAGTGGGAGTCAAATGGCCAACCCGAGAATAACTACAAAACCACCCCGCCTGTGTTGGACAGCGATGGCTCCTTCTTTC
 C  L  V  K  G  F  Y  P  S  D  I  A  V  E  W  E  S  N  G  Q  P  E  N  N  Y  K  T  T  P  P  V  L  D  S  D  G  S  F  F  L
      2170      2180      2190      2200      2210      2220      2230      2240      2250      2260      2270      2280
TGTATAGCAAGCTGACTGTCGACAAGAGCCGTTGGCAACAGGGGAATGTGTTCAGCTGCTCCGTGATGCATGAAGCACTTCATAACCACTACACTCAGAAATCCCTGTCCTTGAGTCCAG
 Y  S  K  L  T  V  D  K  S  R  W  Q  Q  G  N  V  F  S  C  S  V  M  H  E  A  L  H  N  H  Y  T  Q  K  S  L  S  L  S  P  G
      2290
GAAAGTAAgcggccgc
 K  *  NotI
```

Supplementary Data 7

Sequences design to avoid the formation of cryptic acceptor. Magenta indicates putative acceptor and branch point.

1) Signal peptide regions are often rich in leucine and phenylalanine residues encoded by C- or T-rich codons, making them prone to generating cryptic acceptor sites. When designing synthetic genes, cryptic splicing risk can be avoided by using an LC signal peptide that does not contain consecutive C/T-rich regions followed by AG dinucleotides. The effect on expression is shown in Figure 7b.

・Risky sequences（antibody expression was low.）

```
bHV1: ccaccATGGACTGGACCTGGAGAATCCTCTTCCTCGTAGCAGCAGCTACAGGTGCACACAGC
         M  D  W  T  W  R  I  L  F  L  V  A  A  A  T  G  A  H  S

moH1: ccaccATGGGATGGAGCTCTATCATCCTCTTCTTGGTAGCAACAACTACAGGTGTCCACTCC
         M  G  W  S  S  I  I  L  F  L  V  A  T  T  T  G  V  H  S
```

・Verified safe sequences

```
aKL1: ccaccATGGACATGCGGGTCCCTGCCCAGCTTCTGGGCTTGCTGCTCCTGTGGGTGCCCGGAGCTAGGTGC
         M  D  M  R  V  P  A  Q  L  L  G  L  L  L  L  W  V  P  G  A  R  C

cKL2: ccaccATGAGGTTGCCTGCCCAGCTGCTTGGGCTGCTCATGCTGTGGGTGCCCGGAAGCTCCGGC
         M  R  L  P  A  Q  L  L  G  L  L  M  L  W  V  P  G  S  S  G
```

2) Ser/Pro/Leu-rich motifs (yellow region) in the VL framework 1 region may form pseudo-acceptor motifs. Minimizing CT-rich codons and avoiding downstream AG dinucleotides can mitigate this risk.

Trastuzumab framework 1 (+signal peptide):

・Risky sequences (irregular splicing was detected by sequence analysis)

```
ccaccATGGACATGCGGGTCCCTGCCCAGCTTCTGGGCTTGCTGCTCCTGTGGGTGCCCGGAGCTAGGTGCGACATCCAGATGACACAGTCCCCTTCCTCCTTGTCTGCATCAGTAGGCGATAGG
   M  D  M  R  V  P  A  Q  L  L  G  L  L  L  L  W  V  P  G  A  R  C  D  I  Q  M  T  Q  S  P  S  S  L  S  A  S  V  G  D  R
```

・Verified safe sequences

```
ccaccATGGACATGCGGGTCCCTGCCCAGCTTCTGGGCTTGCTGCTCCTGTGGGTGCCCGGAGCTAGGTGCGACATCCAGATGACACAGTCCCCTTCCTCCTTGTCTGCATCTGTAGGCGATAGG
   M  D  M  R  V  P  A  Q  L  L  G  L  L  L  L  W  V  P  G  A  R  C  D  I  Q  M  T  Q  S  P  S  S  L  S  A  S  V  G  D  R
```

Ponezumab Framework 1（+signal peptide）：

・Risky sequences (irregular splicing was detected by sequence analysis)

```
ccaccATGGACATGCGGGTCCCTGCCCAGCTTCTGGGCTTGCTGCTCCTGTGGGTGCCCGGAGCTAGGTGCGACGTCGTTATGACCCAGTCTCCTCTGTCTCTCCCCGTTACACTTGGGCAGCCT
   M  D  M  R  V  P  A  Q  L  L  G  L  L  L  L  W  V  P  G  A  R  C  D  V  V  M  T  Q  S  P  L  S  L  P  V  T  L  G  Q  P
```

・Verified safe sequences

```
ccaccATGGACATGCGGGTCCCTGCCCAGCTTCTGGGCTTGCTGCTCCTGTGGGTGCCCGGAGCTAGGTGCGACGTCGTTATGACCCAGAGCCCACTGTCACTGCCCGTGACACTGGGACAACCT
   M  D  M  R  V  P  A  Q  L  L  G  L  L  L  L  W  V  P  G  A  R  C  D  V  V  M  T  Q  S  P  L  S  L  P  V  T  L  G  Q  P
```

Supplementary Data 8

a) The amino acid sequences of 762EGF. The sequences contain signal sequences derived from human HEG1 (1–28).

MASPRASRWPPPLLLLLLPLLLLPPAAPGTRDGTEKVITESKSPSLVSLPTESTGSQSP
TTLSSSASVNSCAVNPCLHNGECVADNTSRGYHCRCPPSWQGDDCSVDVNECLSN
PCPSTAMCNNTQGSFICKCPVGYQLEKGICNLVRTFVTEFKLKRTFLNTTVEKHSDL
QEVENEITKTLNMCFSALPSYIRSTVHASRESNAVVISLQTTFSLASNVTLFDLADR
MQKCVNSCKSSAEVCQLLGSQRRIFRAGSLCKRKSPECDKDTSICTDLDGVALCQC
KSGYFQFNKMDHSCRACEDGYRLENETCMSCPFGLGGLNCGNPYQGSDYKDDDD
KHHHHHH

b) The chromatogram of Mono Q purification and the dot blot image using SKM9-2 of eluted fraction. A black bar indicates pooled fractions.

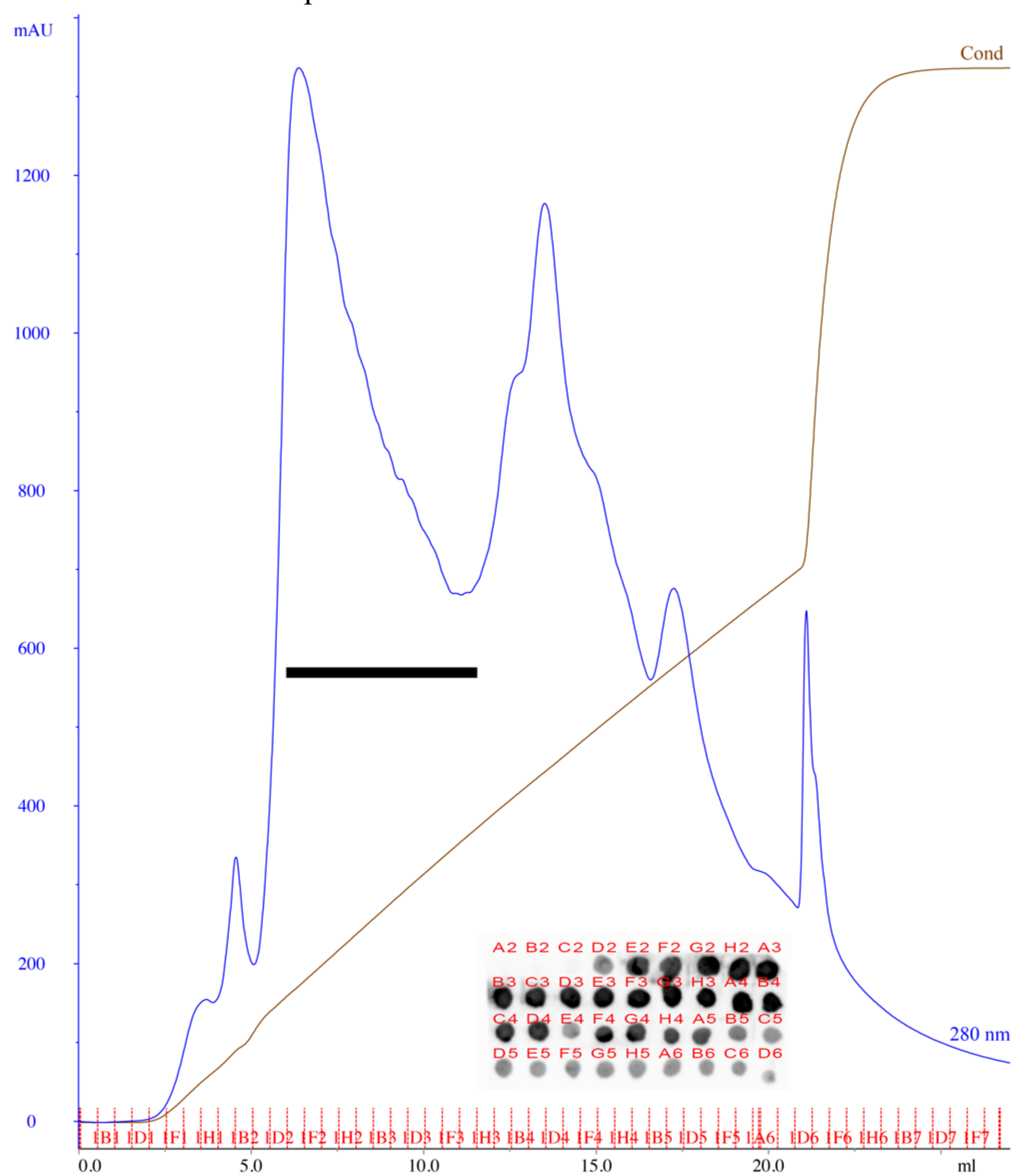

His-tagged recombinant ligand including SKM9-2 epitope (762EGF) was secreted by 293H cells stably expressing 762EGF. Culture supernatant (~800 mL) was filtered through Whatman GD/X syringe filters PVDF membrane (pore size 0.45 µm, Cytiva) and applied to Ni Sepharose excel column (1.6×6 cm) (Cytiva). After washing the column with 20 mM phosphate buffer (pH 7.4) containing 0.02% Tween 20 and 0.5 M NaCl, the sample was eluted with 20 mM phosphate buffer (pH 7.4) containing 0.5 M imidazole, 0.02% Tween 20, and 0.5 M NaCl. The eluate was dialyzed against 20 mM Tris buffer (pH 8.0) using Spectra/Por 3 dialysis tubing (Spectrum Laboratories, CA, USA). The 762EGF was further isolated by anion exchange chromatography using a Mono Q 5/50 GL column (Cytiva) connected to an AKTAexplorer 10S (Cytiva). The 762EGF was eluted in fractions E2–G3 with a linear gradient of 0–0.5 M NaCl in 20 mM Tris buffer (pH 8.0) and was strongly detected by dot blot analysis using SKM9-2. The detected fractions were pooled and stored at -80ºC.

Supplementary Data 9

A representative standard cure in ELISA. Purified SKM9-2 was used as a standard.

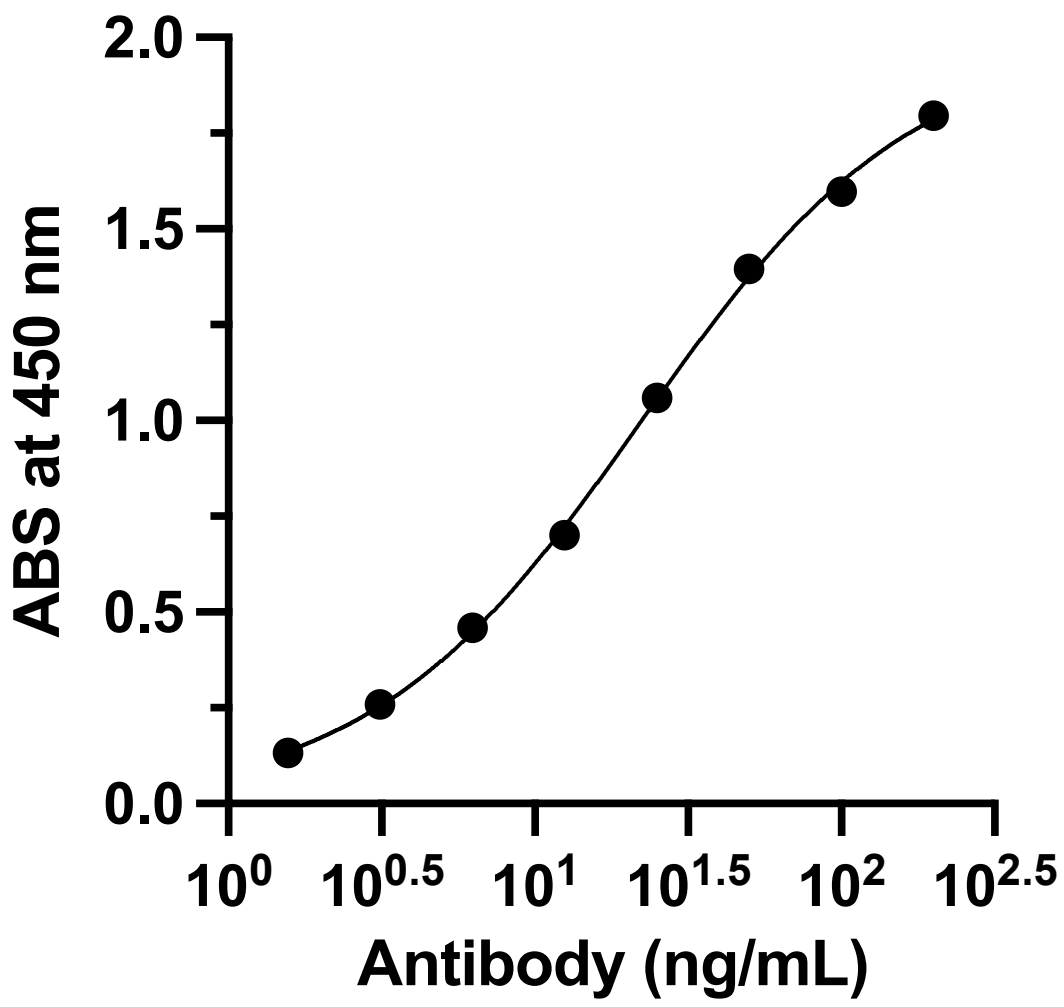

Supplementary Data 10

Sequences of mutants used in figures 5 and 6 were shown (from CAE1 to CAE2). L-chain sequences were shown in Supplementary data 2.

pCAE3.31:
GTGAGTTTTTCCAGCCCTGCATTTTTCAGCCATCCTCTTTCACTCTACTTTTAGC
CTTGGAAGACAGCCGAGAGCCAGGCCAAGCACTAGGTGACAGATATCCACC
[L-chain]
GAATTCACGGTCACTGGCCGCCTCCAGTGCACTCGGAGGTGGGGTGTCTTCCAC
AGTCTCTTTTAACACATCCCTTCAGCTAGTTTTGTTGTTCTTTTATTTGACCTAA
CTTTGGCTTTTCCGTTTACAG

del 3.61:
GTGAGTTTTTCCAGCCCTGCATTTTTCAGCCATCCTCTTTCACTCTACTTTTAGC
CTTGGAAGACAGCCGATATCCACC
[L-chain]
GAATTCCAGTGCACTCGGAGGTGGGGTGTCTTCCACAGTCTCTTTTAACACATC
CCTTCAGCTAGTTTTGTTGTTCTTTTATTTGACCTAACTTTGGCTTTTCCGTTTAC
AG

del 3.62:
GTGAGTTTTTCCAGCCCTGCATTTTTCAGCCATCCTCTTTCAGATATCCACC
[L-chain]
GAATTCTTTTAACACATCCCTTCAGCTAGTTTTGTTGTTCTTTTATTTGACCTAA
CTTTGGCTTTTCCGTTTACAG

del 3.63:
GTGAGTTTTTCCAGCCCTGCATTTTTCAGATATCCACCC
[L-chain]
GAATTCAGCTAGTTTTGTTGTTCTTTTATTTGACCTAACTTTGGCTTTTCCGTTTA
CAG

del 3.64:
GTGAGTTTTTCCAGATATCCACC
[L-chain]
GAATTCTTTTATTTGACCTAACTTTGGCTTTTCCGTTTACAG

del 4.2 :
GTGAGTTTTTCCAGATATCCACC
[L-chain]
GAATTCACCTAACTTTGGCTTTTCCGTTTACAG

mTC:

GTGAGTTTTTCCAGCCCTGCATTTTTCAGCCATCCTCTTTCACTCTACTTTTTCCC
TTGGAAGACAGCCGAGAGCCAGGCCAAGCACTAGGTGACAGATATCCACC
[L-chain]
GAATTCACGGTCACTGGCCGCCTCCAGTGCACTCGGAGGTGGGGTGTCTTCCAC
AGTCTCTTTTAACACATCCCTTCAGCTAGTTTTGTTGTTCTTTTATTTGACCTAA
CTTTGGCTTTTCCGTTTACAG

mFG:
GTGAGTTTTTCCAGCCCTGCTAACCATGTTCATGCCTTCTTCTTTTTCCTACAGC
CTTGGAAGACAGCCGAGAGCCAGGCCAAGCACTAGGTGACAGATATCCACC
[L-chain]
GAATTCACGGTCACTGGCCGCCTCCAGTGCACTCGGAGGTGGGGTGTCTTCCAC
AGTCTCTTTTAACACATCCCTTCAGCTAGTTTTGTTGTTCTTTTATTTGACCTAA
CTTTGGCTTTTCCGTTTACAG

mBG:
GTGAGTTTTTCCAGCCCTGCATTTTTCAGCCATCCTCTTTCACTCTACTTTTAGC
CTTGGAAGACAGCCGAGAGCCAGGCCAAGCACTAGGTGACAGATATCCACC
[L-chain]
GAATTCACGGTCACTGGCCGCCTCCAGTGCACTCGGAGGTGGGGTGTCTTCCAC
AGTCTCTTTTAACACATCCCTTCAGCTAGTTTTGTTGTTTGCTAACCATGTTCAT
GCCTTCTTCTTTTTCCTACAG

mTCBG:
GTGAGTTTTTCCAGCCCTGCATTTTTCAGCCATCCTCTTTCACTCTACTTTTTCCC
TTGGAAGACAGCCGAGAGCCAGGCCAAGCACTAGGTGACAGATATCCACC
[L-chain]
GAATTCACGGTCACTGGCCGCCTCCAGTGCACTCGGAGGTGGGGTGTCTTCCAC
AGTCTCTTTTAACACATCCCTTCAGCTAGTTTTGTTGTTTGCTAACCATGTTCAT
GCCTTCTTCTTTTTCCTACAG

mCA
GTGAGTTTTTCCAGCCCTGCATTTTTCAGCCATCCTCTTTCACTCTACTTTTAGC
CTTGGAAGACAGCCGAGAGCCAGGCCAAGCACTAGGTGACAGATATCCACC
[L-chain]
GAATTCACGGTCACTGGCCGCCTCCAGTGCACTCGGAGCAGGGGTGTCTTCCAC
AGTCTCTTTTAACACATCCCTTCAGCTAGTTTTGTTGTTCTTTTATTTGACCTAA
CTTTGGCTTTTCCGTTTACAG

mCG:
GTGAGTTTTTCCAGCCCTGCATTTTTCAGCCATCCTCTTTCACTCTACTTTTAGC
CTTGGAAGACAGCCGAGAGCCAGGCCAAGCACTAGGTGACAGATATCCACC
[L-chain]
GAATTCACGGTCACTGGCCGCCTCCAGTGCCCAGGGAGGTGGGGTGTCTTCCA

CAGTCTCTTTTAACACATCCCTTCAGCTAGTTTTGTTGTTCTTTTATTTGACCTA
ACTTTGGCTTTTCCGTTTACAG

3.33:
GTGAGTTTTTCCAGCCCTGCATTTTTCAGCCATCCTCTTTCACTCTACTTTTAGC
CTTGGAAGACAGCCGAGAGCCAGGCCAAGCACTAGGTGACAGATATCCACC
[L-chain]
GAATTCACGGTCACTGGCCGCCTCCAGTGCACTCGGAGGTGGGGTGTCTTCCAC
AGTTCTTTTATTTGACCTAACTTTGGCTTTTCCGTTTACAG

Supplementary Data 11

Full-length gel image of the agarose gel electrophoresis shown in Figure 4. Red dotted boxes indicate the cropped regions.

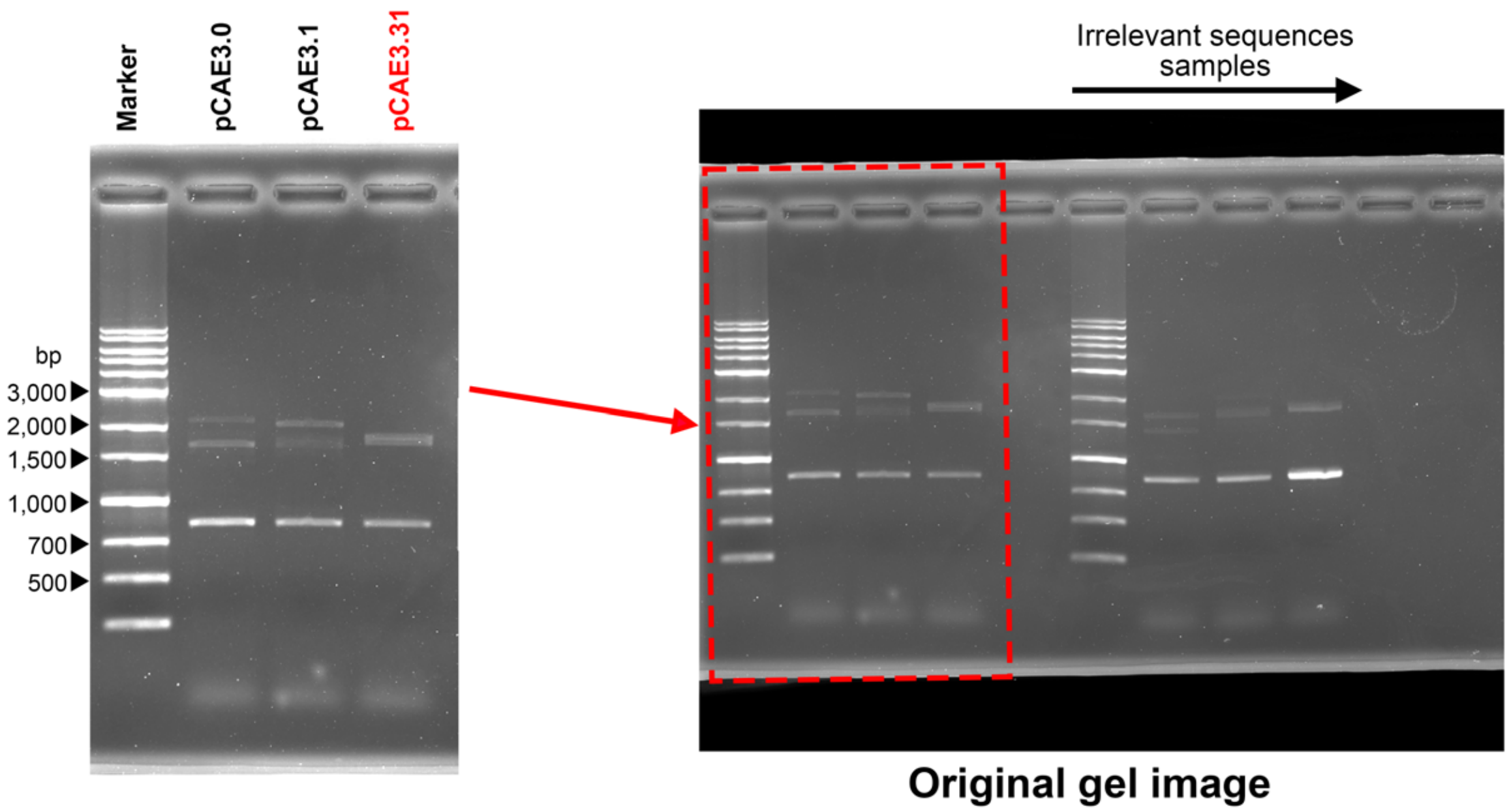

Supplementary Data 12

Full-length gel image of the SDS-PAGE shown in Figure 9. Red dotted boxes indicate the cropped regions.

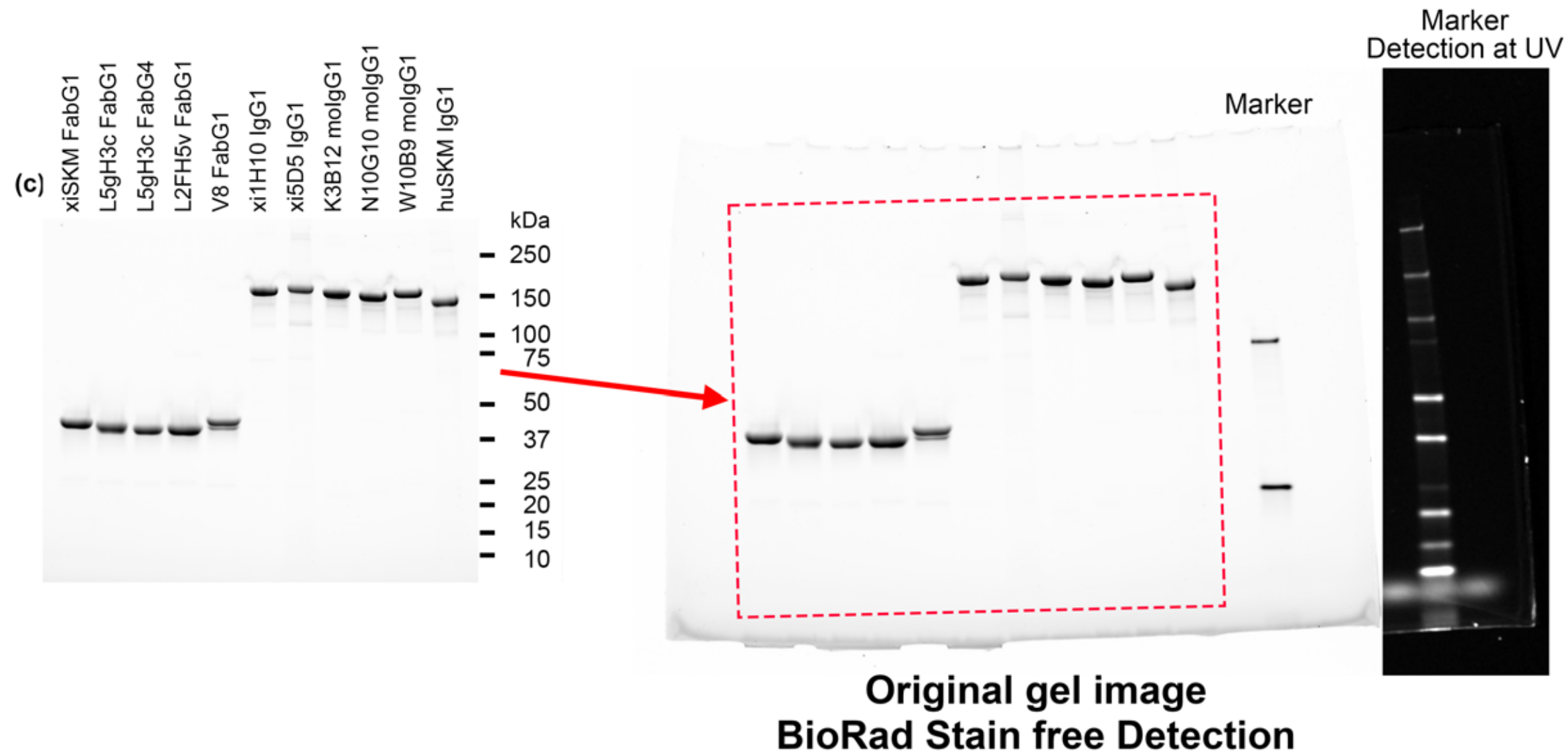

Supplement: Supplementary file 1 [file 41598_2026_57819_MOESM1_ESM.pdf]
